# Reactivity of a heterobinuclear heme–peroxo–Cu complex with *para*-substituted catechols shows a p*K*_a_-dependent change in mechanism†

**DOI:** 10.1039/d4sc05623j

**Published:** 2024-12-30

**Authors:** Sanjib Panda, Suzanne M. Adam, Hai Phan, Patrick J. Rogler, Pradip Kumar Hota, Joshua R. Helms, Brad S. Pierce, Gayan B. Wijeratne, Kenneth D. Karlin

**Affiliations:** a Department of Chemistry, Johns Hopkins University Baltimore Maryland 21218 USA karlin@jhu.edu; b Department of Chemistry & Biochemistry, The University of Alabama Tuscaloosa Alabama 35487 USA gwijeratne@ua.edu

## Abstract

In biological systems, heme–copper oxidase (HCO) enzymes play a crucial role in the oxygen reduction reaction (ORR), where the pivotal O–O bond cleavage of the (heme)Fe^III^–peroxo–Cu^II^ intermediate is facilitated by active-site (peroxo core) hydrogen bonding followed by proton-coupled electron transfer (PCET) from a nearby (phenolic) tyrosine residue. A useful approach to comprehend the fundamental relationships among H-bonding/proton/H-atom donors and their abilities to induce O–O bond homolysis involves the investigation of synthetic, bioinspired model systems where the exogenous substrate properties (such as p*K*_a_ and bond dissociation energy (BDE)) can be systematically altered. This report details the reactivity of a heme–peroxo–copper HCO model complex (LS-4DCHIm) toward a series of substituted catechol substrates that span a range of p*K*_a_ and O–H bond BDE values, exhibiting different reaction mechanisms. Considering their interactions with the bridging peroxo ligand in LS-4DCHIm, the catechol substrates are importantly capable of one or two (i) H-bonds, (ii) proton transfers, and/or (iii) net H-atom transfers, thereby making them attractive, yet complex candidates for studying the redox chemistry of the metal-bound peroxide. A combination of spectroscopic studies and kinetic analysis implies that the suitable modulation of p*K*_a_ and O–H bond BDE values of catechols result in either double proton transfer with the release of H_2_O_2_ or double PCET resulting in reductive O–O bond rupture. The distinguishing role of substrate properties in directing the mechanism and outcome of O_2_ protonation/reduction reactions is discussed in terms of designing O_2_-reduction catalysts based on biological inspiration.

## Abstract 2

Reactions of *para*-R-catechols with a cytochrome c oxidase synthetic model compound, a heme-peroxo-copper species, are p*K*_a_-dependent, suggesting O–O bond homolysis occurs *via* a hydrogen-bond assisted stepwise proton/electron transfer pathway.

## Graphical abstract

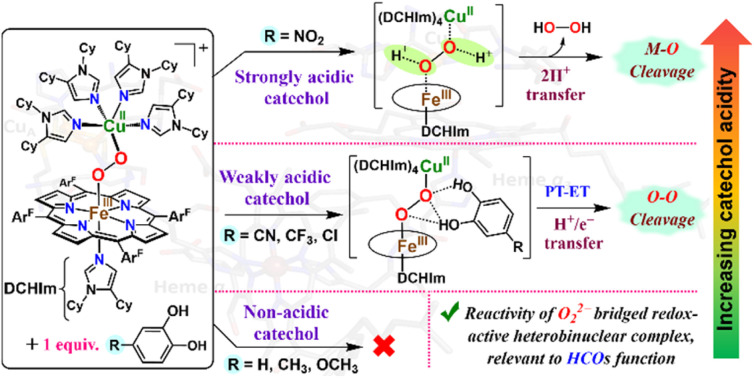

## Introduction

The controlled movement of protons and electrons in biological systems is of great fundamental and functional importance, as these chemical processes can occur in a variety of ways and are often the cornerstone of redox enzyme transformations. During the final step of cellular respiration, O–O bond reductive cleavage is effected at the heterobinuclear active site of heme–copper oxidases (HCOs), where two electrons from the heme a_3_ (Fe^II^ → Fe^IV^=O), one electron from the Cu_B_ site (Cu^I^ → Cu^II^–OH) and a single proton-coupled electron transfer (PCET) process from a juxtaposed tyrosine (Tyr → Tyr˙) residue are involved.^1–4^ Due to the importance of understanding O_2_ ⇄ H_2_O redox interconversion in the context of biology, as well as for fuel cell applications or catalytic oxidations,^5–12^ the mechanistic subtleties of how protons and electrons transfer most efficiently to accomplish O–O bond reductive cleavage (*i.e.*, concerted or sequentially) must be deciphered. In doing so, the acid strength (p*K*_a_), reduction potential and bond dissociation energy (BDE) of the relevant substrates should be balanced to achieve net H-atom donation to an O_2_-derived moiety. In thermodynamic terms, this can occur through various pathways under the broader scope of the proton-coupled electron transfer (PCET) umbrella, including PTET, ETPT or HAT (PT: proton transfer; ET: electron transfer; HAT: hydrogen atom transfer), which continues to attract significant interest from many research groups.^13–22^

**Fig. 1:**
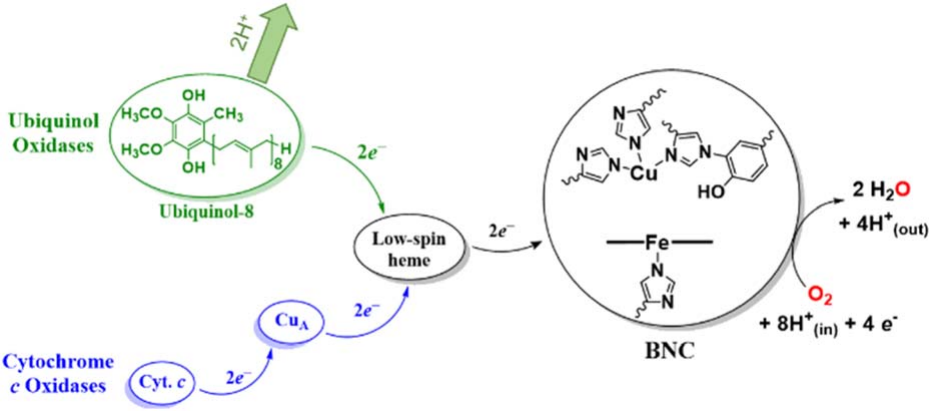
The flow of electrons in two types of HCOs. Quinol oxidases utilise a protein solubilised ubiquinol-8 (structure shown in green) as a source of two electrons, whereas C*c*Os receive reducing equivalents from cytochrome *c via* a dicopper site, Cu_A_. Both types of HCOs contain a low-spin heme cofactor, which shuttles electrons to the heme–copper active site where dioxygen is bound and reduced.

As relevant for this report, which describes PCET-mediated O–O bond cleavage of a (heme)Fe^III^–peroxo–Cu^II^(L) coordination complex, the heme–Cu binuclear center (BNC) in HCOs activates and reduces O_2_ by 4e^−^ (to H_2_O, Fig. 1).^2,3^ The most commonly studied HCOs are cytochrome *c* oxidases (C*c*Os), which receive reducing equivalents from cytochrome *c via* a dicopper cofactor, Cu_A_, and a low-spin heme site (Fig. 1, blue pathway). Most importantly, due to the fast kinetics of the O–O bond homolysis step in C*c*O enzymatic cycle, the fundamental details regarding the properties of putative metal-peroxide species (I_P_, documented to form before O–O bond cleavage) remain obscured.^23^ Extensive investigations using synthetic, spectroscopic, and computational models have provided substantial evidence for the presence of such an intermediate.^2,23–29^ Moreover, synthetic accessibility of (heme)Fe^III^–peroxo–Cu^II^ complexes (resembling I_P_) has resulted in their use as C*c*O models, which suggest that the O–O bond rupture in C*c*O most likely proceeds through a PT followed by ET from the Tyr.^2,23–26^

Another subfamily of HCOs, quinol oxidases (QOs), instead utilise a protein-solubilised ubiquinol (2,3-dimethoxy-5-methyl-6-polyprenyl-1,4-benzoquinol, Fig. 1, green) molecule as an electron source,^30^ where the two protons released following (PC)ET to the BNC are pumped to the intermembrane space (Fig. 1).^31,32^ In C*c*O and QO native enzymes, as well as in biomimetic systems from Collman^33^ and other research groups carrying out electrocatalytic O_2_-reduction of synthetic compounds,^2,28^ it has been shown that this process is controlled by the rate of electron flux to the active site, but also requires a network of H-bonding interactions to facilitate the PCET from a proximal phenolic moiety.^34–36^ Therefore, a fundamental understanding of the functional relationships between transition metal-O_2_ species and phenolic-type substrates remains to be of high importance.

**Fig. 2:**
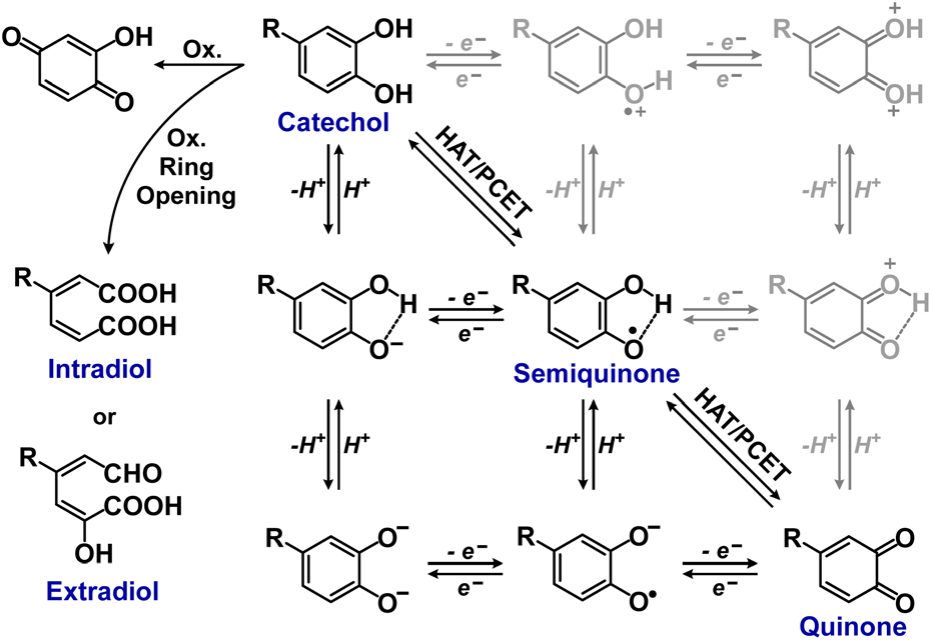
Square scheme and other oxidation pathways of *o*-catechols. The greyed cationic species are unlikely to be involved in PCET reactions due to their high-energy nature.^4^

Hydroquinones (*para*-dihydroxybenzenes) or catechols (*ortho*-dihydroxybenzenes) act as redox substrates in several biochemical/metalloenzyme reactions, most famously in quinol oxidase^37–40^ and catechol oxidase,^41–43^ respectively, where the reducing equivalents are directed toward O–O bond cleavage chemistry. In both of these cases, the hydroquinone or catechol undergoes 2-electron oxidation to form the corresponding quinone, most likely *via* a PCET-type process. Redox reactions of catechols in synthetic systems can be exceedingly complex to interpret because, depending on the catecholic ring substitution(s), a library of such substrates can span a wide range of p*K*_a_'s, O–H BDEs, and ionization potentials (IP) (for the first and second OH group; Fig. 2). Other mechanistic routes include only proton or only electron donation (although the latter is unlikely because oxidations without accompanying proton transfers yield high-energy species),^14^ as well as “overoxidation” to produce a hydroxy-quinone or ring-opened diols (Fig. 2). The overall PCET square scheme for 4-substituted catechols is shown in Fig. 2 and importantly includes potential internal H-bonding in certain deprotonated formulations.

**Fig. 3:**
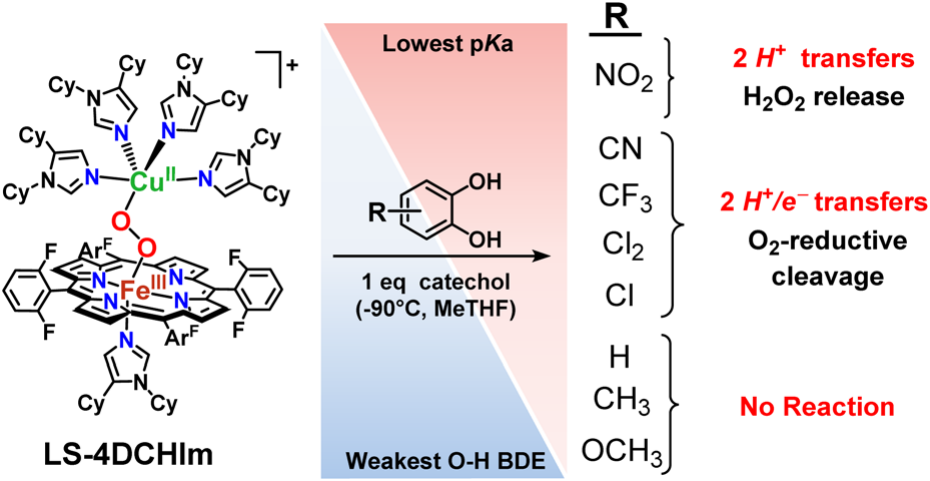
Reactions of the model heme–peroxo–copper complex (LS-4DCHIm) with substituted catechols reveal distinct mechanistic pathways. As dependent on the catechol substitution (with varying p*K*_a_ and BDE), H^+^ transfer occurs when R = NO_2_, net H-atom transfer when R = CN, CF_3_, Cl_2_, Cl, and no reaction when R = H, CH_3_, OCH_3_.

The bio-inspired model complex utilised in this study, LS-4DCHIm ([(DCHIm)(F_8_)Fe^III^–(O_2_^2−^)–Cu^II^(DCHIm)_4_]^+^; F_8_ = tetrakis(2,6-difluorophenyl)porphyrinate; DCHIm = 1,5-dicyclohexylimidazole) (Fig. 3), contains a low-spin heme-Fe^III^ bridged by a (*trans-μ*-1,2-O_2_^2−^) ligand to a Cu^II^ ion,^44^ where the axial ligand on the heme-iron and the four additional Cu ligands are monodentate imidazole (DCHIm) donors. The copper(ii) ion in LS-4DCHIm adopts a distorted square pyramidal geometry, with a DCHIm ligand occupying the axial position. Importantly, the independent nature of the monodentate ligands seemingly imparts a certain degree of flexibility in Cu–ligand geometry, allowing small rearrangements to accommodate substrates approaching the bridging peroxo to assist in activation and/or cleavage.^45^ Our lab has recently evaluated the reactivity of LS-4DCHIm toward separate H^+^/H-bond and electron sources,^45^ where a weak phenolic acid, 4-NO_2_-phenol, H-bonded to the copper-bound O-atom of the bridging peroxo moiety in LS-4DCHIm to generate an adduct with an activated O–O bond (UV-vis, rR, and density functional theory (DFT) evidence).^45^ Unlike the parent peroxo complex, the H-bonded adduct was observed to be reactive toward exogenous decamethylferrocene reductant (Fc*), and undergoes 2e^−^ reductive O–O bond cleavage.^45^ Therefore, the overall peroxo reduction process was essentially following a PT-ET mechanism which proceeded *via* a H-bonded adduct.

In this report, we expand our understanding of O_2_ activation and reduction by heme–copper systems related to biological O_2_-reduction. Our overarching interests lie in gaining a deeper understanding of the details of PCET-type reactions involving reductive O–O cleavage of dioxygen. Thus, by utilizing catechols, which can potentially H-bond in this system and provide one or two protons and/or electrons, we can monitor the effects of subtle changes in p*K*_a_, BDE, IP and H-bonding ability on the reaction outcomes with the LS-4DCHIm peroxo model system. Therefore, we have tested the reactivity of LS-4DCHIm toward a series of substituted catechols that span a range of p*K*_a_ and O–H BDE values (Fig. 3). This demonstrates how the p*K*_a_ of the catechol influences the reaction mechanism, such as: (i) for catechols with strongly electron-withdrawing NO_2_ group, the reaction proceeds *via* metal–oxygen bond cleavage (releasing H_2_O_2_); (ii) in contrast, catechols with moderately withdrawing groups (CN, CF_3_, Cl_2_, and Cl) favour O–O bond rupture; and (iii) catechols without substitution or with strongly electron-donating groups (OCH_3_ and CH_3_) do not react at all.

The results reported herein, garnered from a variety of spectroscopic and analytical methods, provide insights into the favourable substrate properties that lead to O–O bond cleavage. They also highlight the fact that subtle changes in chemical properties can have significant impacts on the mechanism and outcome of protonation/reduction reactions. Kinetic investigations and determination of the fate of the peroxo O-atoms (*i.e.*, giving H_2_O_2_ or water) following the catechol reactions allow for distinguishing between 2PT *vs.* PTET mechanistic proposals (Fig. 3). Relating the reaction outcomes to trends in thermodynamic parameters of the catechol substrates allows for semi-quantitation; these, we observe a p*K*_a_-dependent mechanism, rather than a BDE-dependent mechanism (*i.e.*, a rate-limiting proton transfer process), which leads us to offer new insights into the favourable conditions for O–O bond cleavage in such an environment.

## Results and discussion

### Reaction of LS-4DCHIm with 4-NO_2_-catechol (2PT)

**Fig. 4:**
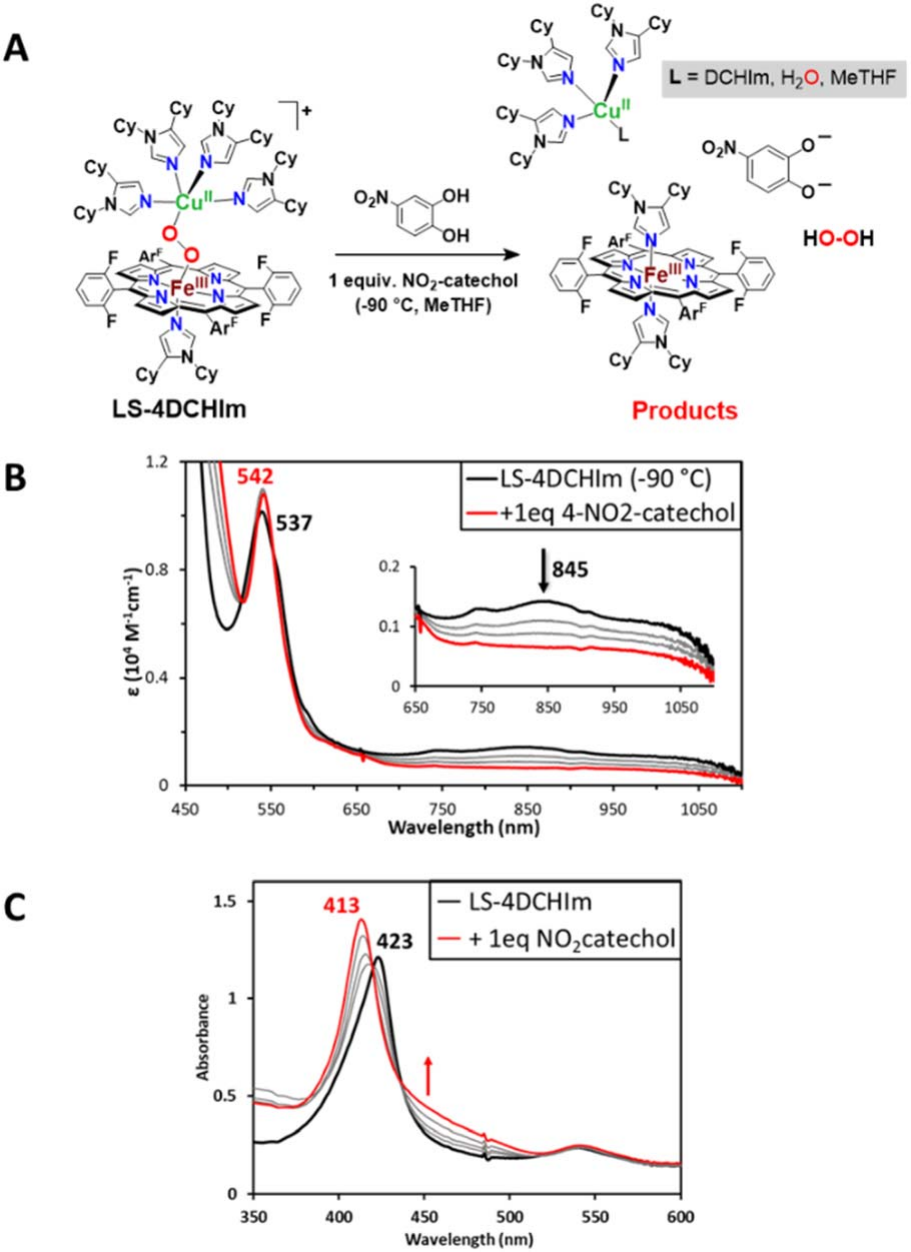
(A) Reaction scheme and (B) UV-vis spectral changes at the Q-band and low energy regions (0.1 mM, *l* = 1 cm) and the Soret band (C) (0.1 mM, *l* = 2 mm) which occur when 1 equiv. of 4-NO_2_-catechol is added to LS-4DCHIm (black spectra) to yield the products depicted in the top scheme (red spectra), including F_8_Fe^III^(DCHIm)_2_, Cu^II^(DCHIm)_4_ and 4-NO_2_-catecholate (see ESI†) (*l* = cuvette path length).

Perhaps the most suitable way to monitor reactions of the LS-4DCHIm complex is *via* UV-visible spectroscopy, which allows detection of even slight changes in the absorption profile at the heme Soret and Q-bands, as well as in the low energy features, and in some cases, allows for observation of organic reaction products. Addition of one equivalent of 4-NO_2_-catechol to a solution of LS-4DCHIm (*λ*_max_ = 423, 537, 845 nm, black spectra in Fig. 4B and C) at −90 °C induces a fast reaction as monitored by UV-vis spectroscopy (∼60 s) to yield the product spectra shown in red in Fig. 4B and C. The absorbance changes include a Q-band shift to 542 nm and isosbestic conversion of the Soret band to 413 nm. In addition, we observe a rapid decrease in intensity of the low energy features associated with the peroxo-to-Fe charge transfer transitions for heme–peroxo–Cu complexes in a low-spin iron(iii) environment, based on prior rR spectroscopy and DFT calculations (Fig. 4B and C).^44,46^ These observations suggest fragmentation of the heme–peroxo–Cu core structure and formation of the low-spin bis-imidazolyl F_8_Fe^III^(DCHIm)_2_ product (Fig. 4A), which has been separately generated and characterised (*λ*_max_ = 413, 542 nm).^45^ Additionally, the EPR spectrum of the product mixture (Fig. S1†) shows the individual oxidised metal complexes (Fig. 4A), Cu^II^(DCHIm)_4_ and F_8_Fe^III^(DCHIm)_2_. Authentic samples of both of these species can be generated, therefore spectral addition using calibration curves allows for semi-quantitation; these Fe^III^ and Cu^II^ products were both obtained in ∼75% yield (Fig. S1†).

**Fig. 5:**
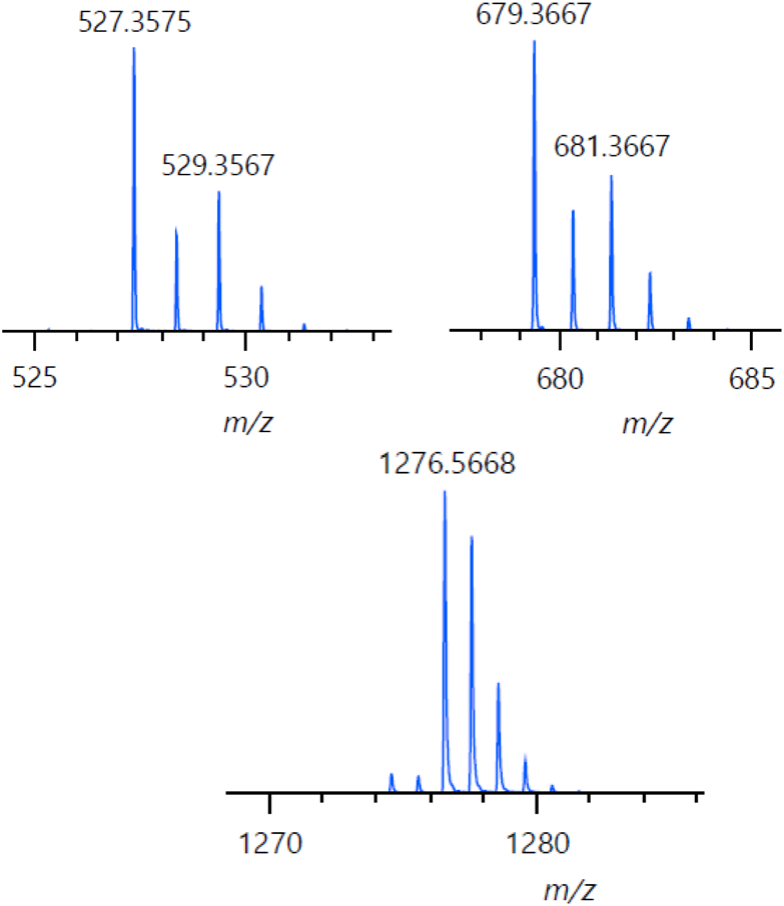
Experimental ESI(+)-MS (segment) of product mixture following the reaction of LS-4DCHIm with 4-NO_2_-catechol. Masses (*m*/*z*) at 527.36 ([Cu(DCHIm)_2_]^+^), 679.37 ([Cu(DCHIm)_2_(4-NO_2_-catecholate)]^+^, detected as [M–H]^+^), and 1276.57 ([F_8_Fe(DCHIm)_2_]^+^).

Indeed, the absorption features of the bis-imidazolyl heme product (*i.e.*, F_8_Fe^III^(DCHIm)_2_; where the DCHIm ligands are coordinated *trans*-axially) dominate the UV-vis spectrum of the final product mixture; however, we are aware that different mechanisms can lead to the same heme product formation,^45^ often preventing direct UV-vis observation of organic reaction products (catecholate or quinone) due to their low molar absorptivity (<4000 M^−1^ cm^−1^) and positioning of *λ*_max_ (400–440 nm, *i.e.*, underneath the intense heme Soret band). Nevertheless, in this case, an additional increase in absorbance is observed at ∼440 nm (appearing as a shoulder on the heme Soret band, indicated by a red arrow in Fig. 4C) in the UV-visible spectrum, which can be attributed to the 4-NO_2_-catecholate species which has been generated separately (Fig. 4C, and S2†). This absorbance increase corresponds to the formation of approximately one equivalent of 4-NO_2_-catecholate as determined by spectral matching to a 1 : 1 : 1 mixture of the products: F_8_Fe^III^(DCHIm)_2_, Cu^II^(DCHIm)_4_, and [4-NO_2_-catecholate]^2−^ (Fig. S2†). Therefore, the spectroscopic characterization of the products by UV-vis and EPR support the formation of the F_8_Fe^III^(DCHIm)_2_, Cu^II^(DCHIm)_4_, and catecholate products depicted in Fig. 4A. Consistent with these results, the ESI(+)-MS of the reaction mixture (Fig. 5 and S3†) exhibits a peak at *m*/*z* = 1276.57 corresponding to [F_8_Fe^III^(DCHIm)_2_]^+^. Additionally, the peak at 527.36 can be attributed to the stable reduced form of [Cu^II^(DCHIm)_4_]^2+^, *i.e.*, [Cu^I^(DCHIm)_2_]^+^, which is always observed due to the inherent reducing environment within the ESI-MS experiment. Also, the peak at 679.37 may suggest the partial formation of [(DCHIm)_2_Cu^II^(4-NO_2_-catecholate)]^+^, detected as [M−H]^+^ in the ESI-MS (the expected *m*/*z* for [M]^+^ is 680.38; see the ESI† for our further explanation) (Fig. 5). In conjunction with the UV-vis spectrum of free 4-NO_2_-catecholate (*vide supra*), the presence of a Cu^II^-catecholate moiety in the mass spectrum provides indirect evidence of catecholate formation as a by-product.

As further support for this conclusion, the established horseradish peroxidase (HRP) enzyme assay^45,47^ allows for quantification of the amount of H_2_O_2_ released in the reaction shown in Fig. 4A. This method shows that addition of one equivalent of 4-NO_2_-catechol to the LS-peroxo complex results in the evolution of a stoichiometric amount of H_2_O_2_ (100% yield, Table 1), implying that this acidic catechol acts as a 2H^+^-transfer reagent (2PT mechanism; p*K*_a_(1)[NO_2_-catechol] = 11.9, p*K*_a_(2)[NO_2_-monocatecholate]^1−^ = 31.4, *vide infra*). Interestingly, upon addition of 2 equiv. strong acid [DMF·H^+^](CF_3_SO_3_^−^) to the reaction product mixture, we observe the disappearance of the absorption feature (*λ*_max_ = 440 nm, *vide supra*) attributed to the catecholate, therefore, we believe that the stronger acid re-protonates the catecholate (Fig. S4†). Additionally, the generation of H_2_O_2_ in the {LS-4DCHIm + 4-NO_2_-catechol} reaction mixture has been supported by an iodide test (see Fig. S5†).

**Table 1:** Quantification of H_2_O_2_ evolved from reactions of LS-4DCHIm with catechols *via* the horseradish peroxidase assay

Substrate	H_2_O_2_ evolved^a^ (%)
4-NO_2_-catechol	100
4-CN-catechol	6
4-CF_3_-catechol	2
4,5-Cl_2_-catechol	5
4-Cl-catechol	6

a Quantification of H_2_O_2_ was achieved by recording the intensity of the diammonium 2,2′-azinobis(3-ethylbenzothiazoline-6-sulfonate) (AzBTS-(NH_4_)_2_) peaks (see ESI).

**Scheme 1:**
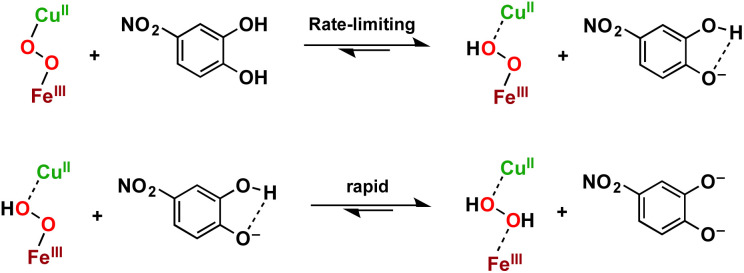
Stepwise transfer of two protons from 4-NO_2_-catechol to LS-4DCHIm, cleaving the metal–O bonds and releasing H_2_O_2_ without the involvement of any reducing equivalents.

It has previously been shown that, when added to various metal peroxo species, acids with sufficiently low p*K*_a_'s cause release of H_2_O_2_ following acid–base metal–oxygen bond cleavage.^45,48^ Accordingly, a catechol in which the first and second p*K*_a_ values are low enough (*i.e.*, lower than the p*K*_a_ of the conjugate acids of Fe–(O_2_^2−^)–Cu and Fe⋯(HO_2_^−^)⋯Cu, respectively), can act as a 2H^+^-donor, without providing any exogenous reducing equivalents. To this end, we conclude that 4-NO_2_-catechol, which is relatively acidic but difficult to oxidise (O–H BDE = 78.3 and 73.4), reacts as a 2-proton donor, cleaving metal–O bonds *via* acid–base chemistry and releasing H_2_O_2_, an undesirable ROS (Scheme 1).

### Reaction of LS-4DCHIm with 4-substituted –CN, –CF_3_, –Cl_2_, and –Cl catechols (2PTET)

**Fig. 6:**
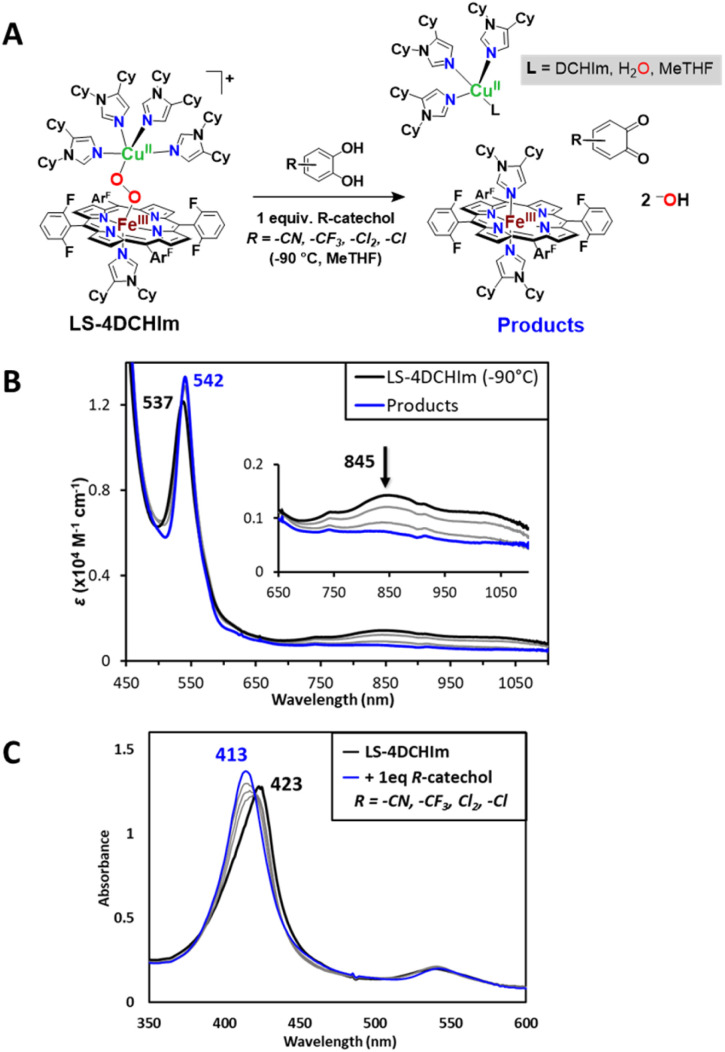
(A) Reaction scheme and (B) UV-vis spectral changes at the Q-band and low energy regions (0.1 mM, *l* = 1 cm) and the Soret band (C) (0.1 mM, *l* = 2 mm), which occur when 1 equiv. of the catechols shown in the scheme (R = –CN, –CF_3_, –Cl_2_, –Cl) are added to LS-4DCHIm (black spectra) to yield the products depicted in the top scheme (blue spectra), characteristic of the F_8_Fe^III^(DCHIm)_2_ product.

The addition of one equivalent of catechols featuring moderately to slightly electron-withdrawing substituents (–CN, –CF_3_, –Cl_2_, and –Cl catechols) to LS-4DCHIm at −90 °C likewise induces a reaction. Isosbestic conversion of the Soret band from 423 to 413 nm is accompanied by a shift in the Q-band from 537 to 542 nm, and the low energy features of LS-4DCHIm disappear (Fig. 6 and S9†). As in the case of 4-NO_2_-catechol, the final UV-vis spectrum is consistent with the formation of the heme species, F_8_Fe^III^(DCHIm)_2_ (*λ*_max_ = 413, 542 nm, Fig. 6B, C and S9†)^45^ in high yield. EPR spectroscopy (Fig. S6†) further supports a product mixture comprising the heme Fe^III^ and a Cu^II^ complex in a ∼1 : 1 ratio. In support of this conclusion, we measured the final Cu(ii) concentration in the {LS-4DCHIm + 4-Cl-catechol} reaction mixture; the results confirm the quantitative formation of Cu(ii) and also reveal a usual axial EPR pattern with hyperfine splitting, which is consistent with the simulated spectra (Fig. S6b†). Overall, the detection of the oxidised metal centers in the EPR product spectra and the disappearance of low-energy features in the UV-vis spectra collectively indicate that the catechol reactions result in the rupture of the bridging peroxo unit. Moreover, analogous to the 4-NO_2_-catechol case, the ESI(+)-MS (Fig. S7†) of the reaction mixtures of LS-4DCHIm with 4-Cl-catechol shows peaks at 1276.56 and 527.36, corresponding to [F_8_Fe^III^(DCHIm)_2_]^+^ and [Cu^I^(DCHIm)_2_]^+^ (*i.e.*, stable reduced form observed instead of [Cu^II^(DCHIm)_4_]^2+^), respectively.

However, in contrast to the reactivity observed with 4-NO_2_-catechol, addition of one equivalent of these catechols evolves negligible amounts of H_2_O_2_ according to the HRP test (2–6% of one equivalent, Table 1). This distinction suggests that subsequent proton transfer(s) followed by electron transfer(s) (PTET) from catechol moieties lead to the reductive cleavage of the O–O core. This may proceed *via* the initial formation of an adduct where the peroxo unit is most likely H-bonded to the catechol (*vide infra*).^45^ Only one mol-equiv. of catechol is required to complete the reactions, thus we propose that one equivalent of *o*-quinone derived from the catechol substrate (Fig. 6A) is also produced, unlike the case of 4-NO_2_-catechol (where the catecholate is generated). The reaction stoichiometry therefore dictates that two protons and two electrons have transferred, and assuming both of the protons and electrons go to the O-atoms of the peroxo moiety, we expect that 2 moles of hydroxide (^−^OH) are formed (Fig. 6A).

Methods aimed to detect the formation of H_2_O/^−^OH have so far been unsuccessful; however, ESI(−)-MS of the reaction mixture {LS-DCHIM + 4-Cl-catechol} shows the formation of *o*-quinone product (Fig. S8†). Additionally, the fact that exactly one equiv. of catechol is required to complete the reaction supports the proposed reaction stoichiometry. Considering the results of the HRP assay and the lack of reactivity with electron-rich catechols having weak O–H BDEs, we hypothesise that although a net two H-atom transfer reaction has occurred, the mechanism most likely incurs a series of catechol-initiated PTET events, including a rate-limiting PT step, rather than two concerted HAT processes. It has been shown in several metal-peroxide systems, including our own work with LS-4DCHIm, that while strong acids promote release of H_2_O_2_*via* M–O cleavage (Scheme 1), relatively weaker acids more favourably achieve reductive O–O cleavage.^45,48^ Comprehensive kinetic analyses for the reactions of the LS-4DCHIm peroxo complex (*vide infra*) provide additional mechanistic insights.

### Mechanistic insights based on kinetic analyses and DFT

**Fig. 7:**
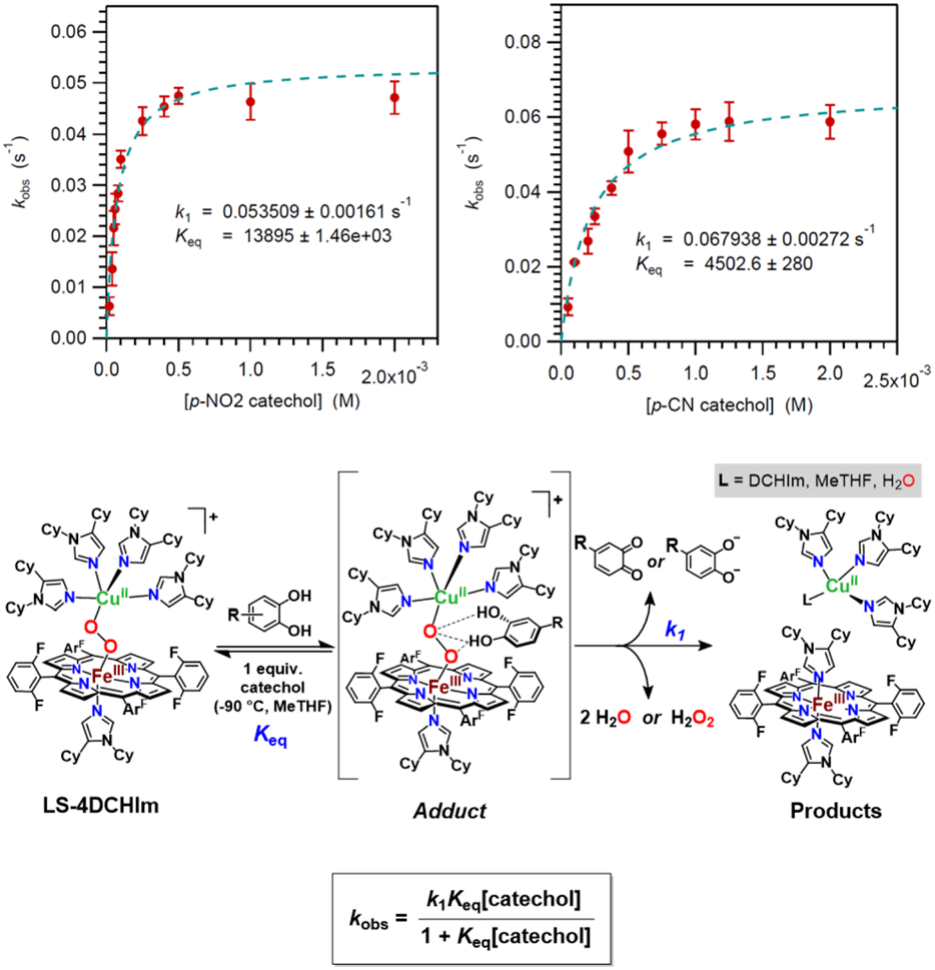
(Top) *k*_obs_*vs.* [catechol] plots for two representative catechols (see Fig. S10† for all other plots) exemplifying the saturation behaviour at high catechol concentrations. (Middle) Scheme showing the kinetic reaction model employed. (Bottom) The equation used to fit the *k*_obs_*vs.* [catechol] data.

To further probe the reaction mechanism(s), pseudo-first order kinetics experiments were employed using increasing concentrations of the catechol substrates described above (see ESI for details and Fig. S10† for all kinetic plots). In all cases, rate saturation occurs at high catechol concentrations (>10 equiv. for all cases except 4-NO_2_-catechol, which reaches a maximum rate with only ∼5 equiv. added) (Fig. 7, top). This type of saturation behaviour is consistent with a mechanism in which rapid equilibrium formation of an intermediate (*K*_eq._) precedes the rate determining step (Fig. 7, middle), and the relationship between the observed rate constant, *k*_obs_, and the physical parameters involved can be described by the equation in Fig. 7, bottom.^45,49–54^

The *K*_eq._ and *k*_1_ values shown in Table 2 were determined from *k*_obs_*vs.* [catechol] plots (Fig. 7, top, and S10†). The fit parameters support the existence of two distinct mechanisms based on the acidity of the catecholic substrate (*vide infra*). Although the rapid reactions prevent isolation or spectroscopic characterization of an intermediate in these cases, we propose that the fast equilibrium step corresponds to the formation of a transient [LS-4DCHIm⋯(catechol)] H-bonded adduct. This proposal is supported by results from our previous studies, including kinetics, rR spectroscopy, and DFT calculations, which demonstrated that LS-4DCHIm can physically accommodate small substrates capable of H-bonding (*e.g.*, 4-NO_2_-phenol).^45^ Additionally, H-bonding to metal-peroxo moieties is known to be “activating” (*i.e.*, it induces weakening of the O–O bond and strengthening of the Fe–O bond, as inferred from rR spectroscopic analysis, thereby reducing the barrier for O–O cleavage).^45,54–57^ In this study, the catechol binding to the LS-4DCHIm complex, likely *via* H-bonding to the peroxo moiety, would effectively initiate (i) a PTET cascade and O–O reductive cleavage (R = –CN, –CF_3_, –Cl_2_, –Cl) or (ii) PT and release of H_2_O_2_ (R = –NO_2_), depending on substrate acidity. Thus, the formation of the proposed H-bonded precursor complex prior to the rate-limiting step of the reaction (*vide infra*) would result in the observed saturation behaviour for the overall reaction kinetics (Fig. 7). Interestingly, the catechol p*K*_a_ values do not correlate linearly with the fitted *K*_eq._ values, which is evidence against a full proton transfer during the initial equilibrium, but rather provides further support for initial formation of an intermediate resembling the H-bonded LS-4DCHIm(catechol) adduct (see Fig. 7).

**Table 2:** Kinetic parameters calculated from fitting *k*_obs_*vs.* [catechol] data plots shown in Fig. 7 (top) with the equation in Fig. 7 (bottom)

Substrate	*K*_eq._ (M^−1^)	*k*_1_ (M^−1^ s^−1^)
4-NO_2_-catechol	13 900 ± 1500	0.0535 ± 0.0016
4-CN-catechol	4500 ± 300	0.0679 ± 0.0027
4-CF_3_-catechol	1900 ± 100	0.0538 ± 0.0017
4,5-Cl_2_-catechol	4000 ± 400	0.0531 ± 0.0011
4-Cl-catechol	2100 ± 200	0.0291 ± 0.0011

To better understand the fundamental relationships between the reaction mechanisms (kinetics) and reactant properties (thermodynamics) and to gain insights into what factors lead to efficient, metal-ion mediated O–O reductive cleavage, it is necessary to evaluate the reactivity outcomes in terms of catechol p*K*_a_, O–H BDE, and ionization potential (IP). For the sake of internal consistency (since these values have not been experimentally measured for the scope of catechols studied herein under the relevant conditions, *i.e.*, solvent and temperature), we report and compare our own DFT-calculated parameters (p*K*_a_, BDE, and IP for the first and second proton, H-atom, and electron, respectively) (see ESI for details†),^58–60^ to gain a sense of relative trends across the scope of catechols employed. The computationally determined parameters are given in Tables 3 and S1.†

**Table 3:** Thermodynamic parameters for the catechols used in this study, calculated using DFT with a THF solvent model^a^

Substrate	O–H BDE 1 (2) kcal mol^−1^	p*K*_a_ 1 (2)	IP 1 kcal mol^−1^
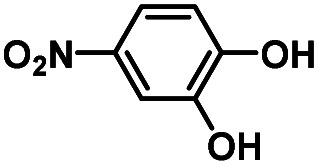	74.2 (69.3)	11.9 (31.4)	151.6
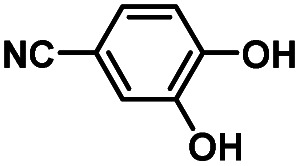	73.6 (68.7)	15.5 (33.5)	147.9
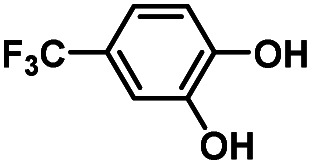	72.3 (66.7)	18.1 (35.0)	146.1
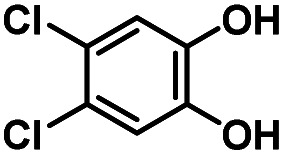	70.5 (65.6)	18.3 (33.5)	142.4
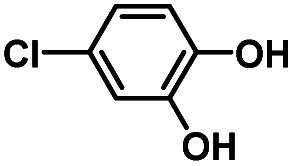	69.7 (65.5)	19.9 (36.3)	140.2
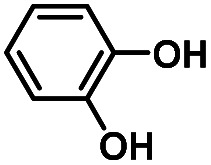	70.4 (65.3)	23.0 (38.5)	138.1
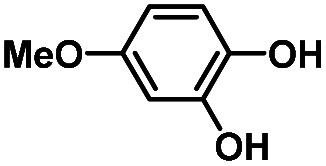	67.8 (64.1)	23.0 (40.8)	127.1
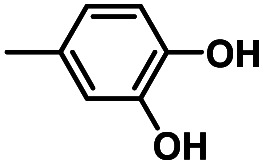	64.1 (62.7)	23.7 (39.2)	133.3

a Shown are the O–H bond dissociation energies (BDE), p*K*_a_'s, and ionization potentials (IP) for the first (and second) H-atom, H^+^, or e^−^, respectively in THF. DFT calculations were performed using the B3LYP(SMD)/6-311++G(2df,p)//B3LYP(SMD)/6-31+G(d) level of theory (see ESI).

**Fig. 8:**
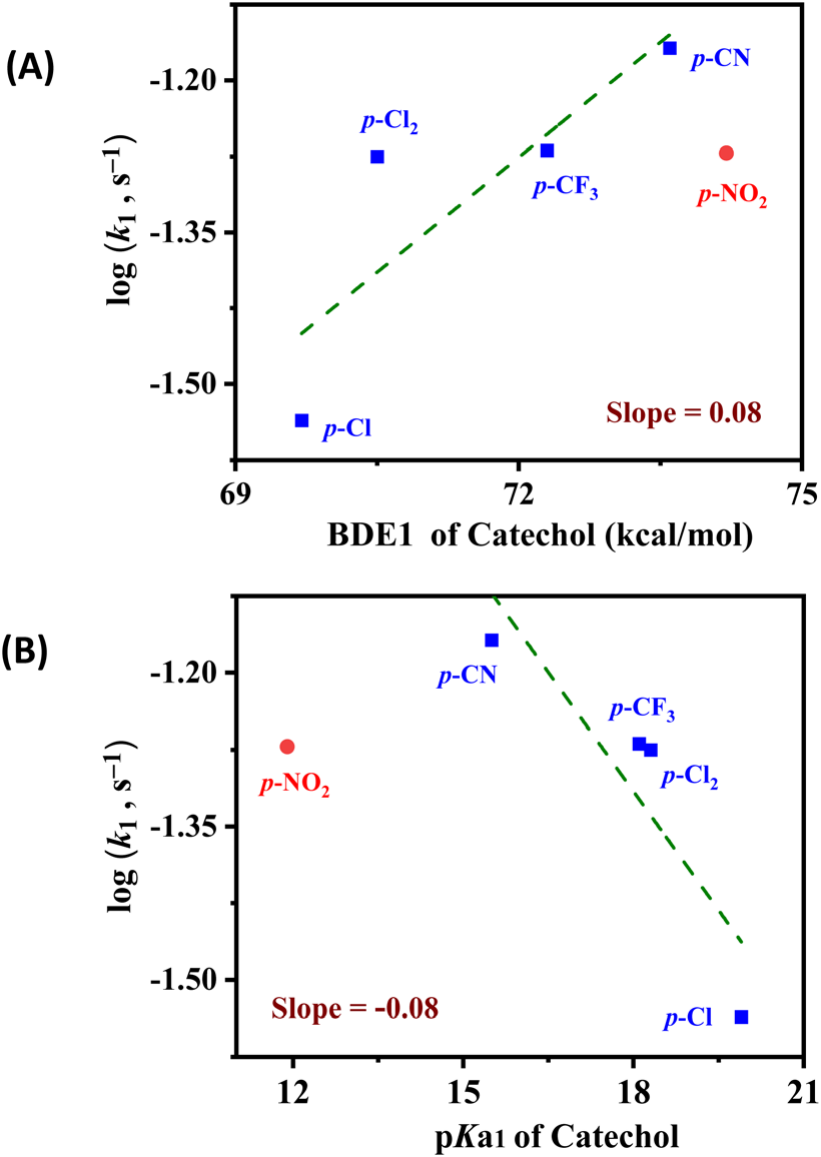
Evans–Polanyi plot (A) and relationship between reaction rate and first p*K*_a_ (B), which show linear trends where the 4-NO_2_-catechol is a clear outlier. The data point for 4-NO_2_ catechol was excluded from the fit, as the reaction mechanism is different, see the discussion in the text. Also, see the ESI.†

Considering the rate-determining step, *k*_1_, in relation to the calculated thermodynamic parameters, O–H BDE and p*K*_a_ for the first H-atom or proton transferred, respectively, a trend is clear. The Evans–Polanyi plot^14,49,61–63^ depicted in Fig. 8A and S11† shows an apparently linear relationship between the *k*_1_ rate constant and the first O–H BDE for the catechols excluding 4-NO_2_-catechol, which corroborates the spectroscopic evidence indicating that 4-NO_2_-catechol follows a different mechanistic pathway from the other four catechols.^25^ The small value of the slope of the linear best-fit trendline in the Evans–Polanyi plot (0.08) denotes a weak dependence of rate on the catechol O–H BDE and an “early” transition state for the reaction.^19,62,64^ This is also consistent with our proposal that the identity of the intermediate is a “reactant-like” H-bonded adduct (Fig. 7, bottom). Furthermore, the positive directionality of the correlation in Fig. 8A reflects the fact that the rate is the slowest for the catechol with the weakest O–H BDE. This is in contrast to the expected relationship between BDE and reaction rate for an HAT reaction, consistent with the observation that electron-rich catechols having very weak O–H BDE do not react with LS-4DCHIm to reductively cleave the bridging peroxide moiety. Since the rates of oxidation of catechols do not well correlate with their bond dissociation energies, we therefore presume that the rate limiting step is a protonation process (Fig. 7 and as discussed in the next paragraph), but not an O–H bond cleavage event which drives the reaction forward.

The relationship between the *k*_1_ rate constant and the p*K*_a_ of the first proton is shown in Fig. 8B, and again, a trend is evident among all the catechols with the exception of 4-NO_2_-catechol. While this is consistent with 4-NO_2_-catechol following a different reaction mechanism, it is clear that the p*K*_a_ is an important parameter for predicting the mechanistic outcomes of this system. Importantly, 4-Cl-catechol, which has the highest p*K*_a_ (is the least acidic), also exhibits the slowest rate of reaction (Table 2). 4-CN-catechol having p*K*_a_(1) = 15.5 defines a threshold where the acidic nature is not strong enough to break the M–O_peroxo_ bond, thus not allowing the production of free H_2_O_2_. Since the 4-CN-catechol is the most acidic of the catechols that yield 2H^+^/2e^−^ O–O reductive cleavage chemistry, and also the fastest reacting, this finding is in excellent agreement with a protonation (or H-bonding)-dependent reaction rate (and mechanism), where proton transfer is rate-determining, and the p*K*_a_ of the catechol substrate is the reaction-determining parameter (2PTET and O–O cleavage: 15.5 ≤ p*K*_a_(1) ≤ 19.9 *vs.* 2PT and H_2_O_2_ release: p*K*_a_(1) ≤ 11.9). As mentioned, previous studies with LS-4DCHIm showed formation of an H-bonded adduct with *p*-NO_2_-phenol (p*K*_a_ (THF, expt.) = 18);^65^ however, in that case, an external electron source was required for an overall PCET reaction to occur.^45^

**Fig. 9:**
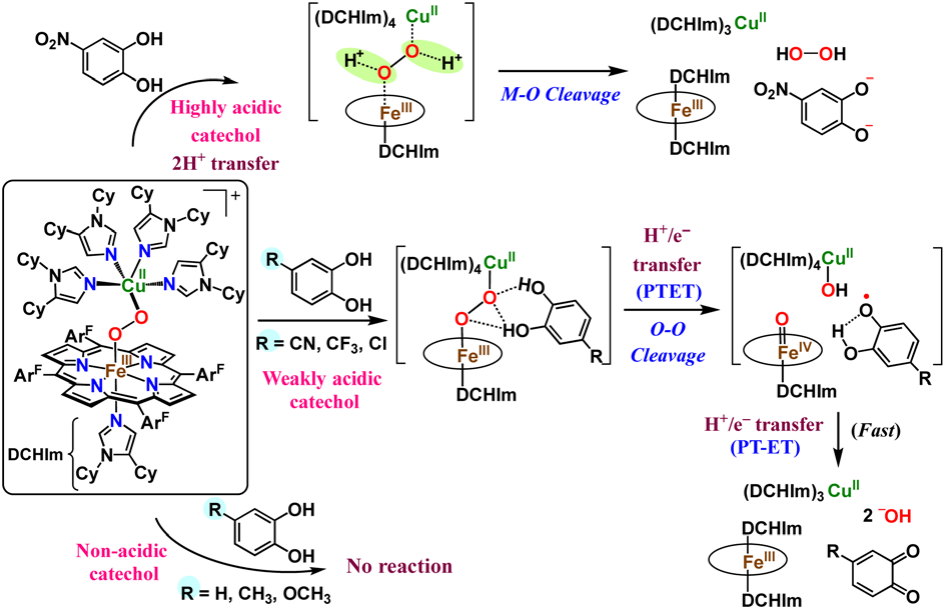
Mechanistic pathways for reactions of LS-4DCHIm with catechols of varying p*K*_a_'s.

In the reactions of weakly acidic catechols (which also have a sufficiently low O–H BDE), the catecholic substrate is presumed to provide the protons and, more importantly, the electrons necessary for reductive O–O bond cleavage to produce H_2_O. Thus, based on H_2_O_2_ quantification and kinetic analysis (supporting a rate limiting proton transfer process), we propose that the 4-NO_2_-catechol follows a fast 2PT mechanism, and the 4-substituted –CN, –CF_3_, –Cl_2_, and –Cl catechols follow a 2PTET mechanism. In the cases involving 2H^+^/2e^−^ reactivity (net 2H˙ transfer from the catechol to the peroxo complex, giving the respective *o*-quinone) and resultant O–O reductive cleavage, the catechol must be acidic enough to initiate the reaction, likely by activating the peroxo moiety *via* H-bonding,^45,54^ while also having sufficiently weak BDE(s). In the grand scheme of O_2_-activation and reduction (Fig. 9), these findings indicate that, for successful O_2_-reductive cleavage to occur within a bridging heme–peroxo–Cu construct, a PTET mechanism is preferred over an HAT mechanism, although the same quantity of protons and electrons are transferred in either case. This is consistent with previous findings that H-bonding and/or protonation of a metal-bridging peroxo moiety can activate the O–O bond and lower the barrier to reduction;^45,54^ both of these interactions are dependent on the H-bonding ability or p*K*_a_ of the substrate.

It is important to note that, although parameters have been determined for both the first and second proton and/or electron, it is reasonable to assume that the first p*K*_a_, BDE, or IP determines the reaction pathway (Fig. 9), especially because the trends are consistent between first *vs.* second H^+^, H˙, and e^−^ (Table 3). Because O–O cleavage of a formal peroxo moiety only requires one electron (plus proton(s)), one might predict that catechols with weaker O–H BDEs or lower IPs will react most favourably to afford O–O cleavage products. If, in fact, only one electron is transferred (with or without proton(s)), the resultant products are expected to be a high-valent Fe^IV^=O and semiquinone; however, these are unlikely to persist or be observable, even under the cryogenic reaction conditions due to the highly reactive nature of the compound II-type species^45^ and the fact that the second O–H bond in the semiquinone is significantly weaker than the first (see Fig. 9 (center), 2, and Table 3).^14,60,66^ This is consistent with the proposed equilibrium formation of an H-bonded intermediate, which activates the O–O bond for cleavage; in this case, it also primes the proximal *o*-hydroxy group of the semiquinone to transfer its proton (and electron) in a rapid cascade.^67^ It is important to note that in HCO enzymes and in some model studies,^54^ the fourth and final electron required for complete dioxygen reduction has been shown to come from the iron center, thereby generating the ferryl species, Fe^IV^=O (single electron transfers from the Fe, Cu, and Tyr moieties provide the first three reducing equivalents). In the catechol reactions, which we propose result in O–O reductive cleavage, the rapid reaction kinetics prevent observation of the intermediate heme species. Therefore, we cannot determine whether both of the catecholic electrons go to the peroxo moiety (bypassing formation of a high-valent iron-oxo species) or, rather, if the two electrons required for peroxo-cleavage originate from the catechol (1e^−^; catechol to semiquinone) and the heme-iron center (1e^−^; Fe^III^ to Fe^IV^). In the latter case (depicted in Fig. 9), we presume that the second electron from the catechol would rapidly reduce the Fe^IV^ intermediate to the more stable Fe^III^ product observed spectroscopically, resulting in the release of hydroxide. Furthermore, no reaction is observed with the less acidic R-catechols (R = H, Me, OMe), as indicated by the absence of changes in the UV-vis spectra in the Soret and Q-band regions (given in Fig. S12, ESI†), and also leads to the possibility of H-bonded adduct formation ambiguous.^68^

## Conclusions

In this report, we have described the reactions of a low-spin heme–peroxo–Cu complex with a series of substituted catechols at low temperature (−90 °C) in MeTHF, which interestingly span two different mechanisms based on the thermodynamic parameters of the catecholic substrates, namely their O–H BDE and p*K*_a_.

Product determination, H_2_O_2_ quantification, and kinetic evaluations of the reactions of LS-4DCHIm with weakly to highly acidic catechols in combination with DFT calculations have led us to propose that an initial p*K*_a_-dependent H-bond adduct formation is critical for activating the peroxide bridge and priming the substrate for the subsequent proton and electron transfers (Fig. 9, center pathway). Indeed, such H-bonded precursor complexes have been proposed to reduce the activation barrier of the overall reaction, making the proton/electron transfer energetically more favourable.^32^ However, a balance of p*K*_a_ and O–H BDE is necessary to avoid double PT and release of H_2_O_2_, rather than oxidation of the catechol and effective O–O cleavage. Using a series of catechols with a range of p*K*_a_'s and O–H BDEs, we have shown that the NO_2_-catechol participates in 2PTs and H_2_O_2_ release (Fig. 9, top pathway), whereas reactions of LS-4DCHIm with CN–, CF_3_–, Cl_2_–, and Cl-catechols release negligible H_2_O_2_ and lead to O–O reductive cleavage *via* a PTET mechanism.

Interestingly, the LS-4DCHIm peroxo complex is unreactive toward catechols with electron-donating substitution (*i.e.*, non-acidic, with weak O–H bonds, such as those in 4-OCH_3_-catechol and 4-CH_3_-catechol) or unsubstituted catechol, even if added in excess (up to 10 equiv.). This result has mechanistic implications, suggesting that even if catechols are competent in providing two protons and two electrons (*i.e.*, two net H-atoms) to effect O–O bond reductive cleavage, the mechanism of transfer is stepwise. In other words, catechols are unlikely to transfer protons and electrons in a concerted manner, and therefore, weak substrate O–H BDEs alone are not sufficient to activate and reduce the heme/Cu-bridged peroxide moiety (Fig. 9, bottom pathway). This study highlights the key balance of H^+^ and e^−^ transfer to govern the O–O cleavage *versus* M–O bond rupture and ROS release, which is relevant to understanding biochemical O_2_-reduction during cellular respiration and for the rational design of practical catalysts and fuel cell technologies. Additionally, this work could contribute to a broader understanding of enzymatic redox processes and provide further insights into the role of PCET in biological systems.

## Data availability

The data supporting this article have been included as part of the ESI.†

## Author contributions

K. D. K., S. M. A. and G. B. W. conceived the project idea and K. D. K. supervised the investigation; S. M. A., S. P., and G. B. W. synthesized all compounds, and generated and analyzed the experimental data with the help of other co-authors; S. P. performed the theoretical calculations and S. P., S. M. A., and G. B. W. interpreted the data. J. H. and B. S. P. carried out the EPR quantitation. K. D. K., S. M. A., S. P., and G. B. W. wrote and edited the manuscript with input from all other co-authors. All authors have approved the final version of the manuscript.

## Conflicts of interest

There are no conflicts to declare.

## Supplementary Material

SC-016-D4SC05623J-s001
