# Supplementary material for: Reactivity of a heterobinuclear heme–peroxo–Cu complex with para-substituted catechols shows a pKa-dependent change in mechanism
Source: Chem Sci. 2024 Dec 30;16(5):2402–12. doi: 10.1039/d4sc05623j (PMC11707526; doi:10.1039/d4sc05623j)
Supplement: SC-016-D4SC05623J-s001 [file SC-016-D4SC05623J-s001.pdf]

Supplementary Information (SI) for Chemical Science.
This journal is © The Royal Society of Chemistry 2024

SUPPLEMENTARY INFORMATION for

Reactivity of a heterobinuclear heme-peroxo-Cu complex with *p*ara-substituted catechols shows a p*K*a-dependent change in mechanism

Sanjib Panda,[#,a] Suzanne M. Adam,[#,a] Hai Phan,[a] Patrick J. Rogler,[a] Pradip Kumar Hota,[a] Josh Helms,[b] Brad S. Pierce,[b] Gayan B. Wijeratne,*[,b] and Kenneth D. Karlin*[,a]

[a]Department of Chemistry, Johns Hopkins University, Baltimore, Maryland 21218, United States

[b]Department of Chemistry & Biochemistry, The University of Alabama, Tuscaloosa, Alabama 35487, United States

Table of Contents: **Page**

1. Materials and Methods and DFT calculations S3-S7
2. Fig. S1: EPR spectrum of {LS-4DCHIm + NO_2-catechol} S8
3. Fig. S2: UV-vis spectrum of {LS-4DCHIm + NO_2-catechol} S9
4. Fig. S3: ESI(+)-MS of {LS-4DCHIm + NO_2-catechol} S10-S11
5. Fig. S4: HRP analysis for H_2O_2 detection S12
6. Fig. S5: NaI test for H_2O_2 detection S13
7. Fig. S6: EPR spectra of {LS-4DCHIm + R-catechol} (R = CN, CF_3, Cl_2, Cl) S14-S15
8. Fig. S7: ESI(+)-MS of {LS-4DCHIm + Cl-catechol} S16
9. Fig. S8: ESI(-)-MS of {LS-4DCHIm + Cl-catechol} S17-S18
10. Fig. S9: UV-vis spectra of {LS-4DCHIm + R-catechol} (R = CN, CF_3, Cl_2, Cl) S19
11. Fig. S10: Kinetics of {LS-4DCHIm + R-catechol} (R = CN, CF_3, Cl_2, Cl) S20
12. Fig. S11: Evans-Polanyi plot S21
13. Fig. S12: UV-vis spectra of {LS-4DCHIm + R-catechol} (R = H, Me, OMe) S22
14. Table S1: Thermodynamic parameters of catechols S23
15. Cartesian coordinates S24-S72
16. References S73-S74

Materials and methods

1. General information

Unless stated otherwise, all chemicals and solvents were of commercially available quality and used without further purification. Inhibitor-free 2-methyltetrahydrofuran (MeTHF) solvent was distilled over sodium/benzophenone under an argon atmosphere. Both solvents were subsequently degassed by bubbling argon through them before use. Freshly distilled and Ar sparged MeTHF was stored in the glovebox over 4 Å activated molecular sieves for at least 2 days prior to use. $[Cu^{I}(CH_3CN)_4]BArF$ (BArF stands for $B(C_6F_5)_4$), and F_8Fe^{II} were synthesized as previously published literature procedures,[1,2] and their purities were verified by elemental analysis and/or ^{1}H NMR. The preparation and handling of air-sensitive compounds were performed under a mBRAUN LABmaster Pro-130 glovebox (<1 ppm of O_2 and <1 ppm of H_2O) filled with nitrogen. Dioxygen gas was purchased from Airgas company and dried through Drierite.

UV-vis and kinetics: All UV-Vis measurements were carried out using a Hewlett Packard 8453 diode array spectrophotometer with a quartz Schlenk cuvette cell. The spectrometer was equipped with HP Chemstation software and a Unisoku thermostated cell holder for low temperature experiments. The complex, **LS-4DCHIm,** was generated as previously described at 0.1 mM concentration at low temperature (–90 °C unless otherwise stated) in a 1 cm path length, rubber septum-capped, quartz Schlenk cuvette.[3] To obtain conditions optimal for observing Soret peaks, the same concentration (0.1 mM) was used in a 2 mm path length Schlenk cuvette. For pseudo-first order kinetic experiments, following generation of the heme-peroxo-Cu complex, 20-100 μL of a solution containing the desired number of equivalents of catechol was added via gastight syringe and mixed by bubbling Ar. Spectra were recorded every 3.0 seconds, and the observed rate constant, k_{obs} (sec^{-1}), corresponds to the initial rate measured as the ΔAbs(845-700 nm) over time adjusted for concentration and molar absorptivity ($\varepsilon = 420\ M^{-1}cm^{-1}$). The k_{obs} rate vs. catechol concentration plots were fit with the program, Igor Pro 4.00, to obtain K_{eq} and k_1 parameters using the equation shown in Fig. 6 which fits with the mechanistic model.

Electron paramagnetic resonance (EPR): A 0.6 mL solution of the **LS-4DCHIm** complex was generated anaerobically as previously described[3] at 2.0 mM concentrations at –90 °C (acetone/liq. N_2 bath) in a 5 mm, rubber septum-capped, EPR tube. The desired

o-catechol was added via gastight syringe, mixed by bubbling Ar, and frozen prior to recording spectra. Spectra were taken with an ER 073 magnet equipped with a Bruker ER041 X-Band Microwave bridge and a Bruker EMX 081 power supply. Microwave frequency = 9.41 GHz, microwave power = 0.201 mW, attenuation = 30db, modulation amplitude = 10 G, modulation frequency = 100 kHz, temperature = 10K. Due to significant spectral overlap between the Cu(II) and low-spin Fe(III) heme signals, quantification of these species was carried out by spectral addition based on a calibration curve of the authentically generated $Cu^{II}(DCHIm)_4$ and $F_8Fe^{III}(DCHIm)_2$ species.[4] The copper signal for the reaction products of the CN-, CF_3-, Cl_2- and Cl-catechol reactions at low temperature is comprised of a combination of $Cu(II)(DCHIm)_4$ and another Cu(II) species, where we propose the organic product of catechol oxidation is binding to the Cu(II) center due to the similar shape observed in frozen EPR samples of Cu(II) complexes of β-diketones such as acetylacetonate (acac),[5] however, the spectrum of $Cu^{II}(DCHIm)_4$ is unchanged by addition of authentic *o*-quinone (or catecholate), even at low temperature.

Continuous-wave (CW) EPR spectroscopy for spin quantification was performed at 20 K using a Bruker ELEXSYS E540 X-band spectrometer (Bruker-Biospin, Billerica, MA). Cryogenic EPR data was collected using a ColdEdge Stinger closed-loop liquid helium cryosystem inserted into an Oxford ESR900 cryostat. A LakeShore 336 temperature controller was used to regulate sample temperature. The {LS-4DCHIm + Cl-catechol} reaction mixture and the standards were recorded under non-saturating conditions. Instrumental parameters: microwave frequency, 9.62 GHz; microwave power, 20 mW; modulation amplitude, 0.9 mT. Simulations of EPR spectrum was completed using SpinCount (ver. 8.0.9019.20391) developed by Professor Michael Hendrich at Carnegie Mellon University by utilizing the general spin Hamiltonian seen in **Eq. 1**.[6,7]

$$\hat{H} = \beta_e \vec{B}_0 \cdot \tilde{\boldsymbol{g}} \cdot \hat{S} + \hat{S} \cdot \tilde{\boldsymbol{A}} \cdot \hat{I} \qquad \textbf{(Eq. 1)}$$

Here, $\tilde{\boldsymbol{g}}$ is the *g*-tensor and $\tilde{\boldsymbol{A}}$ is the nuclear hyperfine interaction, which is treated with second-order perturbation theory.[8,9] Simulations were generated with consideration of all intensity factors, both theoretical and experimental, to allow for determination of species concentration. The only unknown factor relating the spin concentration to signal intensity was an instrumental factor that is specific to the microwave detection system. This factor was determined by a 1.0 mM Cu(EDTA) spin standard prepared from a copper

atomic absorption standard solution purchased from Sigma-Aldrich. All samples were prepared in custom made 4 mm Quartz EPR tubes made at the UA glassblowing facility. For all analytical EPR measurements, data was collected under non-saturating conditions. **Electrospray ionization mass spectrometry (ESI-MS):** ESI-MS data were acquired using a JEOL AccuTOF LC-Express spectrometer with Mass Mountaineer™ version 6 software. Samples were prepared as 0.1 mM solutions in 2-methyltetrahydrofuran (MeTHF) and analyzed under an argon atmosphere. For positive-mode ESI-MS, the needle voltage, detector voltage, and ion guide voltage were set to 2000 V, 2200 V, and 2100 V, respectively. In negative-mode ESI-MS, the needle voltage, detector voltage, and ion guide voltage were adjusted to -2000 V, 2200 V, and 1000 V, respectively.

H_2O_2 quantification by horseradish peroxidase (HRP) test: The spectrophotometric quantification of hydrogen peroxide was achieved by recording the intensity of the diammonium 2,2′-azino-bis(3-ethylbenzothiazoline-6-sulfonate (AzBTS-$(NH_4)_2$) peaks (monitored at 418 nm to minimise error) oxidised by horseradish peroxidase (HRP) which was adapted from published procedures.[4,10] In a typical experiment, 3 mL of the **LS-4DCHIm** complex [0.1 mM] was generated at −90 °C (as above for UV-Vis experiments). If required, solutions containing the desired catechol [0.1 mM] was added and the reaction was allowed to proceed until completion (i.e. no further spectral changes were observed). The reaction crude was subjected to the H_2O_2 analysis as described below, *both before and after* quenching by addition of a 100 μL solution containing 2.5 equiv. of the strong acid, [DMF·H^+]($CF_3SO_3^-$) [0.25 mM]. Hydrogen peroxide (H_2O_2) was detected using the procedure described using the following stock solutions: 300 mM sodium phosphate buffer, pH 7.0 (solution A), 1 mg/mL AzBTS-$(NH_4)_2$ (solution B), 4 mg of HRP (type II salt free (Sigma)), and 6.5 mg of sodium azide in 50 mL of water (solution C) all maintained at 0 °C. Quantification of hydrogen peroxide was achieved by adding a 100 μL of the −90 °C MeTHF sample solution to a cuvette containing 1.3 mL of water, 500 μL of solution A, 100 μL of solution B, and 50 μL of solution C (all chilled). After mixing for 15 s, the samples were allowed to incubate for ~2 min until full formation of the 418 nm band was achieved, allowing for quantification of the H_2O_2 in solution (Table 1).

Synthesis of 4-NO_2-catecholate·$(Na)_2$. In a glovebox under N_2 atmosphere, 50 mg NO_2-catechol (0.323 mmol) was dissolved and stirred with a stir bar in 1 mL 2-MeTHF. A

5 mL suspension containing 15.4 mg (0.646 mmol) NaH in 2-MeTHF (minimally soluble) was added and the mixture was allowed to stir for 1 hour under inert atmosphere, during which time, the evolution of H_2 gas bubbles was observed. Solvent was removed completely from the product reaction mixture and the crude solid was dissolved in dry acetone, filtered to remove excess NaH, and redried to obtain an orange solid catecholate. It was characterised by UV-vis (λ_{max} = 275 nm (ε = 29,000 $M^{-1}cm^{-1}$), 440 nm (ε = 27,000 $M^{-1}cm^{-1}$); see **Fig. S2**), and ^{1}H-NMR (Acetone-d_6; δ = 7.62 (1H), 7.41 (1H), 6.30 (1H) ppm, vs. 4NO_2-catechol δ = 7.73 (2H), 7.02 (1H), 9.04 (broad, 2H) ppm).

DFT calculations: Values for IP, p*K*a and BDE were performed according to a previously described method[11] using the Gaussian 16 software package.[12] The geometry of each catechol was fully optimised at the B3LYP/6-31+G(d) level of theory without further constraints. The solvation energy was calculated using the SMD-PCM solvation model for the THF solvent. For each catechol, the singly protonated form was found to be in its lowest energy state when the proton was between the oxygens. After optimisation, single point energy calculations were performed at the B3LYP/6-311++G(2df,p) level of theory.

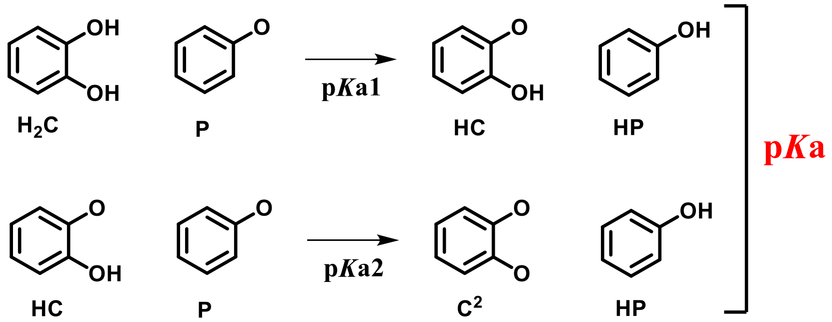

Scheme S1 Isodesmic reaction scheme used for computational determination of p*K*a values (H_2C, C^{2-}, HP, P^- stands for catechol, catecholate, phenol, and phenolate, respectively).

Since DFT tends to systematically over or underestimate chemical properties, and difficulties exist in accurately calculating the solvation energy of protons, an isodesmic reaction using a simple phenol was used to improve the calculation accuracy (Scheme S1).[11] An example calculation (Gibbs free energy change for each reaction) is given in Eq. 2 for the determination of p*K*a1, where H_2C and HP represent the catechol and phenol, respectively and their monodeprotonated forms are designated as HC^- and P^-.

$$\Delta G(pKa1) = \big(G(HC^-) + G(HP)\big) - \big(G(H_2C) + G(P^-)\big) \qquad \textbf{(Eq. 2)}$$

The Gibbs free energy for the deprotonation was converted to p*K*a using Eq. 3, where F is faradays constant.

$$pKa1(H_2C) = pKa(HP) + \Delta G(pKa1)/2.303\mathrm{RT} \qquad \textbf{(Eq. 3)}$$

The experimental p*K*a of HP (i.e., PhOH) in THF is 21.0.[13]

On the other hand, the BDEs for the catechols were calculated by obtaining its free energy difference with respect to a "single box" calculation of the semiquinone ($HC^{\bullet}$)/quinone (Q) and an H-atom at 10 Å apart (Scheme S2, Eqs. 4-5). The IP values are calculated based on Eqs. 6-7 (Scheme S2).

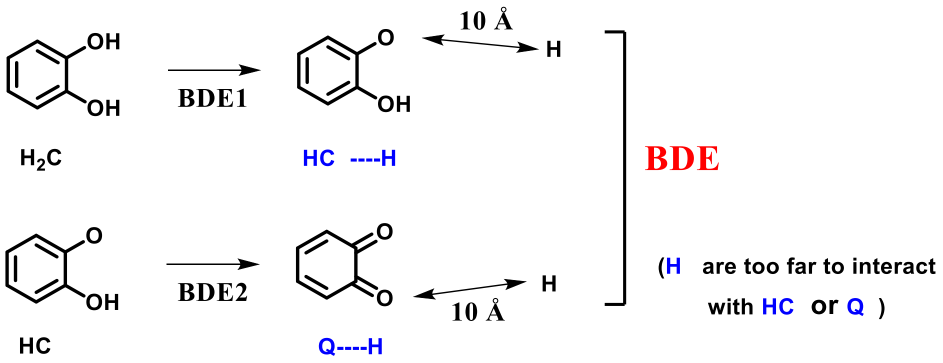

Scheme S2 Computational determination of thermodynamic parameters (BDE, IP) of catechols (H_2C and Q stands for catechol and quinone, respectively).

$$\Delta G(BDE1) = \{G(\mathrm{CH}^{\bullet} \quad \mathrm{H}^{\bullet}) - G(\mathrm{H_2C})\} \qquad \textbf{(Eq. 4)}$$

$$\Delta G(BDE2) = \{G(\mathrm{Q} \quad \mathrm{H}^{\bullet}) - G(\mathrm{CH}^{\bullet})\} \qquad \textbf{(Eq. 5)}$$

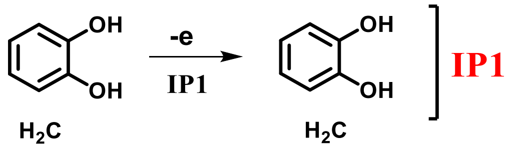

$$\Delta G(IP1) = \{G(\mathrm{CH_2}^{\bullet +}) - G(\mathrm{H_2C})\} \qquad \textbf{(Eq. 6)}$$

$$\mathrm{IP1} = \{\Delta G(IP1) / \mathrm{F}\} \qquad \textbf{(Eq. 7)}$$

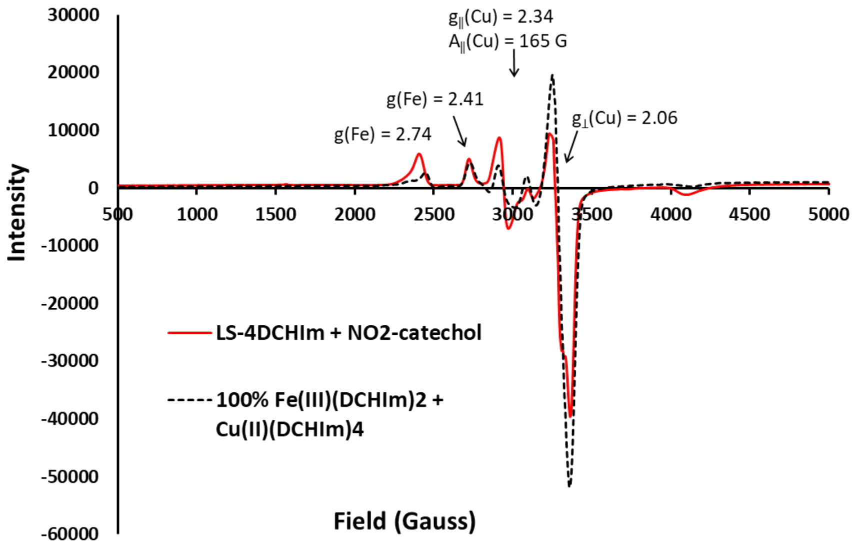

Fig. S1 EPR spectra of the product mixture following the reaction of **LS-4DCHIm** with NO_2-catechol (red) and the theoretical yield (spectral addition of 1 mM $F8Fe^{III}(DCHIm)_2$ + 1 mM $Cu^{II}(DCHIm)_4$) shown as a dotted black line. Fitting by spectral addition gives a semi-quantitation of the yield for the Fe and Cu species of 74%.

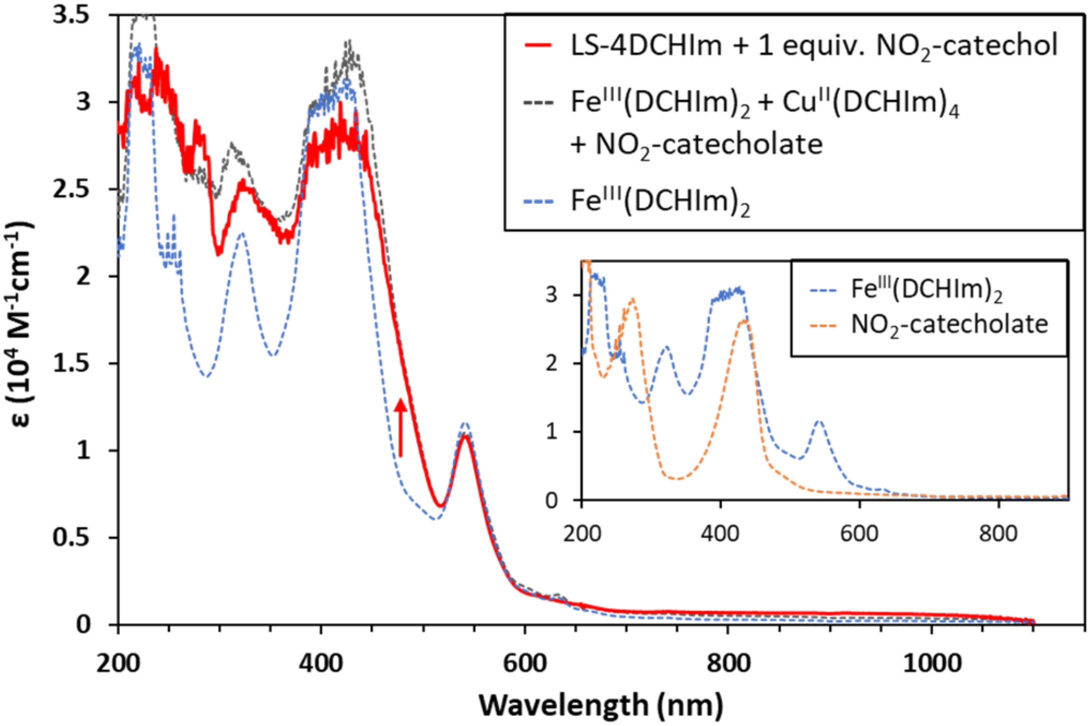

Fig. S2 UV-vis spectrum of the {**LS-4DCHIm** + NO_2-catechol} reaction products (red) compared with a spectrum of a 1:1:1 mixture containing authentic $Fe^{III}(DCHIm)_2$, $Cu^{II}(DCHIm)_4$, and NO_2-catecholate (grey, dotted line) showing nearly complete overlap, and the $Fe^{III}(DCHIm)_2$ spectrum without the catecholate in solution is shown as a blue dotted spectrum for comparison (the increase in absorbance due to the NO_2-catecholate is indicated with a red arrow). The inset shows the individual spectra for $Fe^{III}(DCHIm)_2$ (blue, dotted) and NO_2-catecholate (orange, dotted).

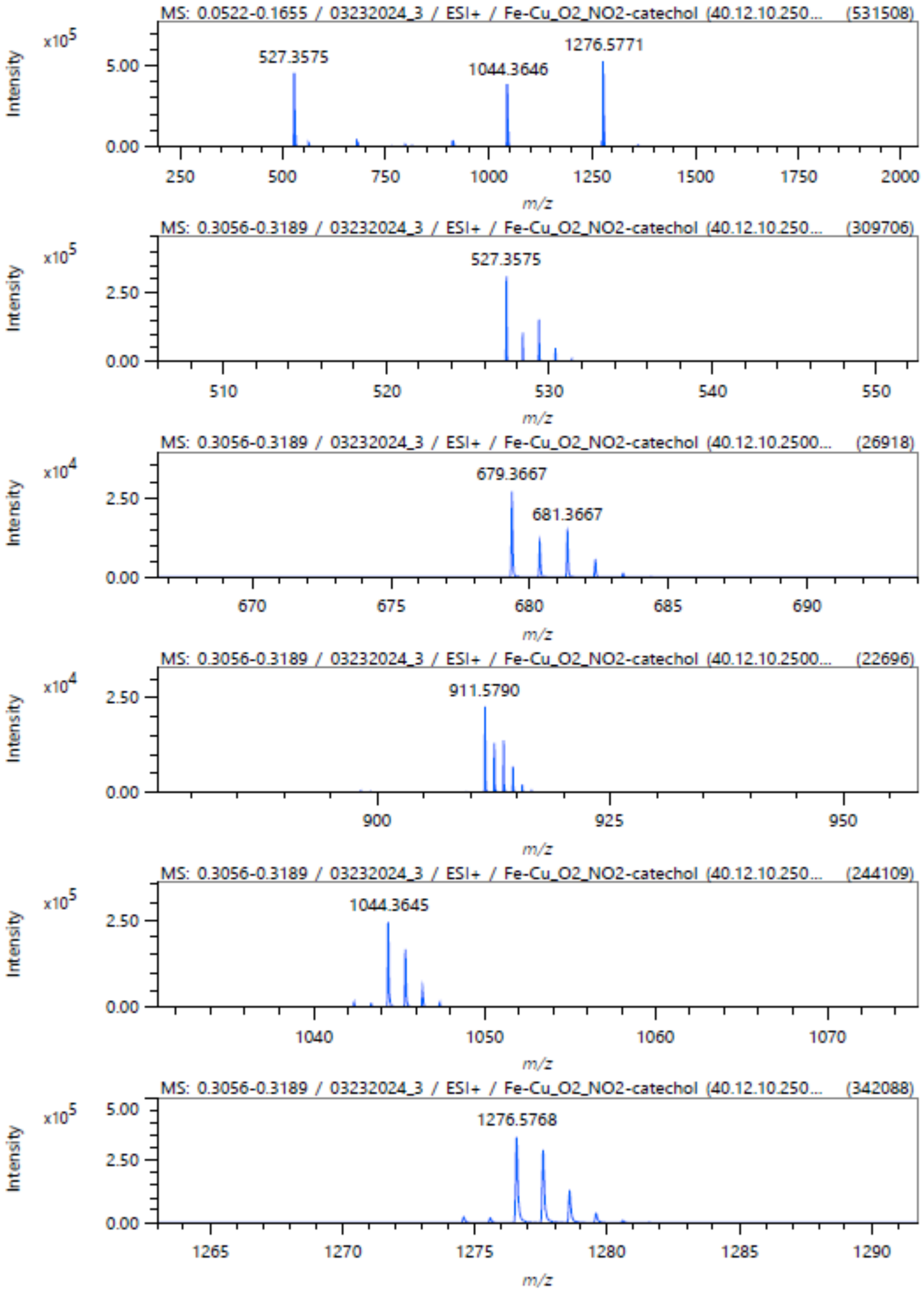

Fig. S3 Experimental ESI(+) mass spectra of **LS-4DCHIm** with 4-NO_2-catechol. Masses at 527.35 ([Cu(DCHIm)$_2$]$^+$), 679.37 ([Cu(DCHIm)$_2$(4-NO_2-catecholate)]$^+$, detected as [M-H]$^+$), 1044.36 ([F_8Fe(DCHIm)]$^+$), 1276.58 ([F_8Fe(DCHIm)$_2$]$^+$).

The 1-unit mass discrepancy for $[(DCHIm)_2Cu^{II}(4\text{-}NO_2\text{-catecholate})]^+$ (679.37 instead of 680.38) in the ESI(+)-MS (**Fig. S3**) may be attributed to $\{M\text{-}H\}^+$. This observation was consistently noted in our repeated experiments. We suggest that a deprotonation may occur from the *N*-substituted cyclohaxane ring of DCHIm. Due to aromaticity, that *N*-atom should be electron poor, making the cyclohexane ring and C–H bond adjacent to the imidazole N-atom partially acidic, which might favor deprotonation in some occasional cases, e.g., in the presence of catecholate. There is literature precedent for such an $\{M\text{-}H\}^+$ peak, where it is stated that this type of mass spectrometric deprotonation is "usually limited to compounds possessing acidic protons or compounds that could generate such ones by tautomerism".[14]

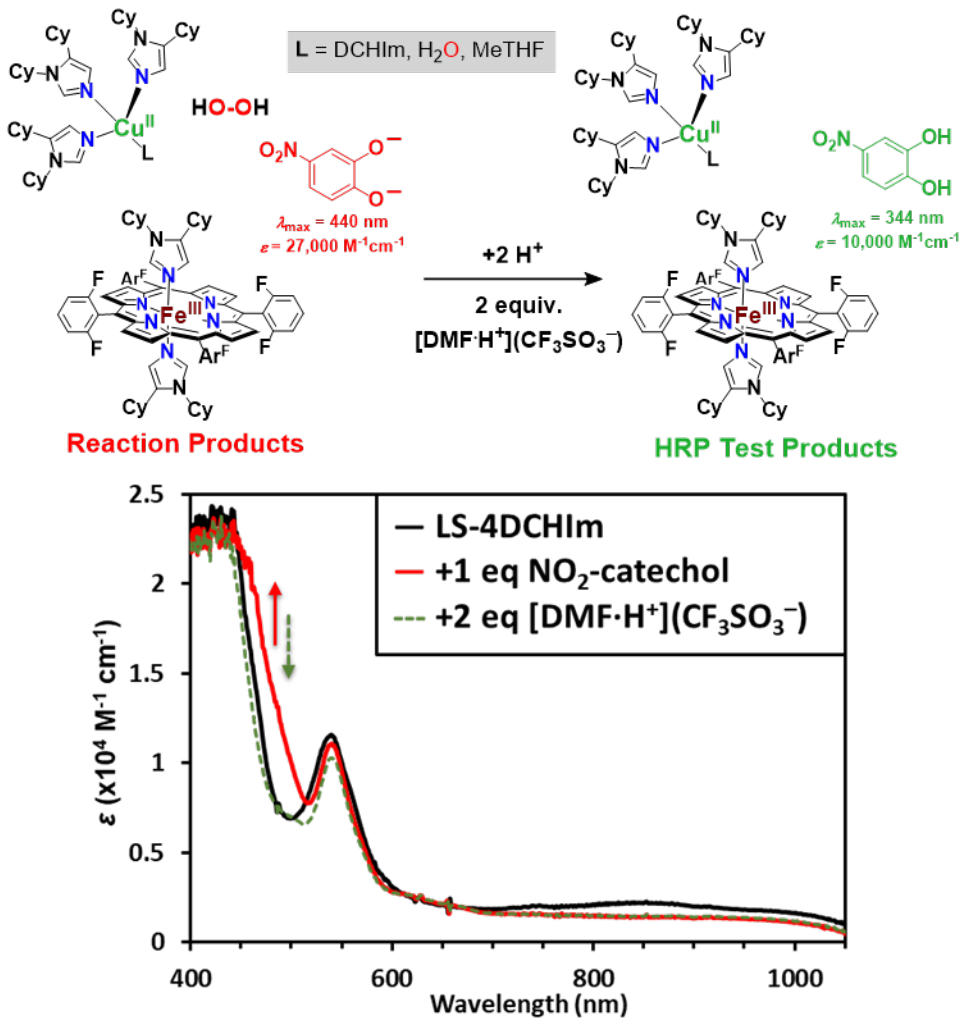

Fig. S4 Scheme and UV-vis spectra of the HRP analysis of the {**LS-4DCHIm** + NO_2-catechol} reaction. Addition of 1 equiv. NO_2-catechol to LS-4DCHIm (black spectrum), yields the reaction products shown in the top scheme (left) corresponding to the red spectrum where the NO_2-catecholate absorbance is evident as a strong shoulder on the Soret band. Addition of 2 equiv. strong acid for HRP analysis results in a decrease of this absorbance (with no other spectral changes), consistent with protonation of the catecholate back to the catechol as shown in the scheme, right.

Sodium Iodide Test for H_2O_2 detection. In addition to the HRP test, the H_2O_2 generation in the reaction mixture was also tested using the iodide test. From a 0.1 mM aliquot of the {LS-DCHIm + NO_2-catechol} solution, 150 µL was transferred to a separate cuvette containing 2 mL of saturated NaI solution in MeCN at room temperature. After incubating for 5 minutes, the UV-vis spectrum of the final mixture was recorded, showing the formation of triiodide (I_3^-), with a primary absorption at 362 nm (**Fig. S5**). While the NO_2-catechol tested positive for H_2O_2, the Cl-catechol remained unreactive, indicating no H_2O_2 formation. Representative overlapping spectra for NO_2-catechol and Cl-catechol are provided here.

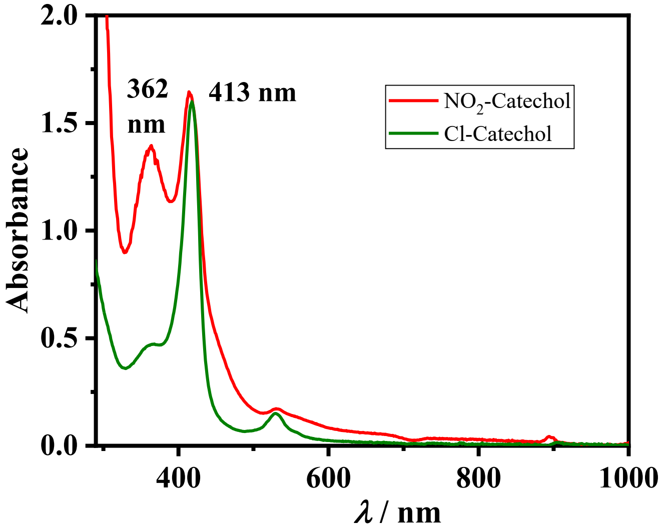

Fig. S5 UV-vis spectra of I_3^- (λ_{max} = 362 nm) generated from {**LS-4DCHIm** + 4-NO_2-Catechol} (Red) and {**LS-4DCHIm** + 4-Cl-Catechol} (green) product with NaI solution in CH_3CN.

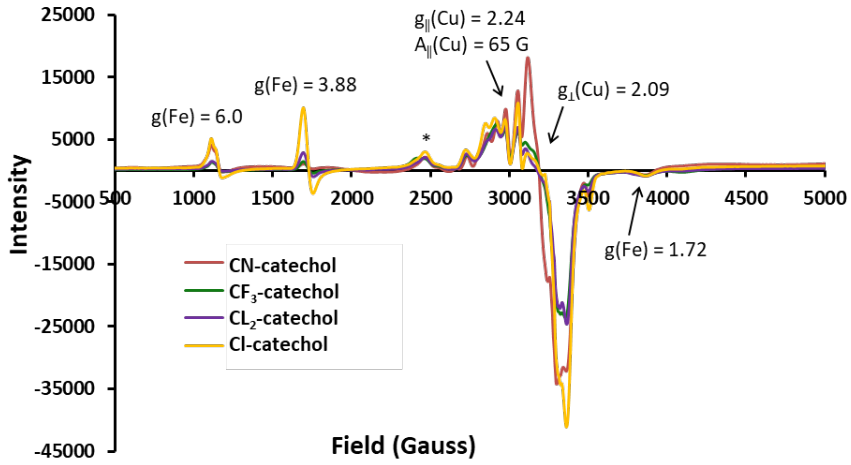

Fig. S6a EPR spectra of the product mixtures following addition of CN-, CF_3-, Cl_2- and Cl-catechols to a solution of **LS-4DCHIm** at –90 °C. Quantification of these species is complicated, however, the labeled (Fe) *g*-values represent the HS species impurity (< 10% in each case), and the peak marked with an asterisk is uniquely due to the LS $Fe^{III}(DCHIm)_2$ product, which can be generated authentically. The LS-heme product quantified based on the intensity of this peak and comparison to a calibration curve generated for the authentic $Fe^{III}(DCHIm)_2$ species provides semi-quantitation of LS-heme product yields in these cases as: (CN (89%), CF_3 (88%), Cl_2 (83%) and Cl (90%)).[4]

The copper signal for the reaction products of the CN-, CF_3-, Cl_2- and Cl-catechol reactions at low temperature is comprised of $Cu^{II}(DCHIm)_4$ and another Cu(II) species, where we propose the organic product of catechol oxidation is interacting with the Cu(II) center due to the similar shape and small hyperfine splitting observed in frozen EPR samples of Cu(II) complexes of β-diketones such as acetylacetonate (acac).[5] Analysis of this product mixture is complicated by the 5 equiv. of coordinatively-labile DCHIm species in solution, which bind strongly to iron (high yields of $Fe^{III}(DCHIm)_2$), potentially leaving the coordinatively unsaturated $Cu^{II}(DCHIm)_3$ species, which we propose may interact with the quinone product. However, the spectrum of $Cu^{II}(DCHIm)_4$ (isolated solid) is unchanged by addition of authentic *o*-quinone (or catecholate), even at low temperature.

To address these ambiguities, we have carried out quantification of the total final Cu(II) concentration via (i) double integration, and (ii) simulation of the final EPR features resulting from the reaction mixture {LS-4DCHIm + Cl-catechol}. In that, a 0.4 mL solution

of 1 mM LS-4DCHIm was prepared in MeTHF at -90 °C (using an acetone/liquid N_2 bath) in a 4 mm rubber septum-capped EPR tube. To this, 1 equivalent of Cl-catechol (10 µL of a 40 mM solution) was added via gastight syringe, mixed under Ar, allowed to react for 30 minutes, and then frozen prior to spectral acquisition.

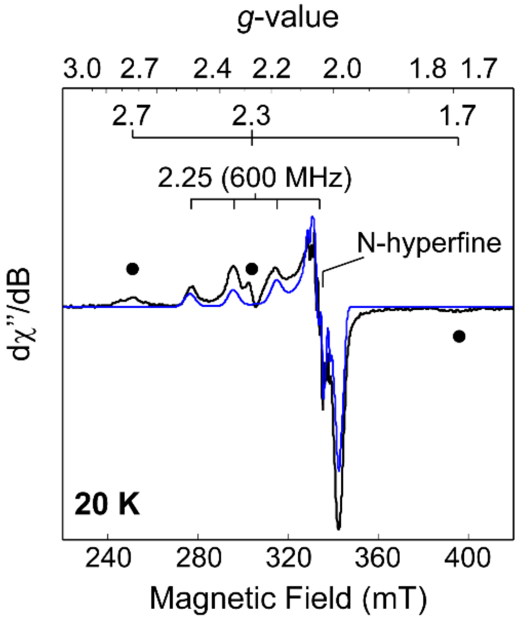

Fig. S6b 20 K CW X-band EPR spectra of {LS-4DCHIm + Cl-catechol}. EPR simulation (*blue line*) is overlaid on the experimental data (*black line*) for comparison. Simulation parameters: $S = 1/2$; $I = 3/2$ (Cu); $g_{1,2,3}$ (2.06, 2.07, 2.25); $A_{1,2,3}$ (-, -, 600 MHz); and s_B, 0.7 mT. A concentration of 1.1 ± 0.1 mM copper(II) was determined by quantitative simulation. This value faithfully reproduces values obtained by double integration of signal and comparison to known Cu(II)-standards (e.g., Cu(EDTA)). A sharp multiline feature is observed in the g ~ 2 region which is reasonably reproduced assuming two N-atoms (I = 1) with inequivalent hyperfine coupling $A_{1,2,3}$ (30, 80, 30 MHz) and $A'_{1,2,3}$ (30, 50, 30 MHz). An additional minority species (●) is observed with rhombic g-values of 2.75, 2.26, and 1.74 which can be attributed to ~0.2 mM. Quantitation of this feature for ~0.3 mM.

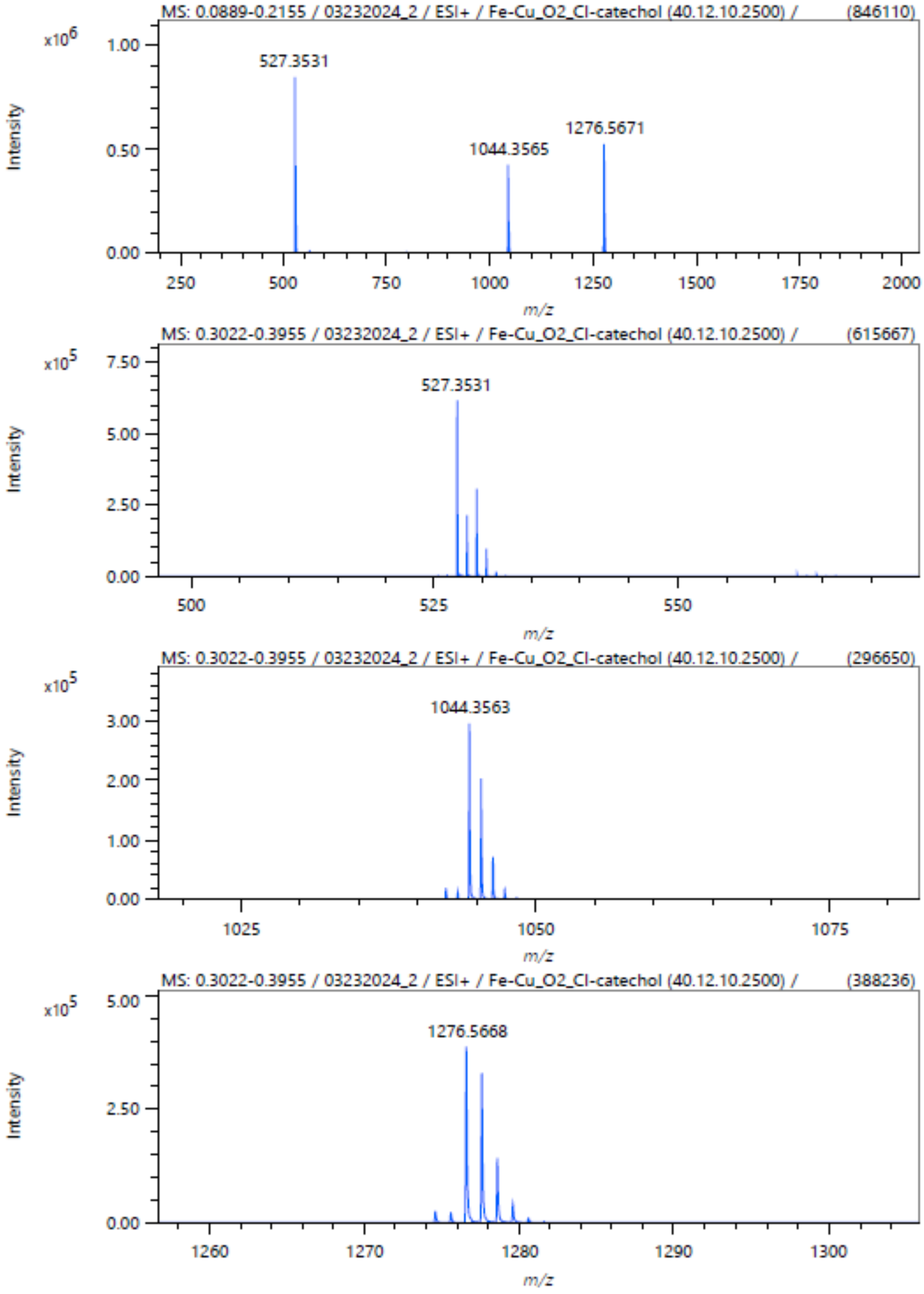

Fig. S7 Experimental ESI(+) mass spectra of **LS-4DCHIm** with 4-Cl-catechol. Masses at 527.35 ($[Cu(DCHIm)_2]^+$), 1044.36 ($[F_8Fe(DCHIm)]^+$), 1276.58 ($[F_8Fe(DCHIm)_2]^+$).

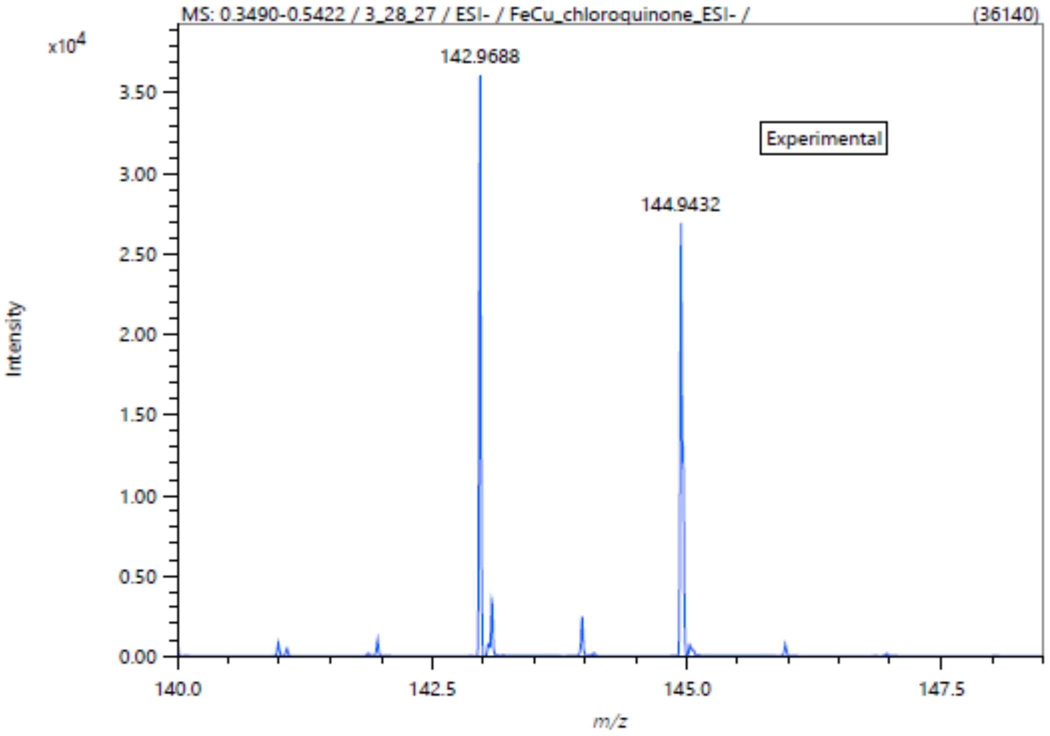

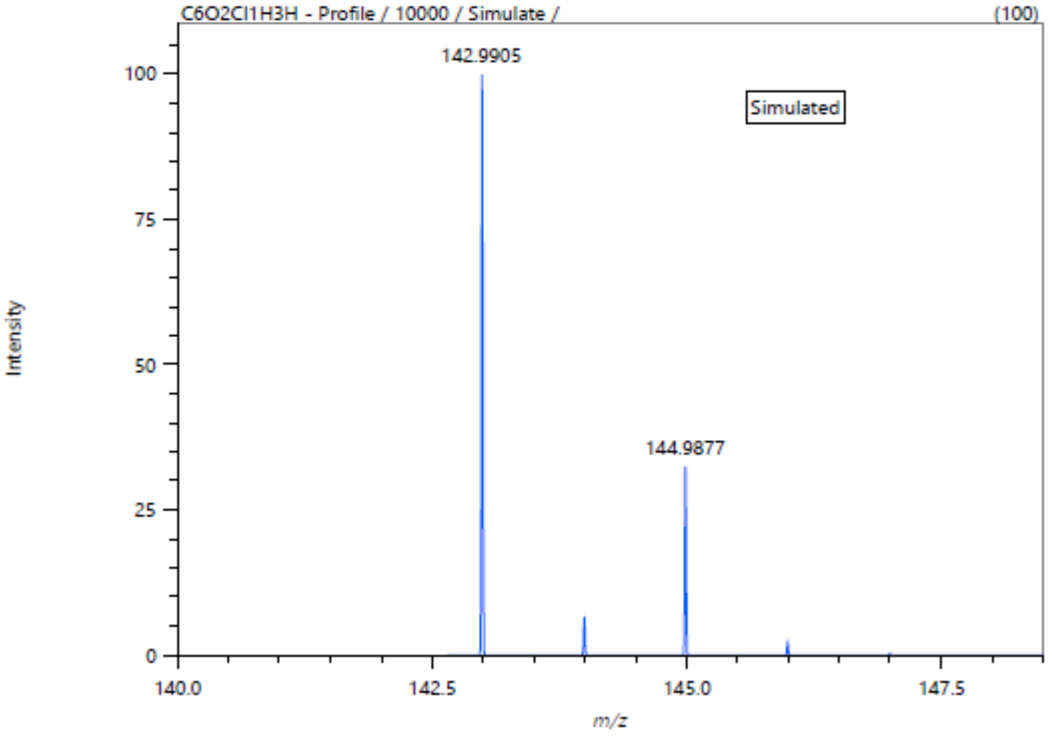

Fig. S8a Experimental and simulated ESI(-) mass spectra of **LS-4DCHIm** with 4-Cl-catechol. Mass at 142.97 suggests that 4-Cl-quinone.

The difference in isotopic patterns between the experimental and simulated spectra (Fig. S8a) is due to the presence of impurities in the experimental spectra (as shown below, where the peak at 144 has a hump corresponding to the impurity). It is sometime challenging to detect accurately the low mass region (m/z < 200), due to overlapping peaks.

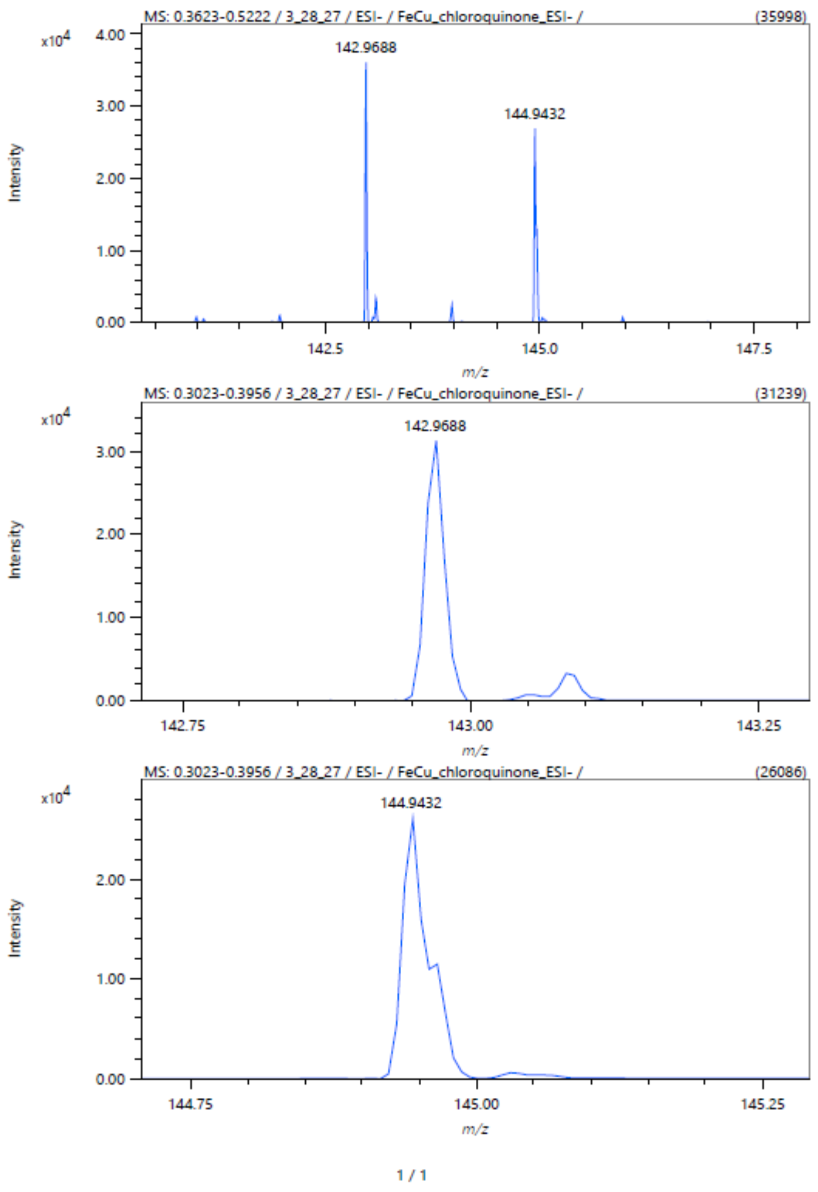

Fig. S8b Extrapolated peak for the experimental ESI(-) mass spectra of **LS-4DCHIm** with 4-Cl-catechol.

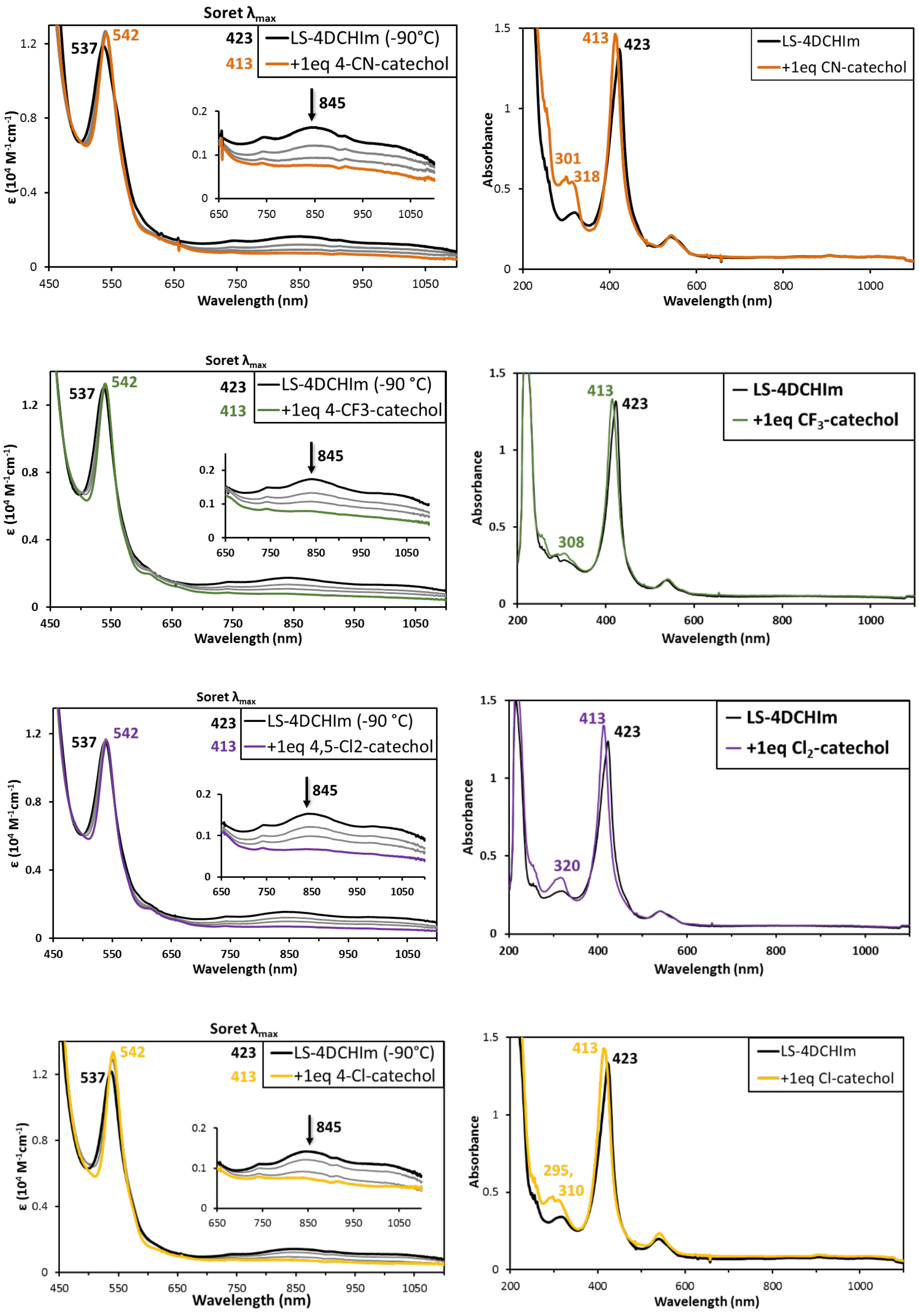

Fig. S9 UV-vis spectroscopic data showing the changes in the Q-band and low-energy regions (left) and Soret band region (right) for the reactions of **LS-4DCHIm** with the catechols proposed to induce O–O reductive cleavage chemistry (see Fig. 9, center pathway).

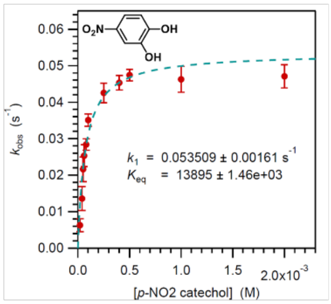

[4-NO$_2$-catechol] (M)	Average rate (s^{-1})
0.00002	0.006296
0.00004	0.013563
0.00005	0.021625
0.00006	0.025319
0.00008	0.028415
0.00010	0.035102
0.00025	0.042549
0.00040	0.045318
0.00050	0.047442
0.00100	0.046258
0.00200	0.047078

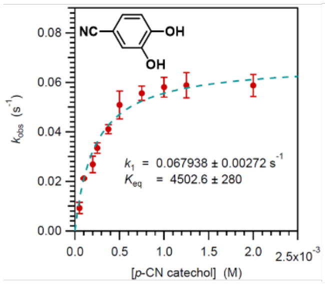

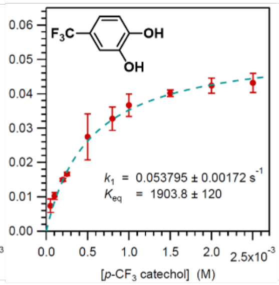

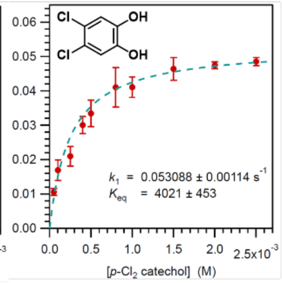

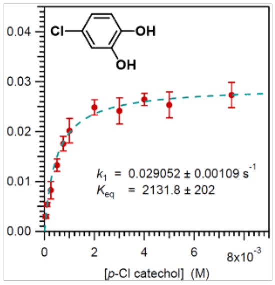

[4-CN-catechol] (M)	Average rate (s^{-1})
0.00005	0.009212
0.00010	0.021173
0.00020	0.026897
0.00025	0.033513
0.000375	0.041118
0.00050	0.050867
0.00075	0.055567
0.00100	0.058089
0.00125	0.058805
0.00200	0.058740

[4-CF$_3$-catechol] (M)	Average rate (s^{-1})
0.00005	0.007356
0.00010	0.010244
0.00020	0.014983
0.00025	0.016678
0.00050	0.027453
0.00080	0.032781
0.00100	0.036666
0.00150	0.040166
0.00200	0.042374
0.00250	0.043164

[4,5-(Cl)$_2$-catechol] (M)	Average rate (s^{-1})
0.00005	0.010565
0.00010	0.016918
0.00025	0.020994
0.00040	0.030067
0.00050	0.033456
0.00080	0.041027
0.00100	0.041060
0.00150	0.046421
0.00200	0.047469
0.00250	0.048538

[4-Cl-catechol] (M)	Average rate (s^{-1})
0.00005	0.002976
0.00010	0.005364
0.00025	0.008262
0.00050	0.013245
0.00075	0.017551
0.00100	0.020157
0.00200	0.024833
0.00300	0.024144
0.00400	0.026444
0.00500	0.025338
0.00750	0.027324

Fig. S10 Reaction rates, k_{obs}, as a function of catechol concentration for the reactions of {**LS-4DCHIm** + X-catechol}. The average rates were calculated based on a minimum of three trials, and the dotted line fits correspond to the equation in Fig. 7 and yield the parameters, k_1 and K_{eq} shown in the plots and in Table 2.

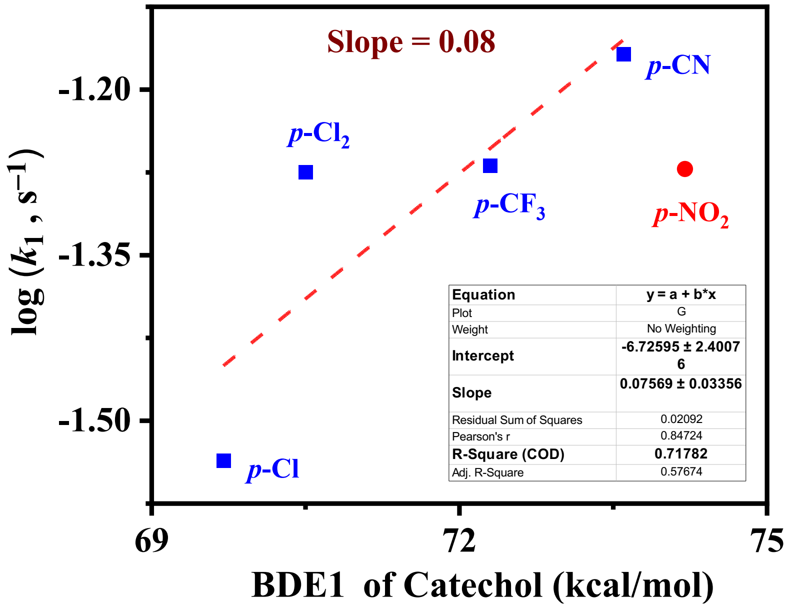

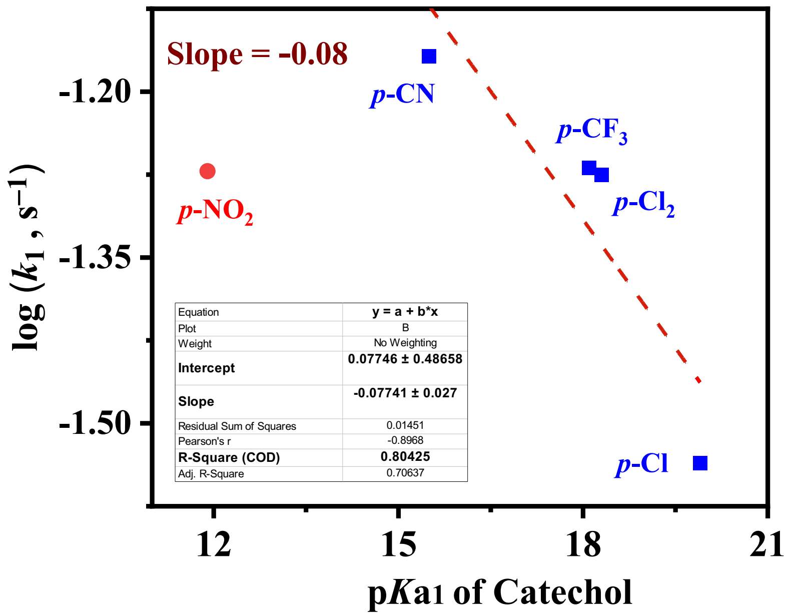

Fig. S11 Evans-Polanyi plot (top) and relationship between reaction rate and first p*K*a (bottom).

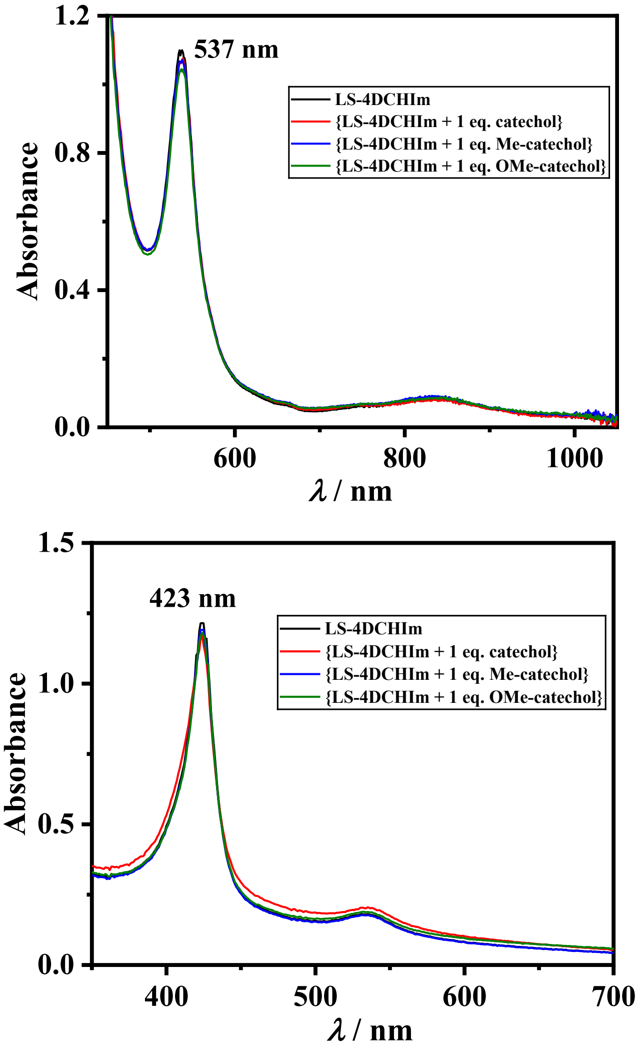

Fig. S12 UV-vis spectroscopic data showing no changes in the Q-band and low-energy regions (top; 0.1 mM, l = 1 cm) and Soret band region (bottom; 0.1 mM, l = 2 mm) for the reactions of **LS-4DCHIm** with the R-catechol (R = H, Me, OMe).

Table S1 Thermodynamic parameters for the catechols used in this study, calculated using DFT with a THF solvent model

Substrate	O–H BDE 1 (2) kcal/mol	p*K*a 1 (2) (considering no H-bond in the semiquinone form)	p*K*a 1 (2) (considering H-bond in the semiquinone form)	IP 1 kcal/mol (in THF/Gas phase)
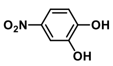	74.2 (69.3)	11.9 (31.4)	6.7 (36.6)	151.6/198.8
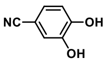	73.6 (68.7)	15.5 (33.5)	9.7 (39.3)	147.9/194.7
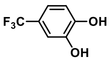	72.3 (66.7)	18.1 (35.0)	11.9 (41.2)	146.1/192.5
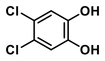	70.5 (65.6)	18.3 (33.5)	11.8 (39.8)	142.4/184.0
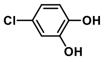	69.7 (65.5)	19.9 (36.3)	13.5 (42.6)	140.2/183.7
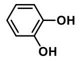	70.4 (65.3)	23.0 (38.5)	16.5 (45.1)	138.1/182.6
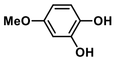	64.1 (62.7)	23.0 (40.8)	16.8 (47.0)	127.1/168.4
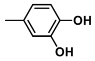	67.8 (64.1)	23.7 (39.2)	17.1 (45.8)	133.3/176.5

The DFT-calculated p*K*a values provide only qualitative insights for the comparison of different reactivities observed with different catechol substituents. Although CF_3- and Cl_2-substituted catechols (Table S1, 3rd and 4th rows) have similar first p*K*a values, their second p*K*a values differ, which may explain the variation in experimentally observed *K*eq values for these substrates. Therefore, although our plot (Fig. S11) is based on the first p*K*a, the second p*K*a may also play a significant role, given that the process involves $2H^+/2e^-$.

Chart 1. DFT-optimised cartesian coordinates for the lowest energy models

---

Catechol-H_2 ($S = 0$)

Zero-point correction=	0.108125 (Hartree/Particle)
Thermal correction to Energy=	0.114547
Thermal correction to Enthalpy=	0.115491
Thermal correction to Gibbs Free Energy=	0.077947
Sum of electronic and zero-point Energies=	-382.731607
Sum of electronic and thermal Energies=	-382.725185
Sum of electronic and thermal Enthalpies=	-382.724241
Sum of electronic and thermal Free Energies=	-382.761785

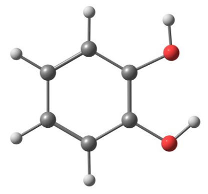

Cartesian Coordinates

C	-2.030858000	0.588463000	0.094687000
C	-0.634636000	0.636654000	0.001726000
C	0.022484000	1.867337000	-0.077852000
C	-0.716962000	3.057184000	-0.064378000
C	-2.106160000	3.007153000	0.029424000
C	-2.769401000	1.770475000	0.108439000
H	-2.557202000	-0.360360000	0.156829000
H	-0.066351000	-0.289544000	-0.007950000
H	1.105756000	1.910791000	-0.150587000
H	-0.216714000	4.021776000	-0.127171000
O	-4.134094000	1.717933000	0.198740000
H	-4.483044000	2.627636000	0.194792000
O	-2.925611000	4.113161000	0.052719000
H	-2.400014000	4.929656000	-0.006967000

[Catechol-H_2]$^{•+}$ ($S = 1/2$)

Zero-point correction=	0.108464 (Hartree/Particle)
Thermal correction to Energy=	0.114808
Thermal correction to Enthalpy=	0.115752
Thermal correction to Gibbs Free Energy=	0.077620
Sum of electronic and zero-point Energies=	-382.510827
Sum of electronic and thermal Energies=	-382.504483
Sum of electronic and thermal Enthalpies=	-382.503539
Sum of electronic and thermal Free Energies=	-382.541671

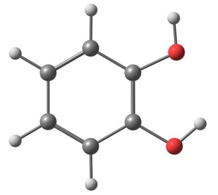

Cartesian Coordinates

C	-2.040239000	0.556424000	0.096948000

C	-0.669750000	0.628106000	0.006797000
C	0.005650000	1.893289000	-0.077740000
C	-0.692972000	3.086674000	-0.070083000
C	-2.086806000	3.037512000	0.026878000
C	-2.776277000	1.757020000	0.108448000
H	-2.568500000	-0.389194000	0.157422000
H	-0.079146000	-0.282181000	-0.003480000
H	1.088556000	1.906727000	-0.149614000
H	-0.184744000	4.044133000	-0.136894000
O	-4.092790000	1.717331000	0.193599000
H	-4.489783000	2.615467000	0.192646000
O	-2.902380000	4.086406000	0.055869000
H	-2.423625000	4.940601000	0.001656000

[Catechol-H]$^-$ (*S* = 0) (No H-bond)

Zero-point correction= 0.094286 (Hartree/Particle)
Thermal correction to Energy= 0.100521
Thermal correction to Enthalpy= 0.101465
Thermal correction to Gibbs Free Energy= 0.064171
Sum of electronic and zero-point Energies= -382.243269
Sum of electronic and thermal Energies= -382.237034
Sum of electronic and thermal Enthalpies= -382.236089
Sum of electronic and thermal Free Energies= -382.273383

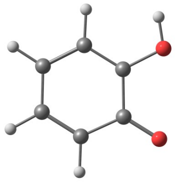

Cartesian Coordinates

C	-2.024594000	0.586361000	0.093140000
C	-0.628289000	0.643218000	-0.000304000
C	0.031745000	1.873692000	-0.077139000
C	-0.748989000	3.049045000	-0.060500000
C	-2.134761000	2.998760000	0.030665000
C	-2.866406000	1.748906000	0.117493000
H	-2.527013000	-0.379362000	0.152180000
H	-0.055216000	-0.284188000	-0.012365000
H	1.115194000	1.934890000	-0.148951000
H	-0.258761000	4.023534000	-0.120556000
O	-4.144853000	1.700106000	0.207154000
O	-2.897890000	4.157432000	0.044386000
H	-2.295789000	4.917954000	-0.018450000

[Catechol-H]$^-$ (*S* = 0) (H-bond)

Zero-point correction= 0.094947 (Hartree/Particle)
Thermal correction to Energy= 0.100909

Thermal correction to Enthalpy= 0.101853
Thermal correction to Gibbs Free Energy= 0.065069
Sum of electronic and zero-point Energies= -382.257697
Sum of electronic and thermal Energies= -382.251735
Sum of electronic and thermal Enthalpies= -382.250791
Sum of electronic and thermal Free Energies= -382.287575

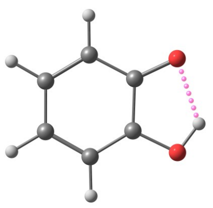

Cartesian Coordinates

C	-2.034890000	0.558169000	0.095561000
C	-0.624720000	0.619347000	0.000833000
C	0.000232000	1.866974000	-0.075789000
C	-0.749251000	3.058355000	-0.061991000
C	-2.164586000	3.039947000	0.031251000
C	-2.769283000	1.731122000	0.109703000
H	-2.548054000	-0.400883000	0.157065000
H	-0.040668000	-0.298376000	-0.010936000
H	1.086150000	1.923719000	-0.148575000
H	-0.247145000	4.023749000	-0.122826000
O	-4.138632000	1.753191000	0.200110000
H	-4.312479000	2.731065000	0.182547000
O	-2.962806000	4.064341000	0.052972000

[Catecholate]$^{2-}$ (S = 0)

Zero-point correction= 0.080650 (Hartree/Particle)
Thermal correction to Energy= 0.086636
Thermal correction to Enthalpy= 0.087580
Thermal correction to Gibbs Free Energy= 0.050685
Sum of electronic and zero-point Energies= -381.721348
Sum of electronic and thermal Energies= -381.715361
Sum of electronic and thermal Enthalpies= -381.714417
Sum of electronic and thermal Free Energies= -381.751313

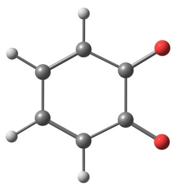

Cartesian Coordinates

C	-2.029143000	0.577485000	0.088278000
C	-0.613687000	0.621260000	-0.004128000
C	0.021275000	1.859608000	-0.072402000
C	-0.766938000	3.040187000	-0.057817000
C	-2.190313000	3.060555000	0.024588000
C	-2.872627000	1.727052000	0.124399000
H	-2.531420000	-0.392854000	0.144601000
H	-0.039676000	-0.307331000	-0.018645000
H	1.108862000	1.930270000	-0.138052000
H	-0.271471000	4.014099000	-0.114976000

O	-4.159325000	1.635241000	0.233488000
O	-2.866310000	4.165112000	0.020117000

[Catechol-H]$^{\bullet}$ ···H$^{\bullet}$ (S = 1)

Zero-point correction= 0.096192 (Hartree/Particle)
Thermal correction to Energy= 0.103950
Thermal correction to Enthalpy= 0.104894
Thermal correction to Gibbs Free Energy= 0.061277
Sum of electronic and zero-point Energies= -382.614705
Sum of electronic and thermal Energies= -382.606946
Sum of electronic and thermal Enthalpies= -382.606002
Sum of electronic and thermal Free Energies= -382.649620

Cartesian Coordinates

C	-2.060953000	0.556462000	0.146393000
C	-0.671975000	0.621138000	0.047203000
C	0.009945000	1.865363000	-0.086404000
C	-0.685625000	3.050349000	-0.121689000
C	-2.124204000	3.047452000	-0.023304000
C	-2.785982000	1.744266000	0.112709000
H	-2.576367000	-0.394263000	0.248106000
H	-0.094074000	-0.298475000	0.071682000
H	1.094022000	1.866184000	-0.160804000
H	-0.184349000	4.008834000	-0.223340000
O	-4.121121000	1.761653000	0.202306000
H	-4.380797000	2.711994000	0.153677000
O	-2.854605000	4.077908000	-0.045136000
H	-1.402255000	13.920623000	-1.050938000

[Quinone] ···H$^{\bullet}$ (S = 1/2)

Zero-point correction= 0.084432 (Hartree/Particle)
Thermal correction to Energy= 0.092290
Thermal correction to Enthalpy= 0.093234
Thermal correction to Gibbs Free Energy= 0.049594
Sum of electronic and zero-point Energies= -382.004843
Sum of electronic and thermal Energies= -381.996985
Sum of electronic and thermal Enthalpies= -381.996041
Sum of electronic and thermal Free Energies= -382.039681

Cartesian Coordinates

C	-2.018603000	0.501232000	0.063174000

C	-0.672813000	0.630938000	0.028772000
C	-0.010901000	1.936198000	-0.023529000
C	-0.701578000	3.098909000	-0.042056000
C	-2.168926000	3.087925000	-0.010411000
C	-2.876639000	1.691801000	0.050499000
H	-2.507209000	-0.468579000	0.102190000
H	-0.040153000	-0.253092000	0.039299000
H	1.075916000	1.947689000	-0.047950000
H	-0.208134000	4.066169000	-0.081771000
O	-4.098283000	1.637412000	0.085707000
O	-2.846560000	4.106275000	-0.029706000
H	-12.341303000	5.004576000	4.816651000

[Catechol-H]$^{\bullet}$ (*S* = 1/2)

Zero-point correction= 0.095994 (Hartree/Particle)
Thermal correction to Energy= 0.102031
Thermal correction to Enthalpy= 0.102976
Thermal correction to Gibbs Free Energy= 0.065344
Sum of electronic and zero-point Energies= -382.113055
Sum of electronic and thermal Energies= -382.107018
Sum of electronic and thermal Enthalpies= -382.106073
Sum of electronic and thermal Free Energies= -382.143705

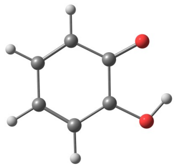

Cartesian Coordinates

C	-2.048157000	0.550864000	0.096988000
C	-0.660070000	0.638857000	0.003470000
C	0.004494000	1.896960000	-0.077603000
C	-0.707650000	3.072545000	-0.065553000
C	-2.146136000	3.045642000	0.030558000
C	-2.789825000	1.728785000	0.110799000
H	-2.550453000	-0.410298000	0.158089000
H	-0.069390000	-0.272861000	-0.008279000
H	1.088544000	1.916075000	-0.149962000
H	-0.219800000	4.041329000	-0.126612000
O	-4.125275000	1.723512000	0.197736000
H	-4.398521000	2.671236000	0.188638000
O	-2.890556000	4.066014000	0.051149000

Quinone (*S* = 0)

Zero-point correction= 0.084263 (Hartree/Particle)
Thermal correction to Energy= 0.090399
Thermal correction to Enthalpy= 0.091344
Thermal correction to Gibbs Free Energy= 0.053900
Sum of electronic and zero-point Energies= -381.503147
Sum of electronic and thermal Energies= -381.497011
Sum of electronic and thermal Enthalpies= -381.496067
Sum of electronic and thermal Free Energies= -381.533510

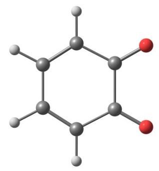

Cartesian Coordinates

C	-2.032489000	0.515512000	0.096335000
C	-0.687487000	0.624311000	0.005221000
C	-0.008539000	1.919239000	-0.076771000
C	-0.681468000	3.092476000	-0.067006000
C	-2.146014000	3.104256000	0.028073000
C	-2.871976000	1.719104000	0.117472000
H	-2.534164000	-0.446500000	0.156830000
H	-0.068687000	-0.269376000	-0.009778000
H	1.076186000	1.914070000	-0.148191000
H	-0.175144000	4.051978000	-0.128506000
O	-4.091926000	1.682984000	0.201090000
O	-2.808041000	4.132969000	0.039856000

[H•] (*S* = 1/2)

Zero-point correction= 0.000000 (Hartree/Particle)
Thermal correction to Energy= 0.001416
Thermal correction to Enthalpy= 0.002360
Thermal correction to Gibbs Free Energy= -0.010654
Sum of electronic and zero-point Energies= -0.501867
Sum of electronic and thermal Energies= -0.500451
Sum of electronic and thermal Enthalpies= -0.499507
Sum of electronic and thermal Free Energies= -0.512521

Cartesian Coordinates

H	-12.341303000	5.004576000	4.816651000

[PhO•] (*S* = 1/2)

Zero-point correction= 0.090829 (Hartree/Particle)
Thermal correction to Energy= 0.096035
Thermal correction to Enthalpy= 0.096979
Thermal correction to Gibbs Free Energy= 0.062018
Sum of electronic and zero-point Energies= -306.851510
Sum of electronic and thermal Energies= -306.846304
Sum of electronic and thermal Enthalpies= -306.845360
Sum of electronic and thermal Free Energies= -306.880321

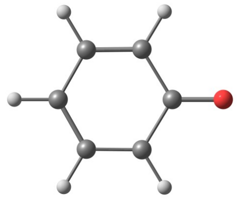

Cartesian Coordinates

C	-0.201774000	0.000000000	-2.851842000
C	-1.375458000	-0.360163000	-2.152911000
C	0.971911000	0.360163000	-2.152911000
H	-2.267648000	-0.633787000	-2.710397000
H	1.864100000	0.633787000	-2.710397000
C	-1.389299000	-0.364387000	-0.773621000
C	0.985752000	0.364387000	-0.773621000
H	-2.278760000	-0.637208000	-0.212051000
H	1.875213000	0.637208000	-0.212051000
C	-0.201774000	0.000000000	-0.019347000
H	-0.201774000	0.000000000	-3.938471000
O	-0.201774000	0.000000000	1.244357000

[PhO$^-$] (S = 0)

Zero-point correction= 0.090080 (Hartree/Particle)
Thermal correction to Energy= 0.095232
Thermal correction to Enthalpy= 0.096176
Thermal correction to Gibbs Free Energy= 0.061319
Sum of electronic and zero-point Energies= -306.998680
Sum of electronic and thermal Energies= -306.993528
Sum of electronic and thermal Enthalpies= -306.992584
Sum of electronic and thermal Free Energies= -307.027441

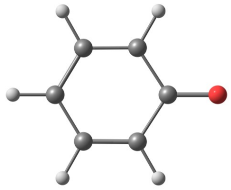

Cartesian Coordinates

C	-2.029911000	0.600002000	0.094423000
C	-0.639735000	0.648169000	0.001422000
C	0.048232000	1.871600000	-0.079474000
C	-0.711859000	3.054284000	-0.064454000
C	-2.102495000	3.024614000	0.028419000
C	-2.846473000	1.790407000	0.113212000
H	-2.540502000	-0.361347000	0.155110000

H	-0.076578000	-0.286140000	-0.008375000
H	1.133130000	1.902243000	-0.151665000
H	-0.205306000	4.018594000	-0.125921000
O	-4.127059000	1.754642000	0.198354000
H	-2.669915000	3.955427000	0.038005000

PhOH (S = 0)

Zero-point correction= 0.103759 (Hartree/Particle)
Thermal correction to Energy= 0.109118
Thermal correction to Enthalpy= 0.110062
Thermal correction to Gibbs Free Energy= 0.074899
Sum of electronic and zero-point Energies= -307.482538
Sum of electronic and thermal Energies= -307.477179
Sum of electronic and thermal Enthalpies= -307.476234
Sum of electronic and thermal Free Energies= -307.511397

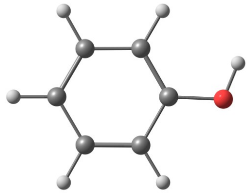

Cartesian Coordinates

C	-2.036572000	0.603108000	0.094554000
C	-0.643995000	0.646993000	0.001646000
C	0.029086000	1.872438000	-0.078405000
C	-0.708799000	3.060181000	-0.064599000
C	-2.103959000	3.030336000	0.028321000
C	-2.765368000	1.798703000	0.107429000
H	-2.565247000	-0.344272000	0.157014000
H	-0.082546000	-0.284190000	-0.007859000
H	1.113022000	1.900127000	-0.150682000
H	-0.201391000	4.019927000	-0.126228000
O	-4.130260000	1.703899000	0.199448000
H	-4.523182000	2.594402000	0.199598000
H	-2.675321000	3.956936000	0.039070000

---

^{NO2}Catechol-H_2 (S = 0)

Zero-point correction= 0.110609 (Hartree/Particle)
Thermal correction to Energy= 0.119377
Thermal correction to Enthalpy= 0.120321
Thermal correction to Gibbs Free Energy= 0.076756
Sum of electronic and zero-point Energies= -587.311141
Sum of electronic and thermal Energies= -587.302374
Sum of electronic and thermal Enthalpies= -587.301429
Sum of electronic and thermal Free Energies= -587.344994

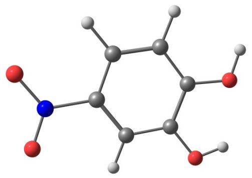

Cartesian Coordinates

C	-2.040897000	0.583394000	0.091007000
C	-0.645075000	0.657575000	0.001854000
C	0.032823000	1.876122000	-0.074564000
C	-0.709346000	3.054524000	-0.060785000
C	-2.101190000	3.002611000	0.029965000
C	-2.771281000	1.762379000	0.104609000
H	-2.548863000	-0.371748000	0.148540000
H	1.113041000	1.904182000	-0.143218000
H	-0.207419000	4.016893000	-0.121172000
O	-4.129107000	1.710421000	0.190017000
H	-4.486995000	2.617157000	0.187659000
O	-2.911928000	4.096597000	0.054227000
H	-2.394210000	4.920317000	-0.000675000
N	0.120733000	-0.581479000	-0.011919000
O	-0.493765000	-1.654176000	0.050305000
O	1.354694000	-0.510590000	-0.085401000

[^{NO2}Catechol-H$_2$]$^{\bullet+}$ (S = 1/2)

Zero-point correction= 0.110660 (Hartree/Particle)
Thermal correction to Energy= 0.119370
Thermal correction to Enthalpy= 0.120314
Thermal correction to Gibbs Free Energy= 0.076098
Sum of electronic and zero-point Energies= -587.068771
Sum of electronic and thermal Energies= -587.060061
Sum of electronic and thermal Enthalpies= -587.059116
Sum of electronic and thermal Free Energies= -587.103332

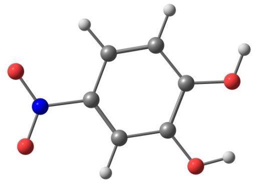

Cartesian Coordinates

C	-2.041140000	0.549207000	0.101161000
C	-0.679363000	0.644873000	0.012458000
C	0.014688000	1.892695000	-0.065501000
C	-0.688404000	3.080927000	-0.061562000
C	-2.083197000	3.040272000	0.027626000
C	-2.774693000	1.760576000	0.109101000
H	-2.561248000	-0.400257000	0.156944000
H	1.096502000	1.898326000	-0.129379000
H	-0.171260000	4.033241000	-0.126447000
O	-4.083457000	1.708193000	0.187326000
H	-4.498816000	2.600543000	0.181752000
O	-2.894669000	4.083597000	0.049082000
H	-2.421672000	4.942715000	-0.006875000

N	0.117582000	-0.600706000	-0.016235000
O	-0.488814000	-1.666884000	0.043620000
O	1.339176000	-0.483140000	-0.102621000

[^{NO2}Catechol-H]$^{-}$ (*S* = 0) (No H-bond)

Zero-point correction= 0.097488 (Hartree/Particle)
Thermal correction to Energy= 0.106030
Thermal correction to Enthalpy= 0.106974
Thermal correction to Gibbs Free Energy= 0.063760
Sum of electronic and zero-point Energies= -586.847107
Sum of electronic and thermal Energies= -586.838565
Sum of electronic and thermal Enthalpies= -586.837621
Sum of electronic and thermal Free Energies= -586.880835

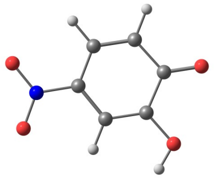

Cartesian Coordinates

C	-2.046107000	0.577943000	0.112773000
C	-0.628625000	0.628571000	0.006381000
C	0.022960000	1.879019000	-0.080882000
C	-0.716704000	3.040858000	-0.062357000
C	-2.160497000	3.060108000	0.044541000
C	-2.782982000	1.735867000	0.131249000
H	-2.534791000	-0.390216000	0.178540000
H	1.104197000	1.909309000	-0.162252000
H	-0.221215000	4.006948000	-0.129166000
O	-4.153409000	1.735903000	0.232256000
H	-4.463474000	0.815092000	0.283882000
O	-2.836433000	4.123382000	0.062326000
N	0.113765000	-0.563505000	-0.010795000
O	-0.489395000	-1.667590000	0.069212000
O	1.369514000	-0.515573000	-0.105530000

[^{NO2}Catechol-H]$^{-}$ (*S* = 0) (H-bond)

Zero-point correction= 0.097897 (Hartree/Particle)
Thermal correction to Energy= 0.106237
Thermal correction to Enthalpy= 0.107181
Thermal correction to Gibbs Free Energy= 0.064356
Sum of electronic and zero-point Energies= -586.858538
Sum of electronic and thermal Energies= -586.850197
Sum of electronic and thermal Enthalpies= -586.849253
Sum of electronic and thermal Free Energies= -586.892079

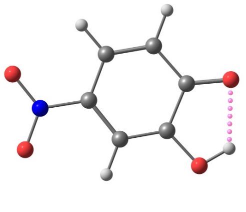

Cartesian Coordinates

C	-2.049024000	0.552075000	0.091606000
C	-0.631015000	0.631785000	0.000888000
C	0.014989000	1.883889000	-0.072491000
C	-0.724124000	3.053238000	-0.057507000
C	-2.153784000	3.032403000	0.032592000
C	-2.773273000	1.715106000	0.106456000
H	-2.539577000	-0.413120000	0.147042000
H	1.096749000	1.916276000	-0.141177000
H	-0.227749000	4.019265000	-0.113865000
O	-4.135317000	1.732107000	0.191756000
H	-4.345409000	2.696774000	0.179468000
O	-2.917916000	4.051162000	0.053245000
N	0.134599000	-0.554977000	-0.014569000
O	-0.452880000	-1.663934000	0.051639000
O	1.387609000	-0.485929000	-0.093747000

[^{NO2}Catecholate]$^{2-}$ (*S* = 0)

Zero-point correction= 0.083933 (Hartree/Particle)
Thermal correction to Energy= 0.092294
Thermal correction to Enthalpy= 0.093238
Thermal correction to Gibbs Free Energy= 0.050201
Sum of electronic and zero-point Energies= -586.340479
Sum of electronic and thermal Energies= -586.332117
Sum of electronic and thermal Enthalpies= -586.331173
Sum of electronic and thermal Free Energies= -586.374211

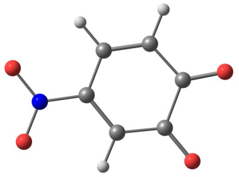

Cartesian Coordinates

C	-2.059103000	0.558378000	0.089752000
C	-0.633811000	0.619755000	0.000182000
C	0.018755000	1.874430000	-0.070369000
C	-0.738251000	3.028647000	-0.056201000
C	-2.184066000	3.040237000	0.029837000
C	-2.886778000	1.702253000	0.116956000
H	-2.534552000	-0.416546000	0.140563000
H	1.101081000	1.910571000	-0.135309000
H	-0.248847000	4.001682000	-0.111065000
O	-4.166556000	1.656907000	0.207993000
O	-2.825832000	4.129399000	0.032859000
N	0.136397000	-0.542143000	-0.015970000
O	-0.437653000	-1.676154000	0.047647000
O	1.411424000	-0.481562000	-0.093425000

[^{NO2}Catechol-H]$^{\bullet}$ ···H$^{\bullet}$ (S = 1)

Zero-point correction= 0.098381 (Hartree/Particle)
Thermal correction to Energy= 0.108545
Thermal correction to Enthalpy= 0.109489
Thermal correction to Gibbs Free Energy= 0.059442
Sum of electronic and zero-point Energies= -587.187807
Sum of electronic and thermal Energies= -587.177644
Sum of electronic and thermal Enthalpies= -587.176699
Sum of electronic and thermal Free Energies= -587.226746

Cartesian Coordinates

C	0.540694000	0.092610000	-4.468074000
C	-0.533429000	-0.314552000	-3.711165000
C	1.762685000	0.450396000	-3.797639000
C	-0.489577000	-0.398648000	-2.295248000
C	1.785402000	0.359220000	-2.332809000
H	-1.367974000	-0.728176000	-1.753533000
C	0.669646000	-0.060977000	-1.609970000
H	0.711571000	-0.122195000	-0.527152000
H	0.496531000	0.152122000	-5.549065000
O	2.920911000	0.695495000	-1.730030000
H	3.554622000	0.953144000	-2.441094000
N	-1.781917000	-0.678815000	-4.391680000
O	-2.732229000	-1.053013000	-3.696972000
O	-1.821718000	-0.593267000	-5.621798000
O	2.809205000	0.835406000	-4.385059000
H	9.928163000	-1.960195000	-10.827500000

[^{NO2}Quinone] ···H$^{\bullet}$ (S = 1/2)

Zero-point correction= 0.086611 (Hartree/Particle)
Thermal correction to Energy= 0.096906
Thermal correction to Enthalpy= 0.097850
Thermal correction to Gibbs Free Energy= 0.047406
Sum of electronic and zero-point Energies= -586.571108
Sum of electronic and thermal Energies= -586.560813
Sum of electronic and thermal Enthalpies= -586.559869
Sum of electronic and thermal Free Energies= -586.610313

Cartesian Coordinates

C	-1.993919000	0.422640000	0.005523000
C	-0.666048000	0.648978000	0.008350000

C	-0.050108000	1.972184000	0.030358000
C	-0.809589000	3.088102000	0.053084000
C	-2.275809000	3.007415000	0.058342000
C	-2.910256000	1.581549000	0.023288000
H	-2.421044000	-0.573768000	-0.011704000
H	1.031910000	2.032028000	0.028176000
H	-0.362046000	4.077358000	0.070616000
O	-4.121129000	1.445858000	0.009932000
O	-3.004374000	3.986376000	0.088507000
H	-12.134629000	5.320244000	4.747016000
N	0.247175000	-0.522016000	-0.012709000
O	-0.249868000	-1.646489000	-0.022896000
O	1.457548000	-0.293283000	-0.018739000

[^{NO2}Catechol-H]$^{\bullet}$ (*S* = 1/2)

Zero-point correction= 0.098100 (Hartree/Particle)
Thermal correction to Energy= 0.106581
Thermal correction to Enthalpy= 0.107526
Thermal correction to Gibbs Free Energy= 0.063599
Sum of electronic and zero-point Energies= -586.686189
Sum of electronic and thermal Energies= -586.677708
Sum of electronic and thermal Enthalpies= -586.676764
Sum of electronic and thermal Free Energies= -586.720691

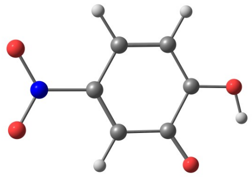

Cartesian Coordinates

C	0.577846000	0.018961000	-4.460735000
C	-0.521523000	-0.333284000	-3.712726000
C	1.786231000	0.398469000	-3.777554000
C	-0.516522000	-0.340459000	-2.293683000
C	1.769781000	0.383845000	-2.309770000
H	-1.413923000	-0.627347000	-1.758946000
C	0.629171000	0.018014000	-1.596295000
H	0.641065000	0.014769000	-0.510966000
H	0.563599000	0.020127000	-5.544227000
O	2.894313000	0.735763000	-1.695103000
H	3.549870000	0.947653000	-2.401364000
N	-1.756663000	-0.718033000	-4.406504000
O	-2.733990000	-1.030300000	-3.718703000
O	-1.758852000	-0.710521000	-5.640202000
O	2.852028000	0.740707000	-4.356568000

[^{NO2}Quinone] (*S* = 0)

Zero-point correction= 0.086384 (Hartree/Particle)
Thermal correction to Energy= 0.094911
Thermal correction to Enthalpy= 0.095856
Thermal correction to Gibbs Free Energy= 0.052285
Sum of electronic and zero-point Energies= -586.069426
Sum of electronic and thermal Energies= -586.060899
Sum of electronic and thermal Enthalpies= -586.059955
Sum of electronic and thermal Free Energies= -586.103525

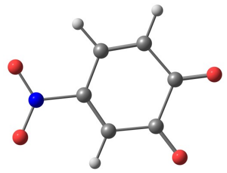

Cartesian Coordinates

C	-2.044129000	0.508658000	0.096276000
C	-0.706682000	0.639209000	0.002847000
C	-0.002065000	1.914776000	-0.081156000
C	-0.681094000	3.081700000	-0.070017000
C	-2.145871000	3.106351000	0.030586000
C	-2.876103000	1.729494000	0.119633000
H	-2.538501000	-0.454467000	0.156219000
H	1.078911000	1.897678000	-0.155703000
H	-0.166601000	4.035721000	-0.135497000
O	-4.090634000	1.679976000	0.204733000
O	-2.802818000	4.135107000	0.046942000
N	0.121381000	-0.593566000	-0.010932000
O	-0.454057000	-1.680064000	-0.018068000
O	1.344978000	-0.451049000	-0.011398000

---

^{CN}Catechol-H$_2$ (*S* = 0)

Zero-point correction= 0.107106 (Hartree/Particle)
Thermal correction to Energy= 0.115108
Thermal correction to Enthalpy= 0.116052
Thermal correction to Gibbs Free Energy= 0.074568
Sum of electronic and zero-point Energies= -475.010470
Sum of electronic and thermal Energies= -475.002469
Sum of electronic and thermal Enthalpies= -475.001524
Sum of electronic and thermal Free Energies= -475.043009

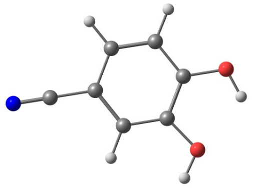

Cartesian Coordinates

C	0.576282000	0.009761000	-4.423114000
C	-0.579020000	-0.351522000	-3.699092000
C	1.725044000	0.365715000	-3.732895000
C	-0.566623000	-0.351421000	-2.293647000
C	1.740688000	0.365664000	-2.322967000
H	-1.458646000	-0.629855000	-1.742006000
C	0.594536000	0.007201000	-1.612894000

H	0.623433000	0.012679000	-0.527376000
H	0.566807000	0.008731000	-5.509870000
O	2.866310000	0.712856000	-1.648180000
H	3.569390000	0.932725000	-2.287685000
O	2.905733000	0.735833000	-4.322204000
H	2.834151000	0.715718000	-5.292993000
C	-1.766596000	-0.718890000	-4.405390000
N	-2.735303000	-1.018223000	-4.980236000

[^{CN}Catechol-H_2]$^{\bullet+}$ (*S* = 1/2)

Zero-point correction= 0.106936 (Hartree/Particle)
Thermal correction to Energy= 0.114994
Thermal correction to Enthalpy= 0.115938
Thermal correction to Gibbs Free Energy= 0.073471
Sum of electronic and zero-point Energies= -474.773826
Sum of electronic and thermal Energies= -474.765769
Sum of electronic and thermal Enthalpies= -474.764825
Sum of electronic and thermal Free Energies= -474.807292

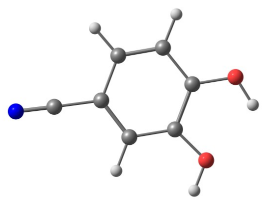

Cartesian Coordinates

C	0.570911000	0.008118000	-4.464797000
C	-0.553127000	-0.343256000	-3.725608000
C	1.724907000	0.371971000	-3.767015000
C	-0.546448000	-0.338476000	-2.280862000
C	1.741982000	0.364131000	-2.310660000
H	-1.452955000	-0.615407000	-1.752923000
C	0.584131000	0.007950000	-1.588886000
H	0.612125000	0.014453000	-0.504579000
H	0.558976000	0.001043000	-5.550768000
O	2.835477000	0.687727000	-1.658489000
H	3.578820000	0.916359000	-2.260086000
O	2.876477000	0.741461000	-4.311320000
H	2.859180000	0.735710000	-5.292948000
C	-1.754113000	-0.722136000	-4.398739000
N	-2.740157000	-1.032674000	-4.932870000

[^{CN}Catechol-H]$^{-}$ (*S* = 0) (No H-bond)

Zero-point correction= 0.093750 (Hartree/Particle)

Thermal correction to Energy= 0.101576
Thermal correction to Enthalpy= 0.102520
Thermal correction to Gibbs Free Energy= 0.061178
Sum of electronic and zero-point Energies= -474.538427
Sum of electronic and thermal Energies= -474.530600
Sum of electronic and thermal Enthalpies= -474.529656
Sum of electronic and thermal Free Energies= -474.570998

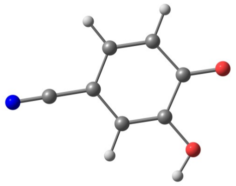

Cartesian Coordinates

C	0.600309000	0.017446000	-4.401895000
C	-0.587053000	-0.353515000	-3.704184000
C	1.743077000	0.373659000	-3.719297000
C	-0.568139000	-0.345469000	-2.290940000
C	1.813076000	0.379801000	-2.261322000
H	-1.466924000	-0.620108000	-1.742859000
C	0.582926000	0.007867000	-1.606847000
H	0.589781000	0.010982000	-0.518448000
H	0.597573000	0.018156000	-5.491724000
O	2.871081000	0.687879000	-1.635036000
O	2.899808000	0.744358000	-4.372744000
H	2.744404000	0.698230000	-5.331784000
C	-1.752758000	-0.720976000	-4.415020000
N	-2.719467000	-1.026164000	-5.004961000

[^{CN}Catechol-H]$^{-}$ (*S* = 0) (H-bond)

Zero-point correction= 0.094217 (Hartree/Particle)
Thermal correction to Energy= 0.101802
Thermal correction to Enthalpy= 0.102746
Thermal correction to Gibbs Free Energy= 0.061916
Sum of electronic and zero-point Energies= -474.551296
Sum of electronic and thermal Energies= -474.543711
Sum of electronic and thermal Enthalpies= -474.542767
Sum of electronic and thermal Free Energies= -474.583597

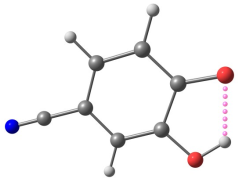

Cartesian Coordinates

C	0.561501000	0.009156000	-4.359992000
C	-0.612307000	-0.351780000	-3.663387000
C	1.718950000	0.362460000	-3.673190000
H	2.617636000	0.638598000	-4.221013000
C	-0.609108000	-0.352983000	-2.235653000
C	1.765682000	0.374192000	-2.247657000
H	-1.505199000	-0.628181000	-1.684812000

C	0.539328000	-0.001927000	-1.567586000
H	0.555043000	0.009215000	-5.447543000
O	0.620554000	0.022002000	-0.203047000
H	1.556586000	0.308073000	-0.056015000
O	2.775889000	0.682089000	-1.519974000
C	-1.784942000	-0.709449000	-4.374359000
N	-2.754237000	-1.004459000	-4.962848000

[^{CN}Catecholate]$^{2-}$ (*S* = 0)

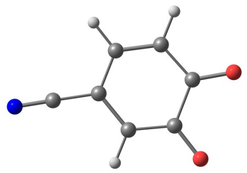

Zero-point correction= 0.080004 (Hartree/Particle)
Thermal correction to Energy= 0.087671
Thermal correction to Enthalpy= 0.088615
Thermal correction to Gibbs Free Energy= 0.047477
Sum of electronic and zero-point Energies= -474.027284
Sum of electronic and thermal Energies= -474.019617
Sum of electronic and thermal Enthalpies= -474.018673
Sum of electronic and thermal Free Energies= -474.059811

Cartesian Coordinates

C	0.550469000	-0.014998000	-4.416897000
C	-0.616561000	-0.349204000	-3.701391000
C	1.741303000	0.333321000	-3.693154000
H	-1.527745000	-0.617182000	-4.234274000
H	2.639815000	0.591752000	-4.254806000
C	-0.584486000	-0.334463000	-2.304703000
C	1.828686000	0.361410000	-2.280431000
H	-1.485578000	-0.593845000	-1.745621000
C	0.575544000	-0.000150000	-1.524924000
O	0.548091000	-0.008253000	-0.247975000
O	2.910061000	0.675048000	-1.654191000
C	0.547746000	-0.033250000	-5.827942000
N	0.559549000	-0.045361000	-7.005250000

[^{CN}Catechol-H]$^{\bullet}$···H$^{\bullet}$ (*S* = 1)

Zero-point correction= 0.094902 (Hartree/Particle)
Thermal correction to Energy= 0.104287
Thermal correction to Enthalpy= 0.105231
Thermal correction to Gibbs Free Energy= 0.057332
Sum of electronic and zero-point Energies= -474.888078
Sum of electronic and thermal Energies= -474.878693
Sum of electronic and thermal Enthalpies= -474.877749

Sum of electronic and thermal Free Energies= -474.925648

Cartesian Coordinates

C	0.631379000	0.013712000	-4.380005000
C	-0.487524000	-0.512605000	-3.658492000
C	1.731735000	0.529186000	-3.718463000
H	-1.332305000	-0.907987000	-4.213267000
H	2.585628000	0.930582000	-4.254612000
C	-0.505112000	-0.523159000	-2.270907000
C	1.756702000	0.538724000	-2.280063000
H	-1.355228000	-0.923255000	-1.726988000
C	0.590790000	-0.009503000	-1.578784000
O	0.631149000	0.007989000	-0.248399000
H	1.495402000	0.413630000	0.000445000
O	2.710369000	0.984649000	-1.585584000
C	0.599872000	0.001585000	-5.813442000
N	0.567455000	-0.011060000	-6.976844000
H	9.492077000	3.763584000	5.217795000

[^{CN}Quinone] ···H$^{•}$ (S = 1/2)

Zero-point correction= 0.083242 (Hartree/Particle)
Thermal correction to Energy= 0.092558
Thermal correction to Enthalpy= 0.093502
Thermal correction to Gibbs Free Energy= 0.046447
Sum of electronic and zero-point Energies= -474.273148
Sum of electronic and thermal Energies= -474.263832
Sum of electronic and thermal Enthalpies= -474.262888
Sum of electronic and thermal Free Energies= -474.309943

Cartesian Coordinates

C	1.185757000	0.146547000	-2.334364000
C	0.763572000	-0.471788000	-3.603002000
C	0.340845000	0.317766000	-1.285216000
H	1.509537000	-0.567009000	-4.386583000
H	0.658257000	0.775371000	-0.353248000
C	-0.498964000	-0.906725000	-3.795438000
C	-1.058644000	-0.126028000	-1.393613000
H	-0.807113000	-1.362344000	-4.732081000
C	-1.509603000	-0.781074000	-2.737083000
O	-2.663654000	-1.160189000	-2.864180000
O	-1.854483000	0.003608000	-0.477228000

C	2.551620000	0.576789000	-2.226323000
N	3.660598000	0.919881000	-2.156746000
H	6.758924000	1.749208000	-1.197228000

[^{CN}Catechol-H]$^{\bullet}$ (*S* = 1/2)

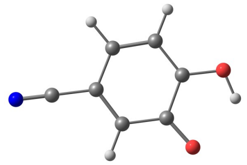

Zero-point correction= 0.094774 (Hartree/Particle)
Thermal correction to Energy= 0.102404
Thermal correction to Enthalpy= 0.103349
Thermal correction to Gibbs Free Energy= 0.061766
Sum of electronic and zero-point Energies= -474.386350
Sum of electronic and thermal Energies= -474.378720
Sum of electronic and thermal Enthalpies= -474.377776
Sum of electronic and thermal Free Energies= -474.419358

Cartesian Coordinates

C	0.641531000	0.016240000	-4.377366000
C	-0.482614000	-0.515772000	-3.668369000
C	1.733457000	0.533731000	-3.703554000
H	-1.320420000	-0.912725000	-4.232482000
H	2.591224000	0.939376000	-4.230212000
C	-0.514076000	-0.529969000	-2.281077000
C	1.743910000	0.539952000	-2.264957000
H	-1.368235000	-0.934538000	-1.746900000
C	0.572978000	-0.014158000	-1.576740000
O	0.599822000	0.000245000	-0.245976000
H	1.460025000	0.408345000	0.012619000
O	2.688805000	0.987939000	-1.559868000
C	0.624193000	0.007649000	-5.811077000
N	0.603387000	-0.002042000	-6.974772000

[^{CN}Quinone] (*S* = 0)

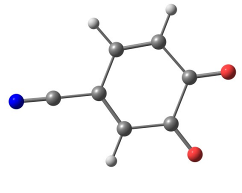

Zero-point correction= 0.082968 (Hartree/Particle)
Thermal correction to Energy= 0.090702
Thermal correction to Enthalpy= 0.091646
Thermal correction to Gibbs Free Energy= 0.050211
Sum of electronic and zero-point Energies= -473.771672
Sum of electronic and thermal Energies= -473.763938
Sum of electronic and thermal Enthalpies= -473.762994
Sum of electronic and thermal Free Energies= -473.804429

Cartesian Coordinates

C	0.654347000	0.042586000	-4.399622000
C	-0.507140000	-0.515826000	-3.686155000
C	1.733311000	0.556841000	-3.755106000
H	-1.325552000	-0.902257000	-4.286177000
H	2.585413000	0.968554000	-4.287283000
C	-0.562549000	-0.552882000	-2.338793000
C	1.767056000	0.570133000	-2.283164000
H	-1.419061000	-0.967917000	-1.815377000
C	0.543613000	-0.033327000	-1.524776000
O	0.544446000	-0.054871000	-0.303701000
O	2.707498000	1.033478000	-1.658128000
C	0.620277000	0.031957000	-5.835375000
N	0.576090000	0.015418000	-6.997334000

----

^{CF3}Catechol-H$_2$ (*S* = 0)

Zero-point correction= 0.112349 (Hartree/Particle)
Thermal correction to Energy= 0.122279
Thermal correction to Enthalpy= 0.123223
Thermal correction to Gibbs Free Energy= 0.076815
Sum of electronic and zero-point Energies= -719.895220
Sum of electronic and thermal Energies= -719.885290
Sum of electronic and thermal Enthalpies= -719.884346
Sum of electronic and thermal Free Energies= -719.930754

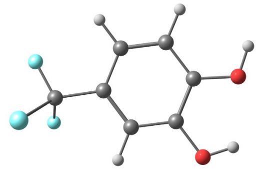

Cartesian Coordinates

C	-2.020812000	0.586532000	0.081732000
C	-0.620174000	0.649124000	0.022166000
C	0.037497000	1.879679000	-0.037482000
C	-0.710387000	3.060696000	-0.032549000
C	-2.099915000	3.004272000	0.030750000
C	-2.761446000	1.761713000	0.086070000
H	-2.541083000	-0.365716000	0.124863000
H	1.119495000	1.931011000	-0.086016000
H	-0.209682000	4.024936000	-0.077656000
O	-4.122664000	1.700193000	0.145754000
H	-4.482262000	2.606064000	0.141702000
O	-2.921803000	4.098601000	0.045918000
H	-2.404821000	4.922601000	0.004135000
C	0.150696000	-0.635355000	-0.013637000
F	1.485462000	-0.460082000	0.152881000
F	-0.248584000	-1.513304000	0.950771000
F	0.000314000	-1.303675000	-1.198307000

[^{CF3}Catechol-H$_2$]$^{\bullet+}$ (*S* = 1/2)

Zero-point correction= 0.112749 (Hartree/Particle)
Thermal correction to Energy= 0.122682
Thermal correction to Enthalpy= 0.123626
Thermal correction to Gibbs Free Energy= 0.076302
Sum of electronic and zero-point Energies= -719.661496
Sum of electronic and thermal Energies= -719.651563
Sum of electronic and thermal Enthalpies= -719.650619
Sum of electronic and thermal Free Energies= -719.697943

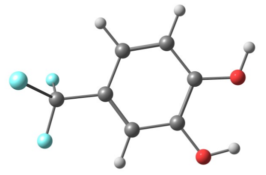

Cartesian Coordinates

C	-2.037233000	0.551847000	0.207449000
C	-0.681276000	0.613872000	0.015205000
C	-0.006357000	1.868897000	-0.180468000
C	-0.699969000	3.062008000	-0.180981000
C	-2.084969000	3.032169000	0.007200000
C	-2.767430000	1.763406000	0.203685000
H	-2.568144000	-0.381357000	0.359945000
H	1.068985000	1.877756000	-0.330544000
H	-0.188085000	4.008554000	-0.326061000
O	-4.070215000	1.722682000	0.376316000
H	-4.479299000	2.616578000	0.356145000
O	-2.892382000	4.082367000	0.025222000
H	-2.419911000	4.931954000	-0.111041000
C	0.154472000	-0.647894000	-0.010451000
F	1.137929000	-0.598323000	0.919562000
F	-0.572094000	-1.755994000	0.225047000
F	0.755809000	-0.801231000	-1.215136000

[^{CF3}Catechol-H]$^{-}$ (S = 0) (No H-bond)

Zero-point correction= 0.098735 (Hartree/Particle)
Thermal correction to Energy= 0.108493
Thermal correction to Enthalpy= 0.109437
Thermal correction to Gibbs Free Energy= 0.063256
Sum of electronic and zero-point Energies= -719.417711
Sum of electronic and thermal Energies= -719.407952
Sum of electronic and thermal Enthalpies= -719.407008
Sum of electronic and thermal Free Energies= -719.453190

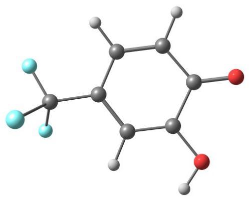

Cartesian Coordinates

C	-2.010449000	0.581061000	0.093224000
C	-0.594036000	0.628337000	0.036113000

C	0.034605000	1.880296000	-0.035209000
C	-0.722234000	3.048021000	-0.036475000
C	-2.159028000	3.064329000	0.026879000
C	-2.762583000	1.739226000	0.089586000
H	-2.516031000	-0.383768000	0.142551000
H	1.118773000	1.943295000	-0.087059000
H	-0.224181000	4.015042000	-0.084427000
O	-4.141484000	1.709233000	0.152040000
H	-4.430269000	0.781482000	0.189810000
O	-2.853582000	4.130240000	0.029573000
C	0.170357000	-0.630357000	-0.015452000
F	1.507662000	-0.471731000	0.197676000
F	-0.249272000	-1.570948000	0.899408000
F	0.082821000	-1.301810000	-1.228842000

[^{CF3}Catechol-H]$^{-}$ (*S* = 0) (H-bond)

Zero-point correction= 0.099283 (Hartree/Particle)
Thermal correction to Energy= 0.108796
Thermal correction to Enthalpy= 0.109740
Thermal correction to Gibbs Free Energy= 0.063997
Sum of electronic and zero-point Energies= -719.431273
Sum of electronic and thermal Energies= -719.421760
Sum of electronic and thermal Enthalpies= -719.420816
Sum of electronic and thermal Free Energies= -719.466559

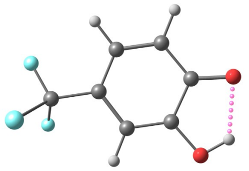

Cartesian Coordinates

C	-2.025130000	0.555879000	0.095642000
C	-0.607689000	0.633023000	0.035177000
C	0.016624000	1.885364000	-0.033813000
C	-0.738141000	3.062546000	-0.034466000
C	-2.158363000	3.035573000	0.027975000
C	-2.761520000	1.718982000	0.093012000
H	-2.529139000	-0.406770000	0.145738000
H	1.100330000	1.948151000	-0.085687000
H	-0.239729000	4.029085000	-0.081498000
O	-4.127837000	1.729450000	0.156780000
H	-4.321638000	2.701353000	0.137913000
O	-2.951603000	4.049727000	0.033856000
C	0.174797000	-0.619685000	-0.017106000
F	1.512349000	-0.442852000	0.177463000
F	-0.220834000	-1.554282000	0.911412000
F	0.075870000	-1.294529000	-1.223496000

[^{CF3}Catecholate]$^{2-}$ (S = 0)

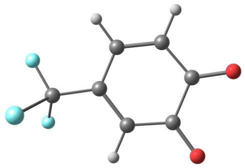

Zero-point correction= 0.084986 (Hartree/Particle)
Thermal correction to Energy= 0.094570
Thermal correction to Enthalpy= 0.095514
Thermal correction to Gibbs Free Energy= 0.049483
Sum of electronic and zero-point Energies= -718.903244
Sum of electronic and thermal Energies= -718.893660
Sum of electronic and thermal Enthalpies= -718.892716
Sum of electronic and thermal Free Energies= -718.938747

Cartesian Coordinates

C	-2.027178000	0.570509000	0.099245000
C	-0.601412000	0.631871000	0.046689000
C	0.031803000	1.876383000	-0.027448000
C	-0.756212000	3.040542000	-0.039492000
C	-2.186558000	3.050315000	0.013645000
C	-2.870403000	1.711540000	0.094446000
H	-2.517596000	-0.402691000	0.151977000
H	1.116327000	1.949594000	-0.075799000
H	-0.265211000	4.015054000	-0.088546000
O	-4.155752000	1.625681000	0.159606000
O	-2.859546000	4.142068000	-0.005711000
C	0.170006000	-0.613430000	-0.014318000
F	1.516039000	-0.463546000	0.195839000
F	-0.232969000	-1.581589000	0.889580000
F	0.095226000	-1.293806000	-1.237574000

[^{CF3}Catechol-H]$^{\bullet}$···H$^{\bullet}$ (S = 1)

Zero-point correction= 0.100325 (Hartree/Particle)
Thermal correction to Energy= 0.111645
Thermal correction to Enthalpy= 0.112589
Thermal correction to Gibbs Free Energy= 0.059432
Sum of electronic and zero-point Energies= -719.774710
Sum of electronic and thermal Energies= -719.763390
Sum of electronic and thermal Enthalpies= -719.762446
Sum of electronic and thermal Free Energies= -719.815603

Cartesian Coordinates

C	0.636290000	0.012320000	-4.351419000
C	-0.479276000	-0.535922000	-3.650596000
C	1.710292000	0.545088000	-3.679967000

H	-1.311448000	-0.948781000	-4.213259000
H	2.567180000	0.966287000	-4.194503000
C	-0.520116000	-0.551324000	-2.262487000
C	1.716651000	0.553349000	-2.237810000
H	-1.370496000	-0.970124000	-1.733217000
C	0.554531000	-0.018214000	-1.552923000
O	0.576352000	-0.002737000	-0.219957000
H	1.429039000	0.419506000	0.039454000
O	2.652950000	1.018296000	-1.532527000
C	0.598125000	-0.008661000	-5.855152000
F	1.710939000	0.507471000	-6.423242000
F	0.458832000	-1.273853000	-6.341385000
F	-0.458230000	0.698814000	-6.346043000
H	9.353927000	6.071436000	3.948828000

[^{CF3}Quinone] ···H$^{•}$ (S = 1/2)

Zero-point correction= 0.088751 (Hartree/Particle)
Thermal correction to Energy= 0.100129
Thermal correction to Enthalpy= 0.101073
Thermal correction to Gibbs Free Energy= 0.047634
Sum of electronic and zero-point Energies= -719.161461
Sum of electronic and thermal Energies= -719.150083
Sum of electronic and thermal Enthalpies= -719.149139
Sum of electronic and thermal Free Energies= -719.202578

Cartesian Coordinates

C	0.701741000	0.023615000	-4.315090000
C	-0.535223000	-0.626526000	-3.865228000
C	1.553589000	0.640789000	-3.469664000
H	-1.163738000	-1.100010000	-4.614513000
H	2.467611000	1.122390000	-3.801711000
C	-0.899941000	-0.645953000	-2.565845000
C	1.255483000	0.674272000	-2.025919000
H	-1.816525000	-1.127283000	-2.237161000
C	-0.062255000	-0.009948000	-1.542346000
O	-0.348334000	0.005847000	-0.354721000
O	1.998541000	1.209226000	-1.219463000
C	0.988320000	-0.032255000	-5.797072000
F	2.129982000	0.595004000	-6.144312000
F	1.089565000	-1.317475000	-6.227541000
F	-0.016409000	0.537752000	-6.512361000

H 2.266420000 2.281302000 9.053471000

[^{CF3}Catechol-H]$^{\bullet}$ (*S* = 1/2)

Zero-point correction= 0.100203 (Hartree/Particle)
Thermal correction to Energy= 0.109747
Thermal correction to Enthalpy= 0.110691
Thermal correction to Gibbs Free Energy= 0.064228
Sum of electronic and zero-point Energies= -719.272970
Sum of electronic and thermal Energies= -719.263426
Sum of electronic and thermal Enthalpies= -719.262482
Sum of electronic and thermal Free Energies= -719.308945

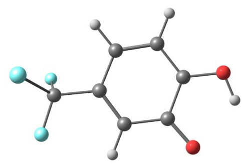

Cartesian Coordinates

C	0.631657000	0.010432000	-4.354104000
C	-0.485393000	-0.521736000	-3.643292000
C	1.718244000	0.529763000	-3.692429000
H	-1.327631000	-0.924381000	-4.198392000
H	2.576574000	0.938431000	-4.214660000
C	-0.515108000	-0.534058000	-2.254834000
C	1.736778000	0.539761000	-2.250405000
H	-1.366857000	-0.939913000	-1.717715000
C	0.572491000	-0.014462000	-1.555004000
O	0.605540000	0.003035000	-0.222306000
H	1.466196000	0.413745000	0.029276000
O	2.685561000	0.991928000	-1.553593000
C	0.580859000	-0.012774000	-5.857393000
F	1.696054000	0.486594000	-6.435749000
F	0.420001000	-1.276914000	-6.339574000
F	-0.469523000	0.708299000	-6.341226000

---

^{CF3}Quinone (*S* = 0)

Zero-point correction= 0.088573 (Hartree/Particle)
Thermal correction to Energy= 0.098187
Thermal correction to Enthalpy= 0.099131
Thermal correction to Gibbs Free Energy= 0.052928
Sum of electronic and zero-point Energies= -718.659729
Sum of electronic and thermal Energies= -718.650115
Sum of electronic and thermal Enthalpies= -718.649171
Sum of electronic and thermal Free Energies= -718.695374

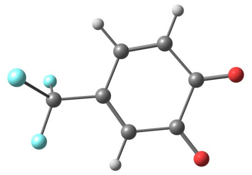

Cartesian Coordinates

C	0.634626000	0.027762000	-4.374634000
C	-0.517514000	-0.526118000	-3.652404000
C	1.712912000	0.543290000	-3.747943000
H	-1.344459000	-0.919783000	-4.237409000
H	2.564006000	0.953767000	-4.281700000
C	-0.568097000	-0.554802000	-2.303872000
C	1.762245000	0.562730000	-2.274328000
H	-1.421777000	-0.965695000	-1.772666000
C	0.541573000	-0.027852000	-1.500916000
O	0.548586000	-0.032692000	-0.279256000
O	2.712367000	1.019157000	-1.659914000
C	0.559299000	-0.003522000	-5.882859000
F	1.655515000	0.502033000	-6.482883000
F	0.402757000	-1.274080000	-6.338710000
F	-0.508795000	0.704651000	-6.334875000

---

^{Cl2}Catechol-H$_2$ (*S* = 0)

Zero-point correction= 0.089161 (Hartree/Particle)
Thermal correction to Energy= 0.098026
Thermal correction to Enthalpy= 0.098971
Thermal correction to Gibbs Free Energy= 0.054745
Sum of electronic and zero-point Energies= -1302.000532
Sum of electronic and thermal Energies= -1301.991666
Sum of electronic and thermal Enthalpies= -1301.990722
Sum of electronic and thermal Free Energies= -1302.034948

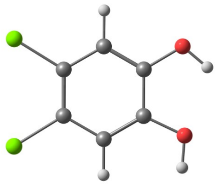

Cartesian Coordinates

C	0.621840000	0.002116000	-4.344080000
C	-0.546574000	-0.336474000	-3.656770000
C	1.775250000	0.349335000	-3.630798000
H	2.682754000	0.612144000	-4.166963000
C	-0.564607000	-0.328632000	-2.258305000
C	1.754879000	0.356376000	-2.241230000
H	-1.468798000	-0.590606000	-1.718546000
C	0.579562000	0.015956000	-1.547582000
O	0.545649000	0.018860000	-0.185875000
H	1.421419000	0.279840000	0.153716000
O	2.826009000	0.679941000	-1.450985000
H	3.604907000	0.904551000	-1.990156000
Cl	-2.016724000	-0.778640000	-4.506201000
Cl	0.694469000	0.007490000	-6.096954000

[^{Cl2}Catechol-H$_2$]$^{\bullet+}$ (*S* = 1/2)

Zero-point correction=	0.089477 (Hartree/Particle)
Thermal correction to Energy=	0.098230
Thermal correction to Enthalpy=	0.099174
Thermal correction to Gibbs Free Energy=	0.054196
Sum of electronic and zero-point Energies=	-1301.772754
Sum of electronic and thermal Energies=	-1301.764000
Sum of electronic and thermal Enthalpies=	-1301.763056
Sum of electronic and thermal Free Energies=	-1301.808035

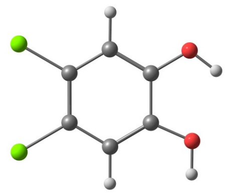

Cartesian Coordinates

C	0.648430000	0.008120000	-4.342866000
C	-0.565741000	-0.340853000	-3.628870000
C	1.801419000	0.355675000	-3.654141000
H	2.706334000	0.616670000	-4.193159000
C	-0.592198000	-0.336798000	-2.249950000
C	1.778230000	0.363856000	-2.261971000
H	-1.490108000	-0.598129000	-1.699961000
C	0.567515000	0.011645000	-1.545996000
O	0.544828000	0.016150000	-0.226705000
H	1.411417000	0.273276000	0.156530000
O	2.799295000	0.675206000	-1.470881000
H	3.612043000	0.912545000	-1.965989000
Cl	-1.986497000	-0.762700000	-4.499048000
Cl	0.675068000	-0.002406000	-6.057721000

[^{Cl2}Catechol-H]$^-$ (*S* = 0) (No H-bond)

Zero-point correction=	0.075537 (Hartree/Particle)
Thermal correction to Energy=	0.084331
Thermal correction to Enthalpy=	0.085275
Thermal correction to Gibbs Free Energy=	0.040965
Sum of electronic and zero-point Energies=	-1301.522808
Sum of electronic and thermal Energies=	-1301.514014
Sum of electronic and thermal Enthalpies=	-1301.513070
Sum of electronic and thermal Free Energies=	-1301.557380

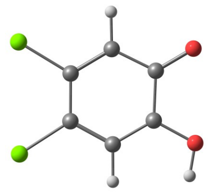

Cartesian Coordinates

C	0.623994000	0.001796000	-4.344402000
C	-0.538422000	-0.334064000	-3.654963000
C	1.767868000	0.346202000	-3.597244000
H	2.681396000	0.610791000	-4.127769000
C	-0.571487000	-0.329970000	-2.259334000

C	1.748486000	0.354906000	-2.211827000
H	-1.490939000	-0.595928000	-1.745315000
C	0.562012000	0.010707000	-1.447725000
O	0.536782000	0.013501000	-0.172643000
O	2.866739000	0.693835000	-1.478366000
H	3.592248000	0.902091000	-2.091360000
Cl	-2.020401000	-0.778520000	-4.528245000
Cl	0.726755000	0.016568000	-6.109281000

[^{Cl2}Catechol-H]$^{-}$ (*S* = 0) (H-bond)

Zero-point correction= 0.076126 (Hartree/Particle)
Thermal correction to Energy= 0.084639
Thermal correction to Enthalpy= 0.085583
Thermal correction to Gibbs Free Energy= 0.041845
Sum of electronic and zero-point Energies= -1301.536650
Sum of electronic and thermal Energies= -1301.528137
Sum of electronic and thermal Enthalpies= -1301.527193
Sum of electronic and thermal Free Energies= -1301.570931

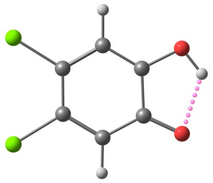

Cartesian Coordinates

C	0.622246000	0.002432000	-4.315956000
C	-0.558032000	-0.339982000	-3.661897000
C	1.779085000	0.349515000	-3.600655000
H	2.686205000	0.612007000	-4.137738000
C	-0.592879000	-0.336314000	-2.249824000
C	1.787760000	0.364870000	-2.183262000
H	-1.508320000	-0.601145000	-1.727875000
C	0.542522000	0.004971000	-1.543893000
O	0.581768000	0.030400000	-0.179054000
H	1.520502000	0.307643000	-0.014304000
O	2.783857000	0.664578000	-1.418756000
Cl	-2.031507000	-0.784621000	-4.532509000
Cl	0.712863000	0.015280000	-6.088031000

[^{Cl2}Catecholate]$^{2-}$ (*S* = 0)

Zero-point correction= 0.061732 (Hartree/Particle)
Thermal correction to Energy= 0.070494

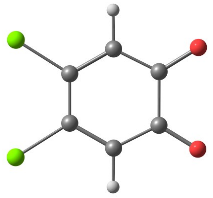

Thermal correction to Enthalpy= 0.071438
Thermal correction to Gibbs Free Energy= 0.027071
Sum of electronic and zero-point Energies= -1301.011508
Sum of electronic and thermal Energies= -1301.002745
Sum of electronic and thermal Enthalpies= -1301.001801
Sum of electronic and thermal Free Energies= -1301.046169

Cartesian Coordinates

C	0.611857000	-0.002757000	-4.342941000
C	-0.548646000	-0.338738000	-3.671635000
C	1.769033000	0.341510000	-3.606429000
H	2.676616000	0.603687000	-4.147989000
C	-0.565917000	-0.323856000	-2.257719000
C	1.821741000	0.384143000	-2.182319000
H	-1.486624000	-0.591612000	-1.742009000
C	0.564139000	0.003589000	-1.452224000
O	0.511754000	-0.014446000	-0.166395000
O	2.890749000	0.721613000	-1.550216000
Cl	-2.055939000	-0.774463000	-4.532610000
Cl	0.712235000	0.000652000	-6.130053000

[^{Cl2}Catechol-H]$^{\bullet}$···H$^{\bullet}$ (*S* = 1)

Zero-point correction= 0.077108 (Hartree/Particle)
Thermal correction to Energy= 0.087332
Thermal correction to Enthalpy= 0.088276
Thermal correction to Gibbs Free Energy= 0.036846
Sum of electronic and zero-point Energies= -1301.882359
Sum of electronic and thermal Energies= -1301.872136
Sum of electronic and thermal Enthalpies= -1301.871192
Sum of electronic and thermal Free Energies= -1301.922621

Cartesian Coordinates

C	0.600820000	-0.001420000	-4.359255000
C	-0.574448000	-0.382695000	-3.633038000
C	1.736253000	0.387578000	-3.694862000
H	2.632504000	0.677789000	-4.233179000
C	-0.597732000	-0.369489000	-2.238455000
C	1.764903000	0.419207000	-2.255284000
H	-1.492832000	-0.659551000	-1.698204000
C	0.542781000	0.021569000	-1.551626000
O	0.577536000	0.053159000	-0.218252000
H	1.482535000	0.358951000	0.026851000
O	2.761438000	0.762862000	-1.567523000

Cl	-2.011977000	-0.874271000	-4.474016000
Cl	0.601840000	-0.027555000	-6.108292000
H	9.033342000	3.073336000	5.870550000

[^{Cl2}Quinone] ···H$^{•}$ (S = 1/2)

Zero-point correction= 0.065491 (Hartree/Particle)
Thermal correction to Energy= 0.075689
Thermal correction to Enthalpy= 0.076633
Thermal correction to Gibbs Free Energy= 0.025339
Sum of electronic and zero-point Energies= -1301.270769
Sum of electronic and thermal Energies= -1301.260571
Sum of electronic and thermal Enthalpies= -1301.259627
Sum of electronic and thermal Free Energies= -1301.310921

Cartesian Coordinates

C	0.588186000	0.073422000	-4.389511000
C	-0.558145000	-0.463236000	-3.618251000
C	1.712957000	0.505961000	-3.779613000
H	2.551449000	0.898149000	-4.346033000
C	-0.532618000	-0.539580000	-2.270034000
C	1.844708000	0.461692000	-2.320079000
H	-1.373549000	-0.932076000	-1.707414000
C	0.639544000	-0.092972000	-1.511081000
O	0.691715000	-0.144951000	-0.291862000
O	2.851247000	0.837989000	-1.739453000
Cl	0.473212000	0.143719000	-6.123475000
Cl	-1.967913000	-1.006159000	-4.479908000
H	6.260664000	2.778579000	7.458916000

[^{Cl2}Catechol-H]$^{•}$ (S = 1/2)

Zero-point correction= 0.077022 (Hartree/Particle)
Thermal correction to Energy= 0.085440
Thermal correction to Enthalpy= 0.086384
Thermal correction to Gibbs Free Energy= 0.042152
Sum of electronic and zero-point Energies= -1301.380580
Sum of electronic and thermal Energies= -1301.372162
Sum of electronic and thermal Enthalpies= -1301.371218
Sum of electronic and thermal Free Energies= -1301.415449

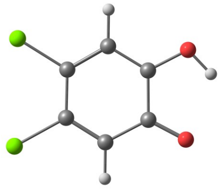

Cartesian Coordinates

C	0.590629000	0.014739000	-4.360014000
C	-0.586723000	-0.350512000	-3.628967000
C	1.739478000	0.370608000	-3.700173000
H	2.637411000	0.648571000	-4.242063000
C	-0.598324000	-0.354971000	-2.234193000
C	1.780759000	0.382324000	-2.260599000
H	-1.494991000	-0.633073000	-1.690274000
C	0.555978000	0.002224000	-1.551931000
O	0.602107000	0.015633000	-0.218552000
H	1.515672000	0.297851000	0.022992000
O	2.790319000	0.694697000	-1.576951000
Cl	-2.041132000	-0.800684000	-4.464049000
Cl	0.577399000	0.010729000	-6.109266000

---

^{Cl2}Quinone (*S* = 0)

Zero-point correction= 0.065285 (Hartree/Particle)
Thermal correction to Energy= 0.073716
Thermal correction to Enthalpy= 0.074660
Thermal correction to Gibbs Free Energy= 0.030708
Sum of electronic and zero-point Energies= -1300.769076
Sum of electronic and thermal Energies= -1300.760645
Sum of electronic and thermal Enthalpies= -1300.759700
Sum of electronic and thermal Free Energies= -1300.803653

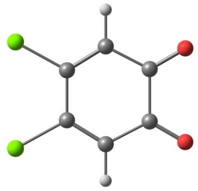

Cartesian Coordinates

C	0.636080000	0.028598000	-4.389085000
C	-0.524652000	-0.529286000	-3.655594000
C	1.706285000	0.537943000	-3.741362000
H	2.554781000	0.946153000	-4.281128000
C	-0.565600000	-0.553638000	-2.305820000
C	1.760606000	0.561702000	-2.276253000
H	-1.415847000	-0.963158000	-1.769932000
C	0.545006000	-0.026039000	-1.507674000
O	0.539033000	-0.036515000	-0.286300000
O	2.710754000	1.020409000	-1.660915000
Cl	0.610534000	0.023963000	-6.127958000
Cl	-1.863392000	-1.166746000	-4.564669000

---

^{Cl}Catechol-H_2 (*S* = 0)

Zero-point correction= 0.098688 (Hartree/Particle)
Thermal correction to Energy= 0.106321

Thermal correction to Enthalpy= 0.107265
Thermal correction to Gibbs Free Energy= 0.066298
Sum of electronic and zero-point Energies= -842.368725
Sum of electronic and thermal Energies= -842.361093
Sum of electronic and thermal Enthalpies= -842.360149
Sum of electronic and thermal Free Energies= -842.401116

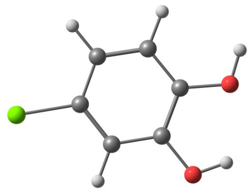

Cartesian Coordinates

C	0.591421000	-0.006516000	-4.371942000
C	-0.561915000	-0.349100000	-3.663571000
C	1.739847000	0.336439000	-3.661093000
C	-0.595943000	-0.357682000	-2.271640000
C	1.724856000	0.333811000	-2.255013000
H	-1.500227000	-0.626218000	-1.735842000
C	0.564716000	-0.011204000	-1.568415000
H	0.559242000	-0.012068000	-0.480531000
H	0.611577000	-0.001721000	-5.456918000
O	2.911134000	0.688366000	-1.659749000
H	2.831346000	0.665619000	-0.690140000
O	2.871728000	0.672713000	-4.344535000
H	3.575610000	0.882513000	-3.703646000
Cl	-2.016490000	-0.783166000	-4.570043000

[^{Cl}Catechol-H_2]$^{\bullet+}$ (*S* = 1/2)

Zero-point correction= 0.099008 (Hartree/Particle)
Thermal correction to Energy= 0.106631
Thermal correction to Enthalpy= 0.107575
Thermal correction to Gibbs Free Energy= 0.065591
Sum of electronic and zero-point Energies= -842.144315
Sum of electronic and thermal Energies= -842.136693
Sum of electronic and thermal Enthalpies= -842.135749
Sum of electronic and thermal Free Energies= -842.177733

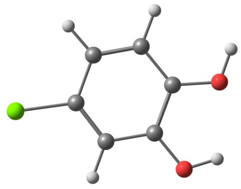

Cartesian Coordinates

C	0.595410000	-0.007042000	-4.404112000
C	-0.540991000	-0.345106000	-3.691562000
C	1.740086000	0.340048000	-3.683711000
C	-0.576364000	-0.346331000	-2.253884000
C	1.718956000	0.331652000	-2.230243000
H	-1.494948000	-0.616254000	-1.743626000
C	0.546997000	-0.011334000	-1.534352000
H	0.536875000	-0.012336000	-0.447954000

H	0.619830000	-0.004483000	-5.488208000
O	2.875019000	0.664848000	-1.673833000
H	2.844529000	0.655211000	-0.692823000
O	2.842914000	0.674256000	-4.333650000
H	3.578963000	0.892578000	-3.723099000
Cl	-1.980375000	-0.783921000	-4.532020000

[^{Cl}Catechol-H]$^{-}$ (S = 0) (No H-bond)

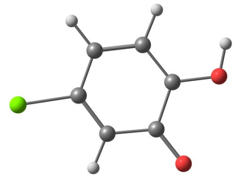

Zero-point correction= 0.084858 (Hartree/Particle)
Thermal correction to Energy= 0.092385
Thermal correction to Enthalpy= 0.093329
Thermal correction to Gibbs Free Energy= 0.052407
Sum of electronic and zero-point Energies= -841.887205
Sum of electronic and thermal Energies= -841.879678
Sum of electronic and thermal Enthalpies= -841.878734
Sum of electronic and thermal Free Energies= -841.919657

Cartesian Coordinates

C	0.585172000	-0.004715000	-4.375167000
C	-0.555700000	-0.344497000	-3.652784000
C	1.817871000	0.350123000	-3.724686000
C	-0.598015000	-0.365180000	-2.263543000
C	1.746313000	0.343673000	-2.274557000
H	-1.493353000	-0.638860000	-1.715843000
C	0.592478000	-0.008461000	-1.591132000
H	0.599076000	-0.009768000	-0.499884000
H	0.573819000	-0.001586000	-5.461800000
O	2.905070000	0.705977000	-1.609530000
H	2.738268000	0.655476000	-0.653093000
O	2.883095000	0.647047000	-4.362870000
Cl	-2.043024000	-0.774099000	-4.560931000

[^{Cl}Catechol-H]$^{-}$ (S = 0) (H-bond)

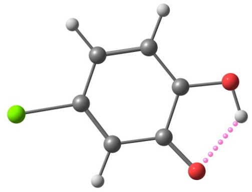

Zero-point correction= 0.085457 (Hartree/Particle)
Thermal correction to Energy= 0.092709
Thermal correction to Enthalpy= 0.093653
Thermal correction to Gibbs Free Energy= 0.053283
Sum of electronic and zero-point Energies= -841.901116
Sum of electronic and thermal Energies= -841.893864
Sum of electronic and thermal Enthalpies= -841.892920
Sum of electronic and thermal Free Energies= -841.933290

Cartesian Coordinates

C	0.574487000	-0.016183000	-4.329837000
C	-0.605544000	-0.342748000	-3.673426000
C	1.757269000	0.330886000	-3.666473000
H	-1.504700000	-0.607530000	-4.220029000
H	2.657214000	0.579307000	-4.222415000
C	-0.597213000	-0.318665000	-2.257671000
C	1.789976000	0.360488000	-2.245977000
H	-1.501514000	-0.568644000	-1.705729000
C	0.555599000	0.019398000	-1.576339000
O	0.628521000	0.060363000	-0.208653000
H	1.573012000	0.329865000	-0.070708000
O	2.807734000	0.659810000	-1.507013000
Cl	0.590105000	-0.039372000	-6.121892000

[^{Cl}Catecholate]$^{2-}$ (*S* = 0)

Zero-point correction= 0.071027 (Hartree/Particle)
Thermal correction to Energy= 0.078473
Thermal correction to Enthalpy= 0.079418
Thermal correction to Gibbs Free Energy= 0.038508
Sum of electronic and zero-point Energies= -841.369796
Sum of electronic and thermal Energies= -841.362350
Sum of electronic and thermal Enthalpies= -841.361406
Sum of electronic and thermal Free Energies= -841.402316

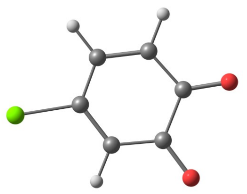

Cartesian Coordinates

C	0.547375000	-0.003796000	-4.370705000
C	-0.614308000	-0.343949000	-3.705417000
C	1.730443000	0.340081000	-3.688576000
H	-1.523746000	-0.612602000	-4.238545000
H	2.628221000	0.601398000	-4.247475000
C	-0.572290000	-0.333282000	-2.282725000
C	1.816770000	0.354627000	-2.261562000
H	-1.480215000	-0.597846000	-1.735535000
C	0.570156000	-0.000937000	-1.501672000
O	0.554519000	-0.002130000	-0.208704000
O	2.912116000	0.660369000	-1.656200000
Cl	0.567759000	0.002027000	-6.183959000

[^{Cl}Catechol-H]$^{\bullet}$ ···H$^{\bullet}$ (*S* = 1)

Zero-point correction= 0.086710 (Hartree/Particle)
Thermal correction to Energy= 0.095703
Thermal correction to Enthalpy= 0.096647
Thermal correction to Gibbs Free Energy= 0.048773
Sum of electronic and zero-point Energies= -842.252132
Sum of electronic and thermal Energies= -842.243139
Sum of electronic and thermal Enthalpies= -842.242195
Sum of electronic and thermal Free Energies= -842.290069

Cartesian Coordinates

C	0.617346000	-0.000047000	-4.354984000
C	-0.540619000	-0.399561000	-3.632924000
C	1.735447000	0.413250000	-3.672718000
H	2.633281000	0.723636000	-4.199098000
C	-0.605696000	-0.395717000	-2.239505000
C	1.746331000	0.445766000	-2.231735000
H	-1.500856000	-0.704980000	-1.709993000
C	0.521860000	0.020531000	-1.543992000
H	0.604983000	-0.025969000	-5.439926000
O	0.545556000	0.053804000	-0.207972000
H	1.443167000	0.380211000	0.038354000
O	2.723586000	0.810257000	-1.522762000
Cl	-1.941520000	-0.917914000	-4.539973000
H	10.600753000	3.064666000	4.210290000

[^{Cl}Quinone] ⋯H$^{•}$ (*S* = 1/2)

Zero-point correction= 0.074993 (Hartree/Particle)
Thermal correction to Energy= 0.084021
Thermal correction to Enthalpy= 0.084965
Thermal correction to Gibbs Free Energy= 0.037375
Sum of electronic and zero-point Energies= -841.641125
Sum of electronic and thermal Energies= -841.632097
Sum of electronic and thermal Enthalpies= -841.631153
Sum of electronic and thermal Free Energies= -841.678743

Cartesian Coordinates

C	0.617238000	0.118303000	-4.344048000
C	-0.551536000	-0.416690000	-3.639243000
C	1.747097000	0.525333000	-3.724893000
H	-1.411763000	-0.712203000	-4.232283000
H	2.601605000	0.920354000	-4.264873000
C	-0.561205000	-0.538687000	-2.295463000

C	1.837308000	0.437312000	-2.264964000
H	-1.422857000	-0.934391000	-1.765558000
C	0.605499000	-0.138085000	-1.495936000
O	0.643753000	-0.237643000	-0.279210000
O	2.828175000	0.791427000	-1.641845000
Cl	0.481760000	0.207939000	-6.084828000
H	3.950290000	7.224327000	5.931705000

[^{Cl}Catechol-H]$^{\bullet}$ (*S* = 1/2)

Zero-point correction= 0.086611 (Hartree/Particle)
Thermal correction to Energy= 0.093810
Thermal correction to Enthalpy= 0.094755
Thermal correction to Gibbs Free Energy= 0.053788
Sum of electronic and zero-point Energies= -841.750370
Sum of electronic and thermal Energies= -841.743171
Sum of electronic and thermal Enthalpies= -841.742226
Sum of electronic and thermal Free Energies= -841.783193

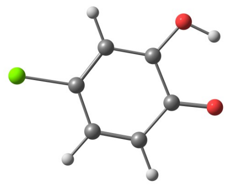

Cartesian Coordinates

C	0.587015000	0.013434000	-4.360319000
C	-0.571065000	-0.345783000	-3.617652000
C	1.736531000	0.369393000	-3.698286000
H	2.635321000	0.647613000	-4.240815000
C	-0.605546000	-0.357411000	-2.223234000
C	1.780331000	0.382189000	-2.257577000
H	-1.501475000	-0.635135000	-1.677736000
C	0.553687000	0.001414000	-1.548075000
H	0.549984000	0.002154000	-5.444911000
O	0.607314000	0.017208000	-0.212556000
H	1.523588000	0.300566000	0.017779000
O	2.787825000	0.694283000	-1.566159000
Cl	-2.012351000	-0.791555000	-4.499573000

---

^{Cl}Quinone (*S* = 0)

Zero-point correction= 0.074828 (Hartree/Particle)
Thermal correction to Energy= 0.082111
Thermal correction to Enthalpy= 0.083055
Thermal correction to Gibbs Free Energy= 0.042256
Sum of electronic and zero-point Energies= -841.139405
Sum of electronic and thermal Energies= -841.132123
Sum of electronic and thermal Enthalpies= -841.131178

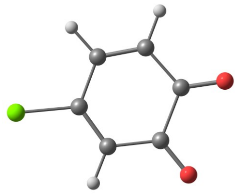

Sum of electronic and thermal Free Energies= -841.171977

Cartesian Coordinates

C	0.642819000	0.031191000	-4.364924000
C	-0.516202000	-0.524459000	-3.660085000
C	1.724961000	0.546951000	-3.741437000
H	-1.335675000	-0.912964000	-4.257174000
H	2.573877000	0.954559000	-4.281133000
C	-0.566314000	-0.554069000	-2.312052000
C	1.767577000	0.566610000	-2.276580000
H	-1.420839000	-0.965499000	-1.782544000
C	0.544934000	-0.027292000	-1.507325000
O	0.545119000	-0.037990000	-0.286000000
O	2.711782000	1.025309000	-1.649258000
Cl	0.560549000	-0.003659000	-6.110943000

^{CH3}Catechol-H$_2$ (S = 0)

Zero-point correction= 0.135407 (Hartree/Particle)
Thermal correction to Energy= 0.143439
Thermal correction to Enthalpy= 0.144384
Thermal correction to Gibbs Free Energy= 0.103355
Sum of electronic and zero-point Energies= -422.033342
Sum of electronic and thermal Energies= -422.025309
Sum of electronic and thermal Enthalpies= -422.024365
Sum of electronic and thermal Free Energies= -422.065394

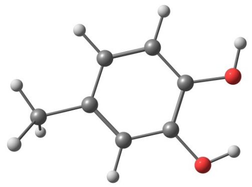

Cartesian Coordinates

C	0.560082000	0.079345000	-4.350174000
C	-0.591032000	-0.363027000	-3.675084000
C	1.714664000	0.418919000	-3.650832000
C	-0.550135000	-0.454901000	-2.278388000
C	1.738772000	0.320987000	-2.249065000
H	-1.427031000	-0.794118000	-1.732291000
C	0.608866000	-0.115108000	-1.565906000
H	0.629647000	-0.190685000	-0.480135000
H	0.566622000	0.163706000	-5.434766000
O	2.928833000	0.680795000	-1.653823000
H	2.868357000	0.586148000	-0.687616000
O	2.820878000	0.846752000	-4.335309000
H	3.528389000	1.039437000	-3.693804000
C	-1.835913000	-0.726306000	-4.452293000
H	-1.639533000	-1.532965000	-5.170765000

H	-2.636302000	-1.061214000	-3.783653000
H	-2.214694000	0.129681000	-5.026289000

[^{CH3}Catechol-H$_2$]$^{•+}$ (*S* = 1/2)

Zero-point correction= 0.135609 (Hartree/Particle)
Thermal correction to Energy= 0.143604
Thermal correction to Enthalpy= 0.144548
Thermal correction to Gibbs Free Energy= 0.102765
Sum of electronic and zero-point Energies= -421.820078
Sum of electronic and thermal Energies= -421.812083
Sum of electronic and thermal Enthalpies= -421.811139
Sum of electronic and thermal Free Energies= -421.852922

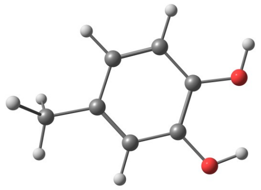

Cartesian Coordinates

C	0.604649000	-0.023896000	-4.400932000
C	-0.561428000	-0.349511000	-3.723347000
C	1.751150000	0.318984000	-3.680842000
C	-0.570421000	-0.330274000	-2.275666000
C	1.719722000	0.341549000	-2.228469000
H	-1.491296000	-0.592404000	-1.762483000
C	0.544016000	0.007814000	-1.540492000
H	0.526678000	0.019060000	-0.454101000
H	0.647061000	-0.028978000	-5.485317000
O	2.872513000	0.692647000	-1.666660000
H	2.829670000	0.696661000	-0.687093000
O	2.868804000	0.624536000	-4.324882000
H	3.596080000	0.848765000	-3.706929000
C	-1.814487000	-0.722874000	-4.444060000
H	-1.694096000	-0.670420000	-5.528554000
H	-2.119712000	-1.742406000	-4.168441000
H	-2.638434000	-0.061805000	-4.141926000

[^{CH3}Catechol-H]$^{-}$ (*S* = 0) (No H-bond)

Zero-point correction= 0.121642 (Hartree/Particle)
Thermal correction to Energy= 0.129479
Thermal correction to Enthalpy= 0.130423
Thermal correction to Gibbs Free Energy= 0.089633
Sum of electronic and zero-point Energies= -421.543625
Sum of electronic and thermal Energies= -421.535788
Sum of electronic and thermal Enthalpies= -421.534843
Sum of electronic and thermal Free Energies= -421.575634

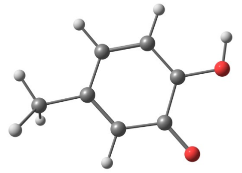

Cartesian Coordinates

C	0.565813000	-0.021222000	-4.362322000
C	-0.566359000	-0.440632000	-3.653479000
C	1.695985000	0.410364000	-3.647303000
H	-1.458506000	-0.780956000	-4.175750000
H	2.581205000	0.739939000	-4.194090000
C	-0.540213000	-0.418974000	-2.241584000
C	1.782763000	0.458737000	-2.216954000
H	-1.420411000	-0.746333000	-1.683306000
C	0.579275000	0.007825000	-1.543263000
O	0.604013000	0.026859000	-0.155497000
H	-0.254240000	-0.296036000	0.166648000
O	2.821397000	0.861441000	-1.579591000
C	0.581523000	-0.031169000	-5.878519000
H	1.371597000	-0.685491000	-6.273416000
H	-0.373948000	-0.384871000	-6.285269000
H	0.765900000	0.971057000	-6.290548000

[^{CH3}Catechol-H]$^{-}$ (S = 0) (H-bond)

Zero-point correction= 0.122174 (Hartree/Particle)
Thermal correction to Energy= 0.129764
Thermal correction to Enthalpy= 0.130708
Thermal correction to Gibbs Free Energy= 0.090386
Sum of electronic and zero-point Energies= -421.558109
Sum of electronic and thermal Energies= -421.550519
Sum of electronic and thermal Enthalpies= -421.549575
Sum of electronic and thermal Free Energies= -421.589897

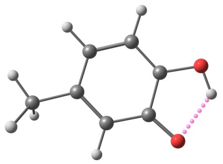

Cartesian Coordinates

C	0.568766000	-0.024174000	-4.367788000
C	-0.564326000	-0.440003000	-3.660350000
C	1.710294000	0.408091000	-3.657720000
H	-1.450746000	-0.776712000	-4.195256000
H	2.595695000	0.733246000	-4.205945000
C	-0.566299000	-0.424614000	-2.243985000
C	1.754880000	0.442239000	-2.241875000
H	-1.446015000	-0.748511000	-1.688780000
C	0.557751000	0.002351000	-1.564475000
O	0.638670000	0.047941000	-0.193900000
H	1.561474000	0.394278000	-0.072461000
O	2.750004000	0.823784000	-1.498664000
C	0.582842000	-0.028945000	-5.883582000

H	1.390604000	-0.659037000	-6.282194000
H	-0.362957000	-0.407262000	-6.290393000
H	0.738479000	0.979720000	-6.292548000

[^{CH3}Catecholate]$^{2-}$ (*S* = 0)

Zero-point correction= 0.107922 (Hartree/Particle)
Thermal correction to Energy= 0.115549
Thermal correction to Enthalpy= 0.116493
Thermal correction to Gibbs Free Energy= 0.075986
Sum of electronic and zero-point Energies= -421.020141
Sum of electronic and thermal Energies= -421.012514
Sum of electronic and thermal Enthalpies= -421.011569
Sum of electronic and thermal Free Energies= -421.052076

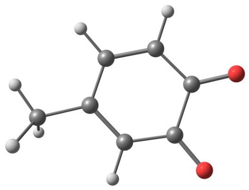

Cartesian Coordinates

C	0.543584000	-0.027458000	-4.411610000
C	-0.570147000	-0.445704000	-3.687939000
C	1.681892000	0.407468000	-3.678242000
H	-1.467264000	-0.792686000	-4.206721000
H	2.567127000	0.741512000	-4.230444000
C	-0.543164000	-0.422893000	-2.265979000
C	1.779913000	0.448199000	-2.255923000
H	-1.425922000	-0.757665000	-1.712915000
C	0.568200000	0.002512000	-1.487315000
O	0.548389000	0.008589000	-0.190975000
O	2.852310000	0.847796000	-1.651190000
C	0.561246000	-0.026852000	-5.927074000
H	1.376526000	-0.643833000	-6.338983000
H	-0.379903000	-0.419635000	-6.336334000
H	0.700499000	0.982906000	-6.347607000

[^{CH3}Catechol-H]$^{\bullet}\cdots$H$^{\bullet}$ (*S* = 1)

Zero-point correction= 0.123529 (Hartree/Particle)
Thermal correction to Energy= 0.132889
Thermal correction to Enthalpy= 0.133834
Thermal correction to Gibbs Free Energy= 0.086349
Sum of electronic and zero-point Energies= -421.920091
Sum of electronic and thermal Energies= -421.910731
Sum of electronic and thermal Enthalpies= -421.909787
Sum of electronic and thermal Free Energies= -421.957272

Cartesian Coordinates

C	0.538219000	0.116829000	-4.336281000
C	-0.606991000	-0.366623000	-3.623102000
C	1.681131000	0.513485000	-3.688119000
H	2.548156000	0.879514000	-4.231300000
C	-0.570876000	-0.439967000	-2.225530000
C	1.759038000	0.452982000	-2.250603000
H	-1.429463000	-0.803619000	-1.667175000
C	0.574882000	-0.043967000	-1.546553000
H	0.487246000	0.165548000	-5.421698000
O	0.657561000	-0.096228000	-0.209640000
H	1.559472000	0.235115000	0.012717000
O	2.761110000	0.792698000	-1.558415000
C	-1.828417000	-0.787818000	-4.389968000
H	-1.586550000	-1.594744000	-5.095117000
H	-2.623793000	-1.137966000	-3.725403000
H	-2.219694000	0.046239000	-4.988437000
H	9.538174000	2.258868000	5.647260000

[^{CH3}Quinone] ···H$^{•}$ (S = 1/2)

Zero-point correction= 0.111906 (Hartree/Particle)
Thermal correction to Energy= 0.121249
Thermal correction to Enthalpy= 0.122193
Thermal correction to Gibbs Free Energy= 0.075095
Sum of electronic and zero-point Energies= -421.311901
Sum of electronic and thermal Energies= -421.302558
Sum of electronic and thermal Enthalpies= -421.301614
Sum of electronic and thermal Free Energies= -421.348712

Cartesian Coordinates

C	0.672460000	0.051323000	-4.410435000
C	-0.504380000	-0.414284000	-3.652273000
C	1.789984000	0.463997000	-3.758195000
H	-1.369405000	-0.731170000	-4.231224000
H	2.667436000	0.810230000	-4.299009000
C	-0.547104000	-0.459716000	-2.303463000
C	1.868433000	0.460085000	-2.300180000
H	-1.424950000	-0.805266000	-1.763914000
C	0.612415000	-0.033579000	-1.511449000
O	0.629260000	-0.051113000	-0.288275000
O	2.861627000	0.821389000	-1.678889000
C	0.566804000	0.042908000	-5.906268000
H	1.482404000	0.406738000	-6.380591000

H	0.360666000	-0.973499000	-6.269436000
H	-0.274168000	0.669356000	-6.234397000
H	5.122634000	4.989776000	7.125223000

[^{CH3}Catechol-H]$^{\bullet}$ (*S* = 1/2)

Zero-point correction= 0.123382 (Hartree/Particle)
Thermal correction to Energy= 0.130977
Thermal correction to Enthalpy= 0.131921
Thermal correction to Gibbs Free Energy= 0.090902
Sum of electronic and zero-point Energies= -421.418388
Sum of electronic and thermal Energies= -421.410793
Sum of electronic and thermal Enthalpies= -421.409849
Sum of electronic and thermal Free Energies= -421.450868

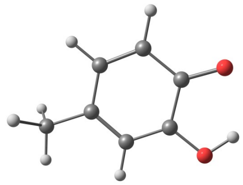

Cartesian Coordinates

C	0.537649000	0.119466000	-4.335976000
C	-0.611203000	-0.355707000	-3.623077000
C	1.689213000	0.491696000	-3.688635000
H	2.559049000	0.851307000	-4.231616000
C	-0.569680000	-0.446157000	-2.226673000
C	1.772769000	0.413272000	-2.252298000
H	-1.430912000	-0.803910000	-1.668554000
C	0.584816000	-0.074935000	-1.548541000
H	0.482456000	0.181700000	-5.420498000
O	0.672890000	-0.144331000	-0.212758000
H	1.580535000	0.171271000	0.009207000
O	2.782720000	0.730461000	-1.560889000
C	-1.842579000	-0.749344000	-4.388688000
H	-1.615126000	-1.546505000	-5.109508000
H	-2.637527000	-1.101595000	-3.724709000
H	-2.228128000	0.099644000	-4.969725000

---

^{CH3}Quinone (*S* = 0)

Zero-point correction= 0.111720 (Hartree/Particle)
Thermal correction to Energy= 0.119333
Thermal correction to Enthalpy= 0.120277
Thermal correction to Gibbs Free Energy= 0.079621
Sum of electronic and zero-point Energies= -420.810222
Sum of electronic and thermal Energies= -420.802609
Sum of electronic and thermal Enthalpies= -420.801664
Sum of electronic and thermal Free Energies= -420.842321

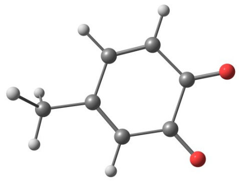

Cartesian Coordinates

C	0.678820000	0.048036000	-4.410101000
C	-0.503898000	-0.450491000	-3.682718000
C	1.762634000	0.502261000	-3.729227000
H	-1.340975000	-0.798903000	-4.284172000
H	2.642989000	0.875080000	-4.247098000
C	-0.583650000	-0.489760000	-2.335361000
C	1.797206000	0.514422000	-2.269547000
H	-1.464587000	-0.861681000	-1.818868000
C	0.539821000	-0.025167000	-1.513483000
O	0.526987000	-0.046533000	-0.290313000
O	2.754734000	0.923971000	-1.622794000
C	0.616666000	0.026249000	-5.908112000
H	1.533294000	0.416666000	-6.358637000
H	0.454254000	-0.998652000	-6.269564000
H	-0.234315000	0.622810000	-6.264876000

^{OCH3}Catechol-H_2 (S = 0)

Zero-point correction= 0.140366 (Hartree/Particle)
Thermal correction to Energy= 0.149314
Thermal correction to Enthalpy= 0.150259
Thermal correction to Gibbs Free Energy= 0.106926
Sum of electronic and zero-point Energies= -497.261619
Sum of electronic and thermal Energies= -497.252670
Sum of electronic and thermal Enthalpies= -497.251726
Sum of electronic and thermal Free Energies= -497.295059

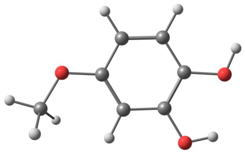

Cartesian Coordinates

C	0.593914000	-0.131319000	-4.388057000
C	-0.597048000	-0.388769000	-3.695052000
C	1.743625000	0.214643000	-3.673719000
C	-0.629271000	-0.297831000	-2.295836000
C	1.714794000	0.306159000	-2.276728000
H	-1.556797000	-0.498771000	-1.768306000
C	0.527061000	0.049217000	-1.594179000
H	0.502163000	0.119918000	-0.508320000
H	0.657896000	-0.191145000	-5.468552000
O	2.909903000	0.655788000	-1.678164000
H	2.806247000	0.697530000	-0.712215000
O	2.899320000	0.462533000	-4.363564000
H	3.599083000	0.687357000	-3.723754000
O	-1.773284000	-0.734877000	-4.303722000

C	-1.797968000	-0.835141000	-5.727474000
H	-1.552903000	0.124925000	-6.199638000
H	-1.107367000	-1.609378000	-6.085484000
H	-2.821151000	-1.115068000	-5.986712000

[^{OCH3}Catechol-H$_2$]$^{•+}$ (S = 1/2)

Zero-point correction= 0.141258 (Hartree/Particle)
Thermal correction to Energy= 0.150068
Thermal correction to Enthalpy= 0.151012
Thermal correction to Gibbs Free Energy= 0.107267
Sum of electronic and zero-point Energies= -497.058519
Sum of electronic and thermal Energies= -497.049709
Sum of electronic and thermal Enthalpies= -497.048764
Sum of electronic and thermal Free Energies= -497.092509

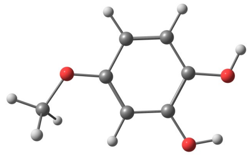

Cartesian Coordinates

C	0.626571000	-0.123874000	-4.427361000
C	-0.561431000	-0.379640000	-3.724133000
C	1.756110000	0.216317000	-3.705481000
C	-0.616088000	-0.290177000	-2.285602000
C	1.702650000	0.303820000	-2.261666000
H	-1.560764000	-0.495530000	-1.792592000
C	0.504144000	0.048361000	-1.568726000
H	0.476418000	0.120412000	-0.485003000
H	0.684512000	-0.185798000	-5.507050000
O	2.860287000	0.634500000	-1.690824000
H	2.796989000	0.686031000	-0.714419000
O	2.901199000	0.463765000	-4.344052000
H	3.617578000	0.688669000	-3.716750000
O	-1.707994000	-0.714522000	-4.289365000
C	-1.807855000	-0.838578000	-5.730746000
H	-1.598369000	0.125993000	-6.201813000
H	-1.117023000	-1.607408000	-6.087569000
H	-2.838716000	-1.136568000	-5.916323000

[^{OCH3}Catechol-H]$^{–}$ (S = 0) (No H-bond)

Zero-point correction= 0.126540 (Hartree/Particle)
Thermal correction to Energy= 0.135315

Thermal correction to Enthalpy= 0.136259
Thermal correction to Gibbs Free Energy= 0.093087
Sum of electronic and zero-point Energies= -496.773212
Sum of electronic and thermal Energies= -496.764436
Sum of electronic and thermal Enthalpies= -496.763492
Sum of electronic and thermal Free Energies= -496.806664

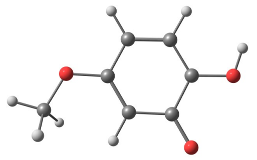

Cartesian Coordinates

C	0.594617000	-0.127181000	-4.387585000
C	-0.590488000	-0.382822000	-3.689271000
C	1.827066000	0.232023000	-3.735891000
C	-0.630676000	-0.297406000	-2.292561000
C	1.740302000	0.311206000	-2.294263000
H	-1.551985000	-0.496531000	-1.752574000
C	0.556355000	0.052913000	-1.618204000
H	0.541965000	0.124233000	-0.528588000
H	0.632324000	-0.191769000	-5.470809000
O	2.906369000	0.658986000	-1.617099000
H	2.708826000	0.673646000	-0.665890000
O	2.910472000	0.462393000	-4.381764000
O	-1.784030000	-0.728905000	-4.303940000
C	-1.806040000	-0.838120000	-5.720221000
H	-1.557884000	0.115612000	-6.205939000
H	-1.116694000	-1.614728000	-6.079080000
H	-2.829632000	-1.118368000	-5.984008000

[^{OCH3}Catechol-H]$^{-}$ (S = 0) (H-bond)

Zero-point correction= 0.127189 (Hartree/Particle)
Thermal correction to Energy= 0.135697
Thermal correction to Enthalpy= 0.136642
Thermal correction to Gibbs Free Energy= 0.093945
Sum of electronic and zero-point Energies= -496.786903
Sum of electronic and thermal Energies= -496.778394
Sum of electronic and thermal Enthalpies= -496.777450
Sum of electronic and thermal Free Energies= -496.820147

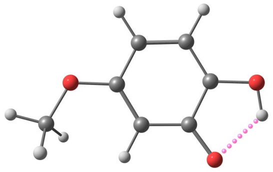

Cartesian Coordinates

C	0.606845000	-0.104903000	-4.394747000
C	-0.577198000	-0.374671000	-3.686901000
C	1.802187000	0.254967000	-3.710610000
C	-0.619661000	-0.298224000	-2.290654000
C	1.722845000	0.322676000	-2.275522000

H	-1.546527000	-0.510408000	-1.764690000
C	0.554130000	0.055654000	-1.587966000
H	0.538780000	0.119617000	-0.500776000
H	0.644350000	-0.162655000	-5.478307000
O	2.901563000	0.672816000	-1.652161000
H	3.502882000	0.784284000	-2.431463000
O	2.944329000	0.524283000	-4.264138000
O	-1.768089000	-0.727143000	-4.303351000
C	-1.789169000	-0.837107000	-5.719446000
H	-1.549539000	0.118392000	-6.206095000
H	-1.093858000	-1.608560000	-6.078147000
H	-2.810450000	-1.125874000	-5.982822000

[^{OCH3}Catecholate]$^{2-}$ (*S* = 0)

Zero-point correction= 0.112890 (Hartree/Particle)
Thermal correction to Energy= 0.121403
Thermal correction to Enthalpy= 0.122347
Thermal correction to Gibbs Free Energy= 0.079586
Sum of electronic and zero-point Energies= -496.246419
Sum of electronic and thermal Energies= -496.237906
Sum of electronic and thermal Enthalpies= -496.236962
Sum of electronic and thermal Free Energies= -496.279723

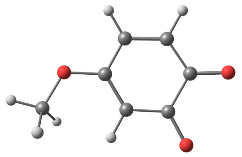

Cartesian Coordinates

C	0.587943000	-0.105826000	-4.373560000
C	-0.614367000	-0.370486000	-3.681671000
C	1.819584000	0.232183000	-3.723875000
C	-0.641892000	-0.301205000	-2.296216000
C	1.803077000	0.324414000	-2.231658000
H	-1.564923000	-0.504851000	-1.753314000
C	0.559484000	0.039173000	-1.608713000
H	0.537799000	0.094781000	-0.516548000
H	0.624478000	-0.156487000	-5.460291000
O	2.871225000	0.642148000	-1.557377000
O	2.895641000	0.451745000	-4.407661000
O	-1.824181000	-0.697357000	-4.324883000
C	-1.809044000	-0.846141000	-5.729470000
H	-1.539746000	0.088687000	-6.244240000
H	-1.117081000	-1.637645000	-6.055523000
H	-2.827513000	-1.125934000	-6.019429000

[^{OCH3}Catechol-H]$^{\bullet}\cdots$H$^{\bullet}$ (*S* = 1)

Zero-point correction= 0.128918 (Hartree/Particle)
Thermal correction to Energy= 0.139164
Thermal correction to Enthalpy= 0.140108
Thermal correction to Gibbs Free Energy= 0.090033
Sum of electronic and zero-point Energies= -497.154021
Sum of electronic and thermal Energies= -497.143774
Sum of electronic and thermal Enthalpies= -497.142830
Sum of electronic and thermal Free Energies= -497.192906

Cartesian Coordinates

C	0.606839000	-0.119338000	-4.452587000
C	-0.555837000	-0.394996000	-3.713064000
C	1.736305000	0.289551000	-3.762276000
C	-0.578836000	-0.260556000	-2.290231000
C	1.760317000	0.441474000	-2.307293000
H	-1.506130000	-0.487541000	-1.772130000
C	0.539033000	0.143785000	-1.606451000
H	0.529851000	0.248905000	-0.525271000
H	0.641119000	-0.217358000	-5.531414000
O	2.842225000	0.820479000	-1.768718000
O	2.882405000	0.571063000	-4.401434000
H	3.515876000	0.832301000	-3.691480000
O	-1.717585000	-0.798014000	-4.258707000
C	-1.811562000	-0.958320000	-5.684216000
H	-1.628004000	-0.006076000	-6.193409000
H	-1.106577000	-1.719098000	-6.036009000
H	-2.834572000	-1.286819000	-5.871427000
H	10.672455000	3.607454000	3.791881000

[^{OCH3}Quinone] ···H$^{\bullet}$ (*S* = 1/2)

Zero-point correction= 0.117338 (Hartree/Particle)
Thermal correction to Energy= 0.127536
Thermal correction to Enthalpy= 0.128480
Thermal correction to Gibbs Free Energy= 0.079124
Sum of electronic and zero-point Energies= -496.547931
Sum of electronic and thermal Energies= -496.537733
Sum of electronic and thermal Enthalpies= -496.536789
Sum of electronic and thermal Free Energies= -496.586144

Cartesian Coordinates

C	0.588330000	-0.116343000	-4.505945000
C	-0.523843000	-0.383190000	-3.757848000
C	1.823435000	0.269976000	-3.857379000
C	-0.527646000	-0.294994000	-2.289177000
C	1.825360000	0.371599000	-2.297443000
H	-1.464268000	-0.528709000	-1.790110000
C	0.570286000	0.058219000	-1.595130000
H	0.568822000	0.124738000	-0.510830000
H	0.592895000	-0.181504000	-5.587989000
O	2.843054000	0.701821000	-1.706718000
O	2.862142000	0.520058000	-4.466224000
O	-1.720366000	-0.748144000	-4.235132000
C	-1.892055000	-0.878111000	-5.658983000
H	-1.711203000	0.081926000	-6.153623000
H	-1.217084000	-1.643379000	-6.056116000
H	-2.929407000	-1.182921000	-5.798834000
H	12.343907000	3.172800000	-6.215252000

[^{OCH3}Catechol-H]$^{\bullet}$ (S = 1/2)

Zero-point correction= 0.128766 (Hartree/Particle)
Thermal correction to Energy= 0.137223
Thermal correction to Enthalpy= 0.138167
Thermal correction to Gibbs Free Energy= 0.094997
Sum of electronic and zero-point Energies= -496.652308
Sum of electronic and thermal Energies= -496.643851
Sum of electronic and thermal Enthalpies= -496.642907
Sum of electronic and thermal Free Energies= -496.686077

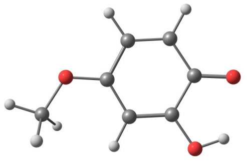

Cartesian Coordinates

C	0.617459000	-0.128452000	-4.436154000
C	-0.564708000	-0.381595000	-3.719811000
C	1.753313000	0.211939000	-3.719664000
C	-0.601335000	-0.291468000	-2.293816000
C	1.763885000	0.316454000	-2.260325000
H	-1.543602000	-0.497374000	-1.794288000
C	0.522584000	0.045895000	-1.584077000

H	0.503410000	0.116649000	-0.500212000
H	0.661164000	-0.192432000	-5.517252000
O	2.852662000	0.634573000	-1.696841000
O	2.918363000	0.466626000	-4.335430000
H	3.552213000	0.679134000	-3.609713000
O	-1.733665000	-0.721529000	-4.292405000
C	-1.812430000	-0.837150000	-5.723135000
H	-1.585261000	0.121423000	-6.202069000
H	-1.131921000	-1.614343000	-6.087126000
H	-2.844291000	-1.120567000	-5.933898000

---

^{OCH3}Quinone (S = 0)

Zero-point correction= 0.117059 (Hartree/Particle)
Thermal correction to Energy= 0.125543
Thermal correction to Enthalpy= 0.126487
Thermal correction to Gibbs Free Energy= 0.083676
Sum of electronic and zero-point Energies= -496.046329
Sum of electronic and thermal Energies= -496.037846
Sum of electronic and thermal Enthalpies= -496.036902
Sum of electronic and thermal Free Energies= -496.079713

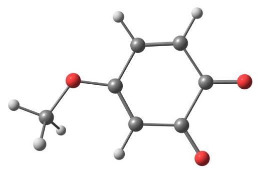

Cartesian Coordinates

C	0.622109000	-0.129060000	-4.464254000
C	-0.523473000	-0.371816000	-3.759683000
C	1.839007000	0.230898000	-3.768149000
C	-0.582555000	-0.283386000	-2.292223000
C	1.785641000	0.321411000	-2.208418000
H	-1.543519000	-0.494337000	-1.830536000
C	0.496308000	0.041261000	-1.555539000
H	0.455113000	0.105968000	-0.471893000
H	0.666232000	-0.192559000	-5.545527000
O	2.790315000	0.615281000	-1.577598000
O	2.903473000	0.466902000	-4.336816000
O	-1.707949000	-0.710863000	-4.283627000
C	-1.824508000	-0.840854000	-5.712943000
H	-1.605776000	0.114392000	-6.201517000
H	-1.148972000	-1.619841000	-6.081404000
H	-2.860959000	-1.126202000	-5.894300000

References

1 H. -C. Liang, E. Kim, C. D. Incarvito, A. L. Rheingold and K. D. Karlin, *Inorg. Chem.*, 2002, **41**, 2209.

2 R. A. Ghiladi, R. M. Kretzer, I. Guzei, A. L. Rheingold, Y.-M. Neuhold, K. R. Hatwell, A. D. Zuberbuhler and K. D. Karlin, *Inorg. Chem.*, 2001, **40**, 5754.

3 I. Garcia-Bosch, S. M. Adam, A. W. Schaefer, S. K. Sharma, R. L. Peterson, E. I. Solomon, and K. D. Karlin, *J. Am. Chem. Soc.*, 2015, **137**, 1032.

4 S. M. Adam, I. Garcia-Bosch, A. W. Schaefer, S. K. Sharma, M. A. Siegler, E. I. Solomon and K. D. Karlin, *J. Am. Chem. Soc.* 2017, **139**, 472.

5 S. Cambré, W. Wenseleers and E. Goovaerts, *J. Phys. Chem. C* 2009, **113**, 13505.

6 D. T. Petasis, M. P. Hendrich, Chapter Eight - Quantitative Interpretation of Multifrequency Multimode EPR Spectra of Metal Containing Proteins, Enzymes, and Biomimetic Complexes. In Methods in Enzymology, Qin, P. Z.; Warncke, K., Eds. Academic Press: 2015; Vol. 563, pp 171-208.

7 A. P. Golombek and M. P. Hendrich, Quantitative analysis of dinuclear manganese(II) EPR spectra. *Journal of Magnetic Resonance* 2003**, 165**, 33-48.

8 J. A. Weil, J. R. Bolton and J. E. Wertz, Electron Paramagnetic Resonance: Elementary Theory and Practial Applications. John Wiley & Sons, Inc.: New York.

9 A. Abragam and B. Bleaney, Electron Paramagnetic Resonance of Transition Ions (International Series of Monographs on Physics). 1970; p 912 pp.

10 R. L. Peterson, J. W. Ginsbach, R. E. Cowley, M. F. Qayyum, R. A. Himes, M. A. Siegler, C. D. Moore, B. Hedman, K. O. Hodgson, S. Fukuzumi, E. I. Solomon and K. D. Karlin, *J. Am. Chem. Soc.* 2013, **135**, 16454.

11 X.-Q. Zhu, C.-H. Wang, and H. Liang, *J. Org. Chem.* 2010, **75**, 7240.

12 M. J. Frisch, G. W. Trucks, H. B. Schlegel, G. E. Scuseria, M. A. Robb, J. R. Cheeseman, G. Scalmani, V. Barone, B. Mennucci, G. A. Petersson, H. Nakatsuji, M. Caricato, X. Li, H. P. Hratchian, A. F. Izmaylov, J. Bloino, G. Zheng, J. L. Sonnenberg, M. Hada, M. Ehara, K. Toyota, R. Fukuda, J. Hasegawa, M. Ishida, T.

Nakajima, Y. Honda, O. Kitao, H. Nakai, T. Vreven, J. A. Montgomery Jr., J. E. Peralta, F. Ogliaro, M. Bearpark, J. J. Heyd, E. Brothers, K. N. Kudin, V. N. Staroverov, R. Kobayashi, J. Normand, K. Raghavachari, A. Rendell, J. C. Burant, S. S. Iyengar, J. Tomasi, M. Cossi, N. Rega, J. M. Millam, M. Klene, J. E. Knox, J. B. Cross, V. Bakken, C. Adamo, J. Jaramillo, R. Gomperts, R. E. Stratmann, O. Yazyev, A. J. Austin, R. Cammi, C. Pomelli, J. W. Ochterski, R. L. Martin, K. Morokuma, V. G. Zakrzewski, G. A. Voth, P. Salvador, J. J. Dannenberg, S. Dapprich, A. D. Daniels, O. Farkas, J. B. Foresman, J. V. Ortiz, J. Cioslowski and D. J. Fox, *Gaussian 09 (Revision A.02)*, Gaussian, Inc., Wallingford CT, 2009.

13 E. Nam, P. E. Alokolaro, R. D. Swartz, M. C. Gleaves, J. Pikul and J. A. Kovacs, *Inorg. Chem.* 2011, **50**, 1592–1602.

14 A. Steckel and G. Schlosser, *Molecules*, 2019, **24**, 611.
